# Supplementary material for: A pragmatic randomised controlled trial of ‘PhysioDirect’ telephone assessment and advice services for patients with musculoskeletal problems: economic evaluation
Source: BMJ Open. 2013 Oct 1;3(10):e003406. doi: 10.1136/bmjopen-2013-003406 (PMC3796275; doi:10.1136/bmjopen-2013-003406)
Supplement: Author's manuscript [file bmjopen-2013-003406.draft_revisions.pdf]

# A pragmatic randomised controlled trial of 'PhysioDirect' telephone assessment and advice services for patients with musculoskeletal problems: economic evaluation

| | |
|---|---|
| Journal: | *BMJ Open* |
| Manuscript ID: | bmjopen-2013-003406 |
| Article Type: | Research |
| Date Submitted by the Author: | 13-Jun-2013 |
| Complete List of Authors: | Hollinghurst, Sandra; University of Bristol, Community Based Medicine<br>Coast, Joanna; University of Birmingham, Department of Health Economics<br>Busby, John; University of Bristol,<br>Bishop, Annette; Keele University,<br>Foster, Nadine; Keele University, Primary Care Musculoskeletal Research Centre<br>Franchini, Angelo; Imperial College, London,<br>Grove, Sean; Bristol Community Health,<br>Hall, Jeanette; Bristol Community Health,<br>Hopper, Cherida; University of Bristol,<br>Kaur, Surinder; University of Bristol,<br>Montgomery, Alan; University of Nottingham,<br>Salisbury, Chris; University of Bristol, Academic Unit of Primary Health Care |
| <b>Primary Subject Heading</b>: | Health economics |
| Secondary Subject Heading: | General practice / Family practice |
| Keywords: | economic evaluation, physiotherapy, telehealth, PRIMARY CARE, costs & cost analysis |
| | |

SCHOLARONE™ Manuscripts

# A pragmatic randomised controlled trial of 'PhysioDirect' telephone assessment and advice services for patients with musculoskeletal problems: economic evaluation

Sandra Hollinghurst, Joanna Coast, John Busby, Annette Bishop, Nadine E Foster, Angelo Franchini, Sean Grove, Jeanette Hall, Cherida Hopper, Surinder Kaur, Alan A Montgomery, Chris Salisbury

Sandra Hollinghurst, PhD. Senior Lecturer in Health Economics[1]
Joanna Coast, PhD. Professor of Health Economics[2]
John Busby, MSc. Research Assistant in Health Economics[1]
Annette Bishop, PhD. Research Physiotherapist[3]
Nadine E Foster, D.Phil. NIHR Professor of Musculoskeletal Health in Primary Care[3]
Angelo Franchini, MSc. Research Associate[4]
Sean Grove, MSc. Clinical Lead Physiotherapy[5]
Jeanette Hall, Grad Dip Phys. Operational lead for Outpatient Physiotherapy[5]
Cherida Hopper, PhD. Trial Manager[1]
Surinder Kaur, BSc. Research Associate[1]
Alan A Montgomery, PhD. Professor of Medical Statistics and Clinical Trials[6]
Chris Salisbury, MD. Professor of Primary Health Care[1]

[1]Centre for Academic Primary Care, School of Social and Community Medicine, University of Bristol, UK
[2]Health Economics Unit, School of Health & Population Sciences, University of Birmingham, UK
[3]Arthritis Research UK Primary Care Centre, Primary Care Sciences, Keele University, UK
[4] Imperial Clinical Trials Unit, School of Public Health Medicine, Imperial College, London, UK
[5]Musculoskeletal Outpatient Department, Bristol Community Health, UK
[6] Nottingham Clinical Trials Unit, Queen's Medical Centre, Nottingham, UK

**Correspondence:**
Sandra Hollinghurst, School of Social and Community Medicine, University of Bristol, Canynge Hall, 39 Whatley Road, Bristol BS8 2PS
s.p.hollinghurst@bristol.ac.uk
0117 331 3901

Key Words: economic evaluation; physiotherapy; telehealth; primary care; cost & cost analysis

For peer review only

# A pragmatic randomised controlled trial of 'PhysioDirect' telephone assessment and advice services for patients with musculoskeletal problems: economic evaluation


## ABSTRACT

### Objectives

To compare the cost-effectiveness of PhysioDirect with usual physiotherapy care for patients with musculoskeletal problems.

### Design

(i) Cost-consequences comparing cost to the NHS, to patients, and the value of lost productivity with a range of outcomes. (ii) Cost-utility analysis comparing cost to the NHS with Quality Adjusted Life Years (QALYs)

### Setting

Four physiotherapy services in England

### Participants

Adults (18+) referred by their general practitioner or self-referred for physiotherapy.

### Interventions

PhysioDirect involved telephone assessment and advice followed by face-to-face care if needed. Usual care patients were placed on a waiting list for face-to-face care.

### Primary and secondary outcome measures

Primary clinical outcome: physical component summary measure from the SF-36v2 at six months.

Secondary outcomes included: Measure Yourself Medical Outcomes Profile; a Global Improvement Score; response to treatment; patient satisfaction; waiting time.

Outcome for the cost-utility analysis: QALYs

### Results

2249 patients took part (1506 PhysioDirect; 743 usual care).


For peer review only

(i) Cost-consequences: there was no evidence of a difference between the two groups in the cost of physiotherapy, other NHS services, personal costs or value of time off work. Outcomes were also similar.

(ii) Cost-utility analysis based on complete cases (n=1,272). Total NHS costs, including the cost of physiotherapy were higher in the PhysioDirect group by £19.30 (95% CI: -£37.60 to £76.19) and there was a QALY gain of 0.007 (95% CI: -0.003 to 0.016). The incremental cost-effectiveness ratio was £2,889 and the net monetary benefit at λ=£20,000 was £117 (95% CI: -£86 to £310)

**Conclusions**

PhysioDirect may be a cost-effective alternative to usual physiotherapy care, though only with careful management of staff time. Physiotherapists providing the service must be more fully occupied than was possible under trial conditions: consideration should be given to the scale of operation, opening times of the service, and flexibility in the methods used to contact patients.

For peer review only

**Article Summary**

Article focus

- What is the cost of providing PhysioDirect telephone assessment and advice, followed by face-to-face care if needed, to adults with musculoskeletal pain problems, compared with usual physiotherapy care?
- What are the patient benefits of PhysioDirect compared with usual care?
- Is PhysioDirect a cost-effective alternative to usual care for primary care patients with musculoskeletal conditions?

Key messages

- There was very little difference between the two groups in terms of cost or outcomes
- PhysioDirect has potential to be a cost-effective alternative to usual care but requires careful management of staff resources

Strengths and limitations of this study

- Findings are based on a large sample of patients with a wide range of musculoskeletal problems based in diverse locations
- The study takes a broad perspective, including the health care provider, patients, and a valuation of lost productivity
- Physiotherapists were constrained by trial conditions and were underutilised

For peer review only

## INTRODUCTION

There is a trend to explore the use of new technology in the delivery of health care, particularly the use of telephone assessment and triage, as for example in NHS Direct.[1] These services aim to better manage patient demand, and research has shown that telephone based services can be safe, clinically accurate, cost-effective, acceptable to patients, and reduce the workload of clinicians,[2-5] although there have been some concerns about using telephone triage in patients presenting with acute health problems.[6]

Musculoskeletal pain problems are one of the most common causes of disability. Over a quarter of all patients registered in general practice will consult at least once for a musculoskeletal problem each year,[7,8] with musculoskeletal pain accounting for around 15% of all GP consultations.[9] This high prevalence of musculoskeletal problems results in large direct and indirect healthcare costs,[10] estimated for low back pain alone at £10,668 million for the UK in 1998[11] and rising substantially since then.[12] Most patients are managed with advice and analgesia but many are referred to physiotherapists, with 1.23 million new referrals each year from GPs and 4.4 million in total.[13] Ensuring timely access to physiotherapy has long been an issue within the UK NHS, with waiting times of more than four months in some areas. Patients may suffer unnecessary pain and disability, and there are high productivity losses: for example, back pain accounts for some 120 million days of certified absence from work each year.[11] Delay may also cause NHS inefficiencies on the one hand as some patients recover and do not attend their physiotherapy appointment when it finally arrives, whilst on the other, some patients continue to access more expensive forms of treatment whilst awaiting their appointment.

In response to these problems, physiotherapy services have drawn on the new service models and a range of 'PhysioDirect' services have been developed. These vary in format though they commonly involve a physiotherapist assessing a patient's musculoskeletal pain problem over the telephone,

For peer review only

sometimes supported by computerised assessment templates, offering tailored, self-management advice supplemented by written advice sent by post. Alternatively if the assessment findings suggest that face-to-face care is needed this is arranged, and patients who are initially managed by telephone advice can phone back for further advice and/or face-to-face treatment.[14] There is, however, limited evidence about the costs and benefits of this approach within physiotherapy. Local evaluations and non-randomised studies suggest that these services may be popular with patients[15-17] and diagnoses made by physiotherapists over the telephone are comparable to diagnoses reached in face-to-face assessments[18-20] although there is some concern that the experience of the physiotherapist providing the telephone assessment might be important.[18,21]

In particular there is no information about the costs or cost-effectiveness of PhysioDirect services, despite: (i) a major underlying rationale for their development being to generate greater efficiency in the use of resources; and (ii) a ready presumption that telephone-based services result in lower costs (by assuming that services better use physiotherapy time, use less costly telephone consultations and reduce rates of appointment non-attendance). Without such evidence, preferably generated alongside high quality primary evidence obtained using rigorous study designs, it remains unclear whether such services should be more widely implemented. This paper reports the results of an economic evaluation conducted alongside a randomised controlled trial powered to generate evidence on whether PhysioDirect services for primary care patients with musculoskeletal problems produce equivalent outcomes to usual face-to-face services.

## METHOD

### Study design

We conducted an economic evaluation alongside a randomised controlled trial to establish the cost-effectiveness of PhysioDirect compared with usual care based only on face-to-face treatment over a

For peer review only

period of 6 months. The trial and its clinical findings have been reported in full elsewhere.[14,22] The primary perspective for the economic evaluation was the health and social care provider, as that is of greatest interest to UK policy makers.[23] However, the nature of the intervention suggests there could be an impact on patients' costs, and as it is known that musculoskeletal conditions account for a considerable amount of time off work[10,11] we included these perspectives in the analysis. We used a cost-consequences approach to compare cost from all three perspectives (health care provider, patients and carers, lost productivity) with a range of clinical outcomes and used a cost-utility analysis to compare cost to the National Health Service (NHS) with Quality-Adjusted Life-Years (QALYs).

**Setting and participants**

We recruited adults aged 18 and over from four community physiotherapy services in England – Bristol, Somerset, Stoke-on-Trent, and Cheshire – which provided diversity in terms of socio-economic status and a mix of urban and rural communities. All patients referred by their general practitioner (GP), or who referred themselves, for physiotherapy for a non-urgent musculoskeletal problem were invited to take part. Patients were randomised to PhysioDirect or usual care on a two to one basis to increase the chances of the PhysioDirect service being fully utilised.

**Interventions**

The intervention has been reported in detail elsewhere[14,22]. Patients randomised to the PhysioDirect service received an invitation to telephone a senior (band 6 or above) specially trained physiotherapist, who assessed their musculoskeletal problem with the aid of previously developed computerised templates.[22] These templates were provided by Huntingdonshire Primary Care Trust, which has been operating a similar service since 2001. Patients were then sent appropriate advice leaflets about self-management and exercises to try at home, and invited to phone again and/or

For peer review only

make a face-to-face appointment if necessary. Patients randomised to usual care were put on the usual service waiting list for face-to-face assessment and treatment.

**Outcome measures**

We used the EQ-5D-3L[24], valued using the UK tariff,[25] to estimate QALYs gained for the cost-utility analysis. The primary outcome for the trial was the physical component summary (PCS) measure from the SF-36v2 questionnaire[26] and secondary clinical outcomes included: the Measure Yourself Medical Outcomes Profile (MYMOP);[27] a Global Improvement Score – a single question about overall improvement; a composite measure of response to treatment including pain, function and overall improvement (OMERACT OARSI);[28] patient satisfaction; and waiting time to first treatment advice from a physiotherapist. All outcomes (except the global improvement score and waiting time to first treatment advice) were measured at baseline, 6 weeks and 6 months and were obtained from a self completed questionnaire administered at these three time points.

**Resource use**

The analysis was based on costs related to the reason for which the patient was referred to the physiotherapy service. We identified relevant resources in discussion with participating physiotherapists and service managers. Direct costs to the health care provider included: cost of initial and follow-up physiotherapy consultations; primary and community consultations; hospital care; and prescribed medication. Patient and carer costs included: telephone calls to the PhysioDirect service; travel; over the counter medication; prescription costs; private therapy and purchase of equipment; extra domestic help; and loss of earnings. Lost productivity was estimated separately in relation to time off work to attend physiotherapy appointments and time off because of the musculoskeletal condition itself.

For peer review only



For peer review only - http://bmjopen.bmj.com/site/about/guidelines.xhtml

Patient level data about all physiotherapy appointments and consultations were recorded either automatically by computer or by the physiotherapist treating the patient. For those in the intervention group, the PhysioDirect assessment software recorded which physiotherapist conducted each telephone call, and the duration of each call. In addition to the time logged on to the system physiotherapists had to carry out administrative activities following each telephone call, such as collating information to send to the patient by post. The time spent on these activities was estimated from information available at one site (Bristol) where manual recording of the entire encounter supplemented the electronic recording.

Physiotherapists assigned to the PhysioDirect service were required to be available throughout the time the service was 'open' but they were not usually fully engaged in dealing with PhysioDirect patients during these hours. We conducted an observational time and motion study at each of the four sites to determine how they occupied their non-PhysioDirect contact time in order to apportion costs appropriately. Time and motion data were collected at points in the study when the sites were expected to be fully operational, and across a mix of day, time of day, and location. The capital costs required to run a telephone services are potentially less than for a face-to-face service. Each site provided information about space and equipment required to run their telephone service and we used this to estimate an overall percentage reduction of capital costs for these compared with a standard face-to-face service.

Data about all face-to-face appointments were recorded routinely. These data included the length of appointment, the grade of the physiotherapist seen, and information about missed appointments.

Information about other NHS resource use was collected, where possible, from general practice records and supplemented by information gained directly from patients. General practice notes were scrutinised for patient level data on primary care consultations and prescribed medication. We

For peer review only

included all consultations at which the musculoskeletal condition for which the patient was referred to physiotherapy was mentioned and these were recorded by type of consultation (for example, face-to-face, telephone, out of hours, home visit) and by type of professional seen (for example, GP, nurse). It was not feasible to distinguish between medication prescribed for the condition for which the patient was referred for physiotherapy and any other musculoskeletal problem so we included all medication of a potentially relevant type, defined using British National Formulary (BNF)[29] coding. These were: Analgesics (chapters 4.7.1 – 4.7.2); Non-steroidal anti-inflammatory drugs (10.1.1); Local corticosteroid injections (10.1.2.2); and Drugs for the relief of soft-tissue inflammation (10.3).

A questionnaire was administered to participants at 6 weeks and 6 months after randomisation to obtain resource use data not available elsewhere. These included information about hospital care related to the condition for which the patient was referred to physiotherapy: visits to Accident & Emergency, outpatient appointments, and inpatient stays. Information about personal expenditure relevant to the patient's musculoskeletal condition was also gained from the questionnaire at 6 weeks and 6 months. We asked about the cost of travel to physiotherapy and other health care appointments, expenditure on over the counter medication, prescription costs, use of private therapies and their cost, expenditure on equipment or devices, and extra help at home. In addition, participants were asked about any time off work, and the associated loss of earnings, because of their condition or to attend health care appointments relating to the condition including usual care physiotherapy and PhysioDirect.

**Valuation of resource use**

Table 1 gives the unit costs and sources used to value the health care resources. We used Curtis[30] to value primary and community health care and Department of Health reference costs[31] for all hospital-based care. The cost of prescribed medication was estimated from that published in the BNF,[29] adjusted to allow for the discount available to the NHS, and the professional fee and



For peer review only

container allowance in accordance with the Drug Tariff for England.[32] Personal expenditure was reported directly by the participants, the exception being travel by car, which was reported as mileage and costed using the AA schedule of motoring costs.[33] Time off work was valued using the median gross weekly earnings by age and sex.[34]

*Table 1 here*

The cost of face-to-face physiotherapy consultations was estimated by adapting the methods of Curtis[30] to obtain a different unit cost for each band of staff at each site. National median pay rates, by band,[36] were adjusted to allow for National Insurance, superannuation, and overheads, as per Curtis, then further adjusted to allow for band and site specific non-contact time. Information about the proportion of time physiotherapists on each grade typically spend in direct contact with patients was provided by the four physiotherapy service managers. This provided us with a cost per hour for each band of staff at each site.

The unit cost of physiotherapists working in the PhysioDirect service was estimated in a similar way, but allowing for the reduced cost of capital and overheads; information from the site managers indicated this to be about 50%. To obtain a cost per hour of telephone contact we used information from the computerised records of the PhysioDirect service, which identified the proportion of time spent by physiotherapists actually dealing with PhysioDirect patients. We then combined this with data from the time and motion study, which identified activities undertaken during non-contact time, for example, administration relating to face-to-face appointments or general administration, to give a cost per hour for each band of staff at each site.

All costs were valued in £ sterling at 2009 prices, adjusted for inflation where necessary.[30]


For peer review only - http://bmjopen.bmj.com/site/about/guidelines.xhtml

**Data analysis**

We investigated the amount of each resource used by patients in each group using frequencies, means and medians. Mean total cost per participant was derived by combining resource use with unit costs.

QALYs were derived from responses to the EQ-5D-3L at baseline, 6 weeks and 6 months using valuations from the UK general population.[25] These values, representing health-related quality of life on a scale between zero (death) and 1 (best imaginable health), were used to compute QALYs experienced over the 6 month period using the area under the curve approach and adjusting for any difference between the groups at baseline.[37]

A cost-consequences matrix was constructed using all available data. We compared costs from all three perspectives (health care provider, patients and carers, lost productivity) with the SF-36v2 PCS, MYMOP, Global Improvement Score, OMERACT OARSI, patient satisfaction, waiting time, and QALYs.

The cost-utility analysis was carried out using data on patients for whom we had complete NHS cost and QALY data. An incremental cost-effectiveness ratio (ICER) was constructed, comparing the difference in mean total cost per patient with mean difference in QALYs. Uncertainty around the ICER was captured by replicating it 5000 times using the bootstrapping technique, estimating a confidence interval around the net monetary benefit, and constructing a cost-effectiveness acceptability curve.

We used the multiple imputation by chained equation procedure to address the issue of missing cost and EQ-5D data.[38] This technique uses a regression model to estimate missing values from known values. In addition to cost and EQ-5D-3L variables the imputation model also included randomisation


For peer review only - http://bmjopen.bmj.com/site/about/guidelines.xhtml

group, age, sex, and SF36v2 PCS. Stata v12[39] was used to generate five datasets using 10 switching procedures.

Discounting was not carried out because the analysis was restricted to costs and outcomes over a period of less than a year. All analyses were conducted using Microsoft Excel and Stata v12.[39]

**Sensitivity Analyses**

We addressed three areas of uncertainty using one/two-way sensitivity analyses. First, we estimated the cost of running the PhysioDirect service if it was operating at full capacity. It is likely that this was not achieved during the trial because of: low demand due to exclusions and non-participation in the trial; inflexible staffing levels to ensure consistency throughout the trial period; and the 'one-way' system generally used, where physiotherapists waited for patients to call them but did not routinely contact patients themselves (notwithstanding some limited use of answer-machines). Data from the Bristol service, which continued to operate beyond the trial period and was then able to tailor staffing levels to demand, were used to estimate the cost of running a more 'efficient but feasible' PhysioDirect service once the trial had ended.

The second area of uncertainty addressed hospital costs. Patients in the trial were recruited from primary care and for these, use of secondary care is infrequent but relatively expensive and this can have a disproportionate effect on mean total cost. We tested this by excluding hospital costs from the total.

The third and fourth sensitivity analyses used the imputed missing data as described above first with data collected during the trial and second by combining with results of the first sensitivity analysis, mimicking an 'efficient but feasible' service.

For peer review only

## RESULTS

A total of 2,249 patients took part in the trial, 1506 allocated to PhysioDirect and 743 to usual care. The mean age was 60, with slightly more females than males (60% vs 40%); they were overwhelmingly white (97%), just over half (60%) were employed and all but a few were referred for physiotherapy by their GP. Nearly all participants (2,223=99%) gave permission to access their GP notes to obtain data about primary care encounters and prescribed medication. 81% returned questionnaires at both 6 weeks and 6 months though not all participants completed all sections at both time points. We had complete NHS cost and QALY data for 840 (56%) PhysioDirect and 432 (58%) usual care participants.

### Resource use

Table 2 gives information about the different types of physiotherapy consultations by patients in each group. Of the 1506 patients in the PhysioDirect group, 39% (586) were managed solely over the telephone and only 46% (695) had any face-to-face consultations. In total, patients in the usual care group had, on average, 0.38 (95% CI: 0.12 to 0.63) more consultations than those in the PhysioDirect group and the mean total duration of all consultations was 20 minutes longer (95% CI: 12 to 28).

*Table 2 here*

Tables 3 and 4 give information about NHS and personal resource use. Just over a third of patients (35%) had a GP consultation during the 6 months and 40% received a prescription for musculoskeletal pain-related medication. There was very little difference between the two groups in terms of health care use and the only important difference in personal expenditure was travel to physiotherapy appointments.

For peer review only

*Tables 3 and 4 here*

**Costs and consequences**

Table 5 summarises the mean cost per patient, by group, for each category of cost. All available data are included giving variable denominators for each category. Comparing the two groups, there are small differences in cost in some categories but for most of these the confidence intervals indicate there is no evidence of a difference between the groups.

*Table 5 here*

Table 6 combines the results of the cost analysis with the full range of primary and secondary outcomes, including QALYs.

*Table 6 here*

Results are presented for all available data, with cost categories combined. Again, denominators vary within the table and they also differ from those in table 5 because sub-categories have been collapsed. There was no evidence of a difference in the primary clinical outcome (the SF36v2 PCS) between the groups, suggesting that PhysioDirect led to similar outcomes as usual physiotherapy care. Patients in the PhysioDirect group had their first assessment and telephone advice 27 days earlier than those in the usual care group, however patient satisfaction was slightly lower in those receiving PhysioDirect. QALYs were higher in the PhysioDirect group by 0.009, which equates to about 3.3 extra days of full health over a year.

**Cost-utility analysis**

For peer review only



For peer review only - http://bmjopen.bmj.com/site/about/guidelines.xhtml

The cost-utility analysis presented in Table 7 uses complete cases, that is, we include only those patients for whom we had complete NHS cost and QALY data: 432 (58%) from the usual care group and 840 (56%) from PhysioDirect. The small extra cost of caring for patients in the PhysioDirect group was compensated for by the extra QALY gain, giving an incremental cost-effectiveness ratio of £2,889. If society is willing to pay £20,000 per QALY[23] the net monetary benefit is £117 (95% CI -£96 to £310) and there is 0.88 probability that the intervention is cost-effective. This is illustrated in the cost-effectiveness acceptability curve in figure 1.

*Table 7 here*

*Figure 1 here*

**Sensitivity Analysis**

The results of the four sensitivity analyses are shown in table 8 and figure 2. Scenario (1) indicates the potential cost-effectiveness of a more efficient PhysioDirect service. During the trial PhysioDirect clinic opening hours, physiotherapists spent about 35% of their time on the phone or dealing with directly related administration; in Bristol after the trial, this was increased to 57%. Under this scenario, the cost per patient in the PhysioDirect group was £14.53 less than under trial conditions and £2.11 less per patient in the usual care group. The ICER is therefore lower, at £1,045, the NMB correspondingly higher at £127 ($\lambda$=£20,000). At low levels of $\lambda$, the probability of PhysioDirect being cost-effective under this scenario is higher than with the base case, though at $\lambda$=£20,000 it reaches a similar value (see figures 1 and 2).

*Table 8 here*

*Figure 2 here*

For peer review only

The effect of removing hospital costs from the analysis is shown in sensitivity analysis (2). Hospital costs accounted for 75% of all NHS costs yet only 19% (n=252) participants reported using any secondary care. Hospital use was evenly divided between the two groups so removing these from the analysis made very little difference to incremental analysis.

The effect of imputing missing NHS cost and QALY data is explored in sensitivity analysis (3). Using these data the cost of the interventions is lower but this is offset by higher NHS costs, giving a higher mean total cost in both groups, by £21.41 in the usual care group and £6.58 in the PhysioDirect group. QALYs using imputed data are lower, by 0.005 in the usual care group and by 0.010 in the PhysioDirect group. The net effect is a reduction of both incremental cost and incremental QALYs, giving an ICER of £2,260. Uncertainty around the ICER is reduced, as seen by the flatter CEAC in figure 2.

Sensitivity analysis (4) combines analyses (1) and (3) by using imputed cost data in the 'efficient service' scenario. The results suggest a possible cost saving of £6.02 per patient; the probability that the service is cost-effective at λ=£20,000 is 0.72.

## DISCUSSION

### Statement of principal findings

The results of this economic evaluation suggest that PhysioDirect services for patients with musculoskeletal problems require careful management if they are to be a cost-effective alternative to usual physiotherapy care. There was very little difference between the two groups in terms of either outcomes or costs, and the finding that PhysioDirect is cost-effective is based on evidence that it provides very slightly greater QALY benefits at very slightly greater cost.

For peer review only

Clearer cost savings were observed in the sensitivity analysis that replicated the post-trial service, once greater flexibility in working arrangements was implemented. Without the restrictions of a trial environment staffing was adjusted to meet the anticipated demand, a call-back service was employed which accommodated fluctuations in activity during each session, referrals added to the system were adjusted regularly to reflect actual staffing and the number of patients waiting for a call-back and a higher throughput of patients led to greater economies of scale. These changes ensured physiotherapists within the PhysioDirect service spent a higher proportion of their PhysioDirect clinic time on the telephone with patients. There was no evidence of a difference between PhysioDirect and usual care in cost to patients and their families, or to society through the costs of lost production.

**Strengths and weaknesses of the study**

The study has a number of strengths. It is the first study assessing the cost-effectiveness of a PhysioDirect service including a large sample of patients with a wide range of musculoskeletal problems based across a number of locations.[14] It uses a rigorous study design and conforms to CONSORT guidelines.[40] Follow up from participating patients was good, a high proportion of the resource use data were collected from GP records, and there was collection of resource use information outside of the main health service perspective. Nevertheless, there are also limitations. The proportion of eligible individuals consenting to participate in the trial was only 50%.[22] Further, a particular difficulty in conducting economic evaluation with new service developments is ensuring that they are fully utilised.[41] Although there was a clear run-in period prior to data collection for the trial (ranging from 4 to 12 weeks in each of the four sites), to ensure that services were operating as well as they could, and a 2:1 randomisation ratio in favour of PhysioDirect was used, there was still considerable underutilisation of the new service. This was ameliorated by including a more fully utilised service within the sensitivity analysis. Finally, because the differences in both costs and effects are small, there is still some uncertainty around the findings.

For peer review only

**The meaning of the study and implications for policy makers**

If the aim of health services is to achieve maximum health gain from an investment in health care, then PhysioDirect has a high probability of being more cost-effective than usual physiotherapy care. This research also, however, suggests that it cannot be assumed that PhysioDirect will reduce costs, although it could potentially do so if PhysioDirect services are managed efficiently. Both the costs and cost-effectiveness of services will depend on the productivity of physiotherapist time. If physiotherapists are able to use most of their time dealing directly with patients during sessions when they are available on the telephone, then the service will be less costly. This is most likely to be achieved by operating a call-back service and/or by operating the service on a large scale to even out fluctuations in demand. Further efficiencies may also be achieved if these services are, in the future, provided in conjunction with direct access for patients (rather than following referral from another health care professional), given that patients who self-refer are likely to contact the service with musculoskeletal problems of shorter duration,[42] and such patients may be particularly appropriate for the initial assessment and advice provided by a PhysioDirect service. More generally, the study has broader implications for telephone services, particularly around the implicit assumption that such services will inevitably be money saving. Here this assumption was found to be false, largely because the physiotherapists' time was underutilised during PhysioDirect clinic hours. Thus, for all such services, it will be important for policy makers to ensure that easy assumptions about the costs of these services are properly assessed in relation to factors such as how efficiently the service is run, and what proportion of patients are subsequently invited for face to face care following an initial telephone call.

**Unanswered questions and future research**

As services evolve, further research should explore the costs and benefits of PhysioDirect under different scenarios. These might include: comparing different skill levels of staff operating the

For peer review only

service; the inclusion of patient self-referrals in addition to GP referrals; the use, or otherwise, of computerised support in assessing the patient; the extension to internet services (possibly combined with cameras);[43] and the use of mobile 'smartphone' technology, for example in rapid assessment of musculoskeletal injuries. In particular, however, it will be important to assess the costs and benefits of services once they are more established and provided on a wider scale. The costs and benefits of telehealth more generally need further exploration in relation to their cost effectiveness particularly given the negative findings of the Whole Systems Demonstrator project evaluating telehealth support and treatment for patients with long term conditions.[44] It would be helpful to identify those characteristics that are likely to make services both more cost-effective and less costly.

For peer review only

## References


1. Munro J, Nicholl, J, O'Cathain A et al. Impact of NHS Direct on demand for immediate care: observational study. *Br Med J* 2000;321:150-153.

2. Lattimer V, Sassi F, George S et al. Cost analysis of nurse telephone consultation in out of hours primary care: evidence from a randomised controlled trial. *Br Med J* 2000;320:1053-1057.

3. Lattimer V, George S, Thompson F et al. Safety and effectiveness of nurse telephone consultation in out of hours primary care: randomised controlled trial. The South Wiltshire Out of Hours Project (SWOOP) Group. *Br Med J* 1998;317:1054-1059.

4. Pinnock H, Bawden R, Proctor S et al. Accessibility, acceptability, and effectiveness in primary care of routine telephone review of asthma: pragmatic, randomised controlled trial. *Br Med J* 2003;326:477-479.

5. Bunn F, Byrne G, Kendall S. Telephone consultation and triage: effects on health care use and patient satisfaction.*Cochrane Database of Systematic Reviews.(4):CD004180, 2004.* 2004;CD004180.

6. McKinstry B, Watson P, Pinnock H et al. Telephone consulting in primary care: a triangulated qualitative study of patients and providers. *Br J Gen Pract* 2009;59:e209-e218.

7. Picavet HS, Schouten JS. Musculoskeletal pain in the Netherlands: prevalences, consequences and risk groups, the DMC(3)-study. *Pain* 2003;102:167-178.

8. Jordan K, Ong BN, Croft P. Previous consultation and self reported health status as predictors of future demand for primary care. *J Epidemiol Commun Health* 2003;57:109-113.

9. McKormick A, Fleming D, and Charlton J *Morbidity statistics from general practice. Fourth national study 1991-1992 Serise MB5 no.3*. London: 1995.


For peer review only

10. Andersson GB. Epidemiological features of chronic low-back pain. *Lancet* 1999; 354: 581-585.

11. Maniadakis N, Gray A. The economic burden of back pain in the UK. *Pain* 2000;84:95-103.

12. National Institute for Health and Clinical Excellence *Low back pain: early management of persistent non-specific low back pain. NICE clinical guideline 88*. 2009.

13. Department of Health. Physiotherapy Services Summary Information for 2004-2005, England http://www.hscic.gov.uk/searchcatalogue?productid=4306&kwd=K&gran=Government+Office+Regions&sort=Relevance&size=10&page=1#top (accessed 10.06.2013)

14. Salisbury C, Montgomery AA, Hollinghurst S et al. Effectiveness of PhysioDirect telephone assessment and advice services for patients with musculoskeletal problems: pragmatic randomised controlled trial. *Br Med J* 2013;346:f43.

15. Hodgson K. Kidderminster helpline proves a success. *Physiotherapy Frontline* 2001;7:22-23.

16. Taylor S, Ellis I, and Gallagher M. Patient satisfaction with a new physiotherapy telephone service for back pain patients. *Physiotherapy* 2002;88:645-657.

17. Clayson M and Woolvine M. Back pain direct clinic: a collaboration between general practitioners and physiotherapists. *Work Based Learning in Primary Care* 2004;2:38-43.

18. Turner D. An exploratory study of physiotherapy telephone assessment. *International Journal of Therapy and Rehabilitation* 2009;16:97-105.

19. Russell T, Truter P, Blumke R, Richardson B. The diagnostic accuracy of telerehabilitation for nonarticular lower-limb musculoskeletal disorders. *Telemed J & E-Health* 2010;16:585-594.

20. Patel S, Hossain FS, Colaco HB et al. The accuracy of primary care teams in diagnosing disorders of the shoulder. *J Eval Clin Pract* 2011;17:118-122.


For peer review only

21. Jette DU, Ardleigh K, Chandler K et al. Decision-making ability of physical therapists: physical therapy intervention or medical referral. *Phys Ther* 2006;86:1619-1629.

22. Salisbury C, Foster NE, Hopper C et al. A pragmatic randomised controlled trial of the effectiveness and cost-effectiveness of 'PhysioDirect' telephone assessment and advice services for physiotherapy. *Health Technology Assessment (Winchester, England)* /20;17:1-157.

23. National Institute for Health and Clinical Excellence. Guide to the Methods of Technology Appraisal http://www.nice.org.uk/media/B52/A7/TAMethodsGuideUpdatedJune2008.pdf (accessed 10.06.2013)

24. Brooks R, with the EuroQol Group. EuroQol: the current state of play. *Health Policy* 1996;53-72.

25. Dolan P, Gudex C, Kind P et al. *A social tariff for EuroQol: Results from a UK general population survey*. University of York, 1-9-1995.

26. Quality Metric. A community for measuring health outcomes using SF tools. http://www.sf-36.org/ (accessed 10.06.2013)

27. Paterson, C. Measuring outcomes in primary care: a patient generated measure, MYMOP, compared with the SF-36 survey. *Br Med J* 1996;312:1016-1020.

28. Pham T, Van Der Heijde D, Lassere M et al. Outcome variables for osteoarthritis clinical trials: The OMERACT-OARSI set of responder criteria. *J Rheumatol* 2003;30:1648-1654.

29. British National Formulary. British National Formulary. http://www.bnf.org/bnf (accessed 10.06.2013)

30. Curtis L. *Unit costs of health and social care 2009*. PSSRU, University of Kent, 2010.


For peer review only

31. Department of Health. NHS Reference costs 2009-2010. https://www.gov.uk/government/publications/nhs-reference-costs-2009-2010 (accessed 10.06.2013)

32. National Health Service. NHS Drug tariff. http://www.nhsbsa.nhs.uk/PrescriptionServices/924.aspx (accessed 10.06.2013)

33. The Automobile Association. The AA schedule of motoring costs. http://www.theaa.com/motoring_advice/running_costs/index.html (accessed 10.06.2013)

34. Office for National Statistics. Annual survey of hours and earnings 2009 http://www.ons.gov.uk/ons/rel/ashe/annual-survey-of-hours-and-earnings/2009-revised/index.html (accessed 10.06.2013)

35. Royal College of Nursing. Agenda for change – pay rates http://www.nhscareers.nhs.uk/working-in-the-nhs/pay-and-benefits/agenda-for-change-pay-rates (accessed 10.06.2013)

36. Scott A, Simoens S, Heaney D, et al. What does GP out of hours care cost? An analysis of different models of out of hours care in Scotland. *Scot Med J* 2004;49:61-66.

37. Manca A, Hawkins N, Sculpher M. Estimating mean QALYs in trial-based cost-effectiveness analysis: the importance of controlling for baseline utility. *Health Econ* 2005;14:487-496.

38. van Buuren S, Boshuizen HC, Knook D L. Multiple Imputation of missing blood pressure covariaties in survival analysis. *Stat Med* 1999;18:681-694:

39. StataCorp *Statistical Software: Release 11. College Station, TX: StataCorp LP.* 2009.

40. The CONSORT Group. CONSORT: the transparent reporting of trials http://www.consort-statement.org (accessed 10.06.2013)

For peer review only

41. Coast J, Hensher M, Mulligan J, et al. Conceptual and practical difficulties with the economic evaluation of health services developments. J *Health Serv Res Policy* 2000;5:42-48.

42. Leemrijse CJ, Swinkels IC, Veenhof C. Direct access to physical therapy in the Netherlands: results from the first year in community-based physical therapy. Phys Ther. 2008 88(8):936-46

43. Li LC, Townsend AF, Badley EM. Self-management interventions in the digital age: new approaches to support people with rheumatologic conditions. Best Pract Res Clin Rheumatol. 2012 26(3):321-33.

44. Henderson C, Knapp M, Ferbnandez J-L. Cost effectiveness of telehealth for patients with long term conditions (Whole Systems Demonstrator telehealth questionnaire study): nested economic evaluation in a pragmatic, cluster randomised controlled trial. Br Med J 2013;34

For peer review only

**Competing interestes**

We have read and understood the BMJ Group policy on declaration of interests and declare the following interests: none.

**Funding**

The research was funded by the Medical Research Council (MRC) and managed by the National Institute for Health Research (NIHR) on behalf of the MRC-NIHR partnership. The funder had no role in: the study design; the collection, analysis, or interpretation of data; the writing of the report; the decision to submit the paper for publication. The researchers are all independent of the funders. All researchers had access to all the data.

Nadine Foster is supported by a National Institute for Health Research NIHR Research Professorship NIHR-RP-011-015. The views expressed in this publication are those of the authors and not necessarily those of the NHS, the National Institute for Health Research or the Department of Health.

**Aurthors' contributions**

CS was principal investigator on the trial, SH took the lead on the economic evaluation. JC, NF, SG, JH, and AM were co-applicants and contributed to the conception and design. SH, JC and JB carried out the analysis with help from CS and AM, and all authors contributed to the interpretation of the data. SH and JC wrote the first draft of the paper with all authors contributing to subsequent revisions. All authors have seen and approved the final version.

**Acknowledgments**

We would like to thank the patients who contributed to this research; the physiotherapists, administrative staff, managers and commissioners who supported the set up and delivery of the trial in the four primary care trusts; participating general practices; the research support staff in Bristol and Keele; members of the Trial Steering Committee and Data Monitoring Committee; and Jill Gamlin and Nick Deane who developed the PhysioDirect assessment algorithms and software used in this trial.

**Data sharing**

The datasets will be made freely available on application to any bone-fide suitable qualified researcher upon submission of a protocol which provides a research question and research design which could be addressed by the data from this study, has been peer reviewed and received ethical and research governance approvals. Users of the data will also be subject to a written agreement which complies with the MRC guidance. Data will be made available for sharing only after the publication of the final project report and the main papers in peer reviewed journals.

For peer review only

**Table 1: Data sources and unit costs**

| | Unit cost (£) |
|---|---|
| **Primary and Community Care**[30] | |
| General Practitioner | |
| Surgery | 27.00 |
| Telephone consultation | 16.00 |
| Home visit | 91.00 |
| Practice nurse | |
| Surgery | 10.00 |
| Telephone consultation | 5.93 |
| Health Care Assistant/phlebotomist | |
| Surgery | 6.92 |
| District Nurse | |
| Home visit | 16.33 |
| **Out of Hours**[41] | |
| General practitioner | 23.50 |
| **Hospital care**[31] | |
| A&E | 103.00 |
| Outpatient visits | By Healthcare Resource Group, differentiated by first and follow-up |
| Inpatient stays | By Healthcare Resource Group |
| **Prescribed medication**[29] | per item, by name, strength and amount |
| **Mileage**[33] | 0.4612 |
| **Time off work**[34] | Median national wage by age and sex |

For peer review only

**Table 2: Number of physiotherapy consultations and mean duration, by type and group**

| | | Usual care (n=743) | PhysioDirect (n=1506) |
|---|---|---|---|
| **Face-to-face appointments** | mean (SD) number | 3.11(2.63) | 1.91(2.72) |
| | mean (SD) total duration (minutes) | 107.51(88.92) | 64.20(89.31) |
| **Telephone appointments** | mean (SD) number | 0.13(0.44) | 0.96 (0.63) |
| | mean (SD) total duration (minutes) | 4.21(14.64) | 27.37(19.92) |
| **Home visits** | mean (SD) number | 0.00 (0.06) | 0.00 (0.06) |
| | mean (SD) total duration (minutes) | 0.14(2.27) | 0.12(2.12) |
| **All physiotherapy contacts** | mean (SD) number | 3.25(2.70) | 2.87(2.94) |
| | mean (SD) total duration (minutes) | 111.86(90.50) | 91.70 (95.40) |

For peer review only

**Table 3: Health services resource use, by group. All available data.**

| | Mean (SD) number of consultations | | | |
|---|---|---|---|---|
| | **n** | **usual care** | **n** | **PhysioDirect** |
| GP consultations | 739 | 0.77(1.47) | 1484 | 0.87 (1.68) |
| Nurse consultations | 739 | 0.04 (0.22) | 1484 | 0.06 (0.32) |
| Other primary care consultations | 739 | 0.02(0.14) | 1484 | 0.02 (0.17) |
| **Total number of primary care contacts** | **739** | **0.83(1.56)** | **1484** | **0.96 (1.84)** |
| Number of prescriptions | 728 | 1.36 (2.73) | 1469 | 1.68 (3.72) |
| A&E (visits) | 467 | 0.02 (0.01) | 912 | 0.03 (0.01) |
| Out-patient (consultations) | 467 | 0.17 (0.83) | 910 | 0.35 (1.03) |
| In-patient stays (Finished Consultant Episodes) | 465 | 0.01 (0.10) | 910 | 0.01 (0.10) |

For peer review only


For peer review only - http://bmjopen.bmj.com/site/about/guidelines.xhtml

**Table 4: Patient and societal resource use, by group. All available data.**

| Number (%) reporting: | n | usual care | n | PhysioDirect |
|---|---|---|---|---|
| Expenditure on travel to physiotherapy | 462 | 242 (52.4) | 1232 | 308 (25.8) |
| Expenditure on travel to primary care | 669 | 116 (17.3) | 1337 | 237 (17.7) |
| Expenditure on over-the-counter medication | 506 | 256 (50.6) | 1028 | 512 (49.8) |
| Expenditure on prescriptions | 559 | 264 (47.2) | 1085 | 508 (46.8) |
| Expenditure on private therapy | 484 | 89 (18.4) | 934 | 167 (17.9) |
| Equipment purchase | 480 | 139 (29.0) | 939 | 233 (24.8) |
| Payments for extra domestic help | 459 | 35 (7.6) | 928 | 76 (8.2) |
| Loss of earnings | 598 | 30 (5.5) | 1209 | 64 (5.3) |
| Any time off to attend physiotherapy consultation | 692 | 218 (31.5) | 1416 | 380 (26.8) |
| Work has been affected because of condition | 477 | 141 (29.6) | 959 | 317 (33.1) |

For peer review only

**Table 5: Mean total cost per patient, by group and category. All available data[a].**

| | n | usual care mean (SD) cost | n | mean (SD) cost | Incremental difference (95% Confidence Interval) |
|---|---|---|---|---|---|
| **Physiotherapy services** | | | | | |
| Face-to-face appointments | 743 | £64.42 (£53.00) | 1506 | £38.76 (£53.92) | -£25.66 (-£30.37 to -£20.95) |
| Telephone appointments | 743 | £5.22 (£18.01) | 1506 | £35.17 (£26.34) | £29.94 (£27.84 to £32.05) |
| Home visits | 743 | £0.08 (£1.33) | 1506 | £0.08 (£1.46) | £0.00 (-£0.12 to £0.13) |
| **Total physiotherapy cost** | **743** | **£69.73 (£56.17)** | **1506** | **£74.01 (£63.97)** | **£4.28 (-£1.12 to £9.69)** |
| **Primary care services** | | | | | |
| GP consultations | 739 | £19.21 (£35.91) | 1484 | £21.69 (£41.66) | £2.48 (-£1.04 to £6.00) |
| Nurse consultations | 739 | £0.44 (£2.37) | 1484 | £0.61 (£3.17) | £0.16 (-£0.10 to £0.42) |
| Other primary care consultations | 739 | £0.03 (£0.57) | 1484 | £0.07 (£1.31) | £0.05 (-£0.05 to £0.15) |
| **Total primary care cost** | **739** | **£19.68 (£36.68)** | **1484** | **£22.37 (£42.83)** | **£2.69 (-£0.92 to £6.30)** |
| **Medication cost** | 728 | £11.04 (£51.61) | 1469 | £10.33 (£55.43) | -£0.72 (-£5.53 to £4.10) |
| **Hospital services** | | | | | |
| A&E | 467 | £1.99 (£17.12) | 912 | £3.17 (£20.84) | £1.18 (-£1.01 to £3.37) |
| Out-patient | 467 | £30.74 (£98.36) | 910 | £38.35 (£126.05) | £7.61 (-£5.50 to £20.72) |
| In-patient | 465 | £51.02 (£520.48) | 910 | £34.99 (£399.62) | -£16.03 (-£65.70 to £33.64) |
| **Total hospital cost** | **459** | **£83.04 (£561.68)** | **899** | **£77.00 (£446.24)** | **-£6.04 (-£60.99 to £48.91)** |
| **Personal expenditure** | | | | | |
| Cost of all calls to physiotherapy service | 743 | £0.97 (£0.99) | 1506 | £1.75 (£1.29) | £0.79 (£0.68 to £0.89) |
| Travel to physiotherapy | 462 | £6.11 (£11.48) | 1232 | £3.11 (£8.51) | -£3.01 (-£4.01 to -£2.00) |
| Travel for primary care | 669 | £0.65 (£2.93) | 1337 | £0.75 (£4.10) | £0.11 (-£0.24 to £0.45) |
| Over-the-counter medication | 490 | £7.67 (£14.09) | 987 | £8.61 (£22.38) | £0.94 (-£1.23 to £3.11) |

For peer review only

| | | | | | |
|---|---|---|---|---|---|
| Cost of prescriptions | 553 | £2.72 (£8.95) | 1076 | £2.67 (£8.33) | -£0.05 (-£0.93 to £0.82) |
| Private therapy | 475 | £21.98 (£70.34) | 915 | £39.34 (£296.94) | £17.36 (-£9.76 to £44.48) |
| Equipment purchase | 473 | £17.16 (£169.12) | 924 | £9.12 (£56.42) | -£8.04 (-£20.08 to £4.00) |
| Extra domestic help | 451 | £10.93 (£64.31) | 905 | £13.68 (£96.02) | £2.75 (-£7.07 to £12.56) |
| Cost associated with loss of earnings | 598 | £46.69 (409.72) | 1209 | £82.78 (£885.85) | £36.09 (-£38.63 to £110.81) |
| **Value of time off work** | | | | | |
| Time off work to attend physiotherapy | 598 | £12.90 (£38.99) | 1211 | £11.91 (£57.86) | £0.95 (-£3.81 to £5.70) |
| Time off work associated with the condition | 452 | £265.92 (£1350.82) | 884 | £226.61(£1139.84) | £111.31 (-£159.04 to £379.67) |

[a] Uses all available data, so denominators differ by category.

For peer review only

**Table 6: Cost-consequences. All available data[a].**

| | n (%) | usual care | n (%) | PhysioDirect | Incremental difference (95% Confidence Interval) |
|---|---|---|---|---|---|
| **Mean (SD) Cost** | | | | | |
| Total physiotherapy cost | 743 | £69.73 (£56.17) | 1506 | £74.01 (£63.97) | £4.28 (-£1.12 to £9.69) |
| Cost of NHS services including physiotherapy | 453 (61%) | £189.19 (£557.61) | 888 (59%) | £196.43 (£472.02) | £7.24 (-£49.68 to £64.10) |
| Total personal expenditure | 310 (42%) | £121.10 (£571.15) | 714 (47%) | £166.40 (£1040.27) | £45.30 (-£78.01 to £168.61) |
| Total value of all time off work[b] | 451 (61%) | £276.75 (£1355.00) | 883 (59%) | £240.74 (£1147.20) | -£36.01 (-£174.69 to £102.66) |
| **Consequences[c]** | | | | | **Difference/odds ratio (95% confidence interval)[d]** |
| SF36v2 PCS | 629 (85%) | 44.18 (10.84) | 1283 (85%) | 43.50 (10.94) | -0.01 (-0.80 to 0.79) |
| MYMOP[e] | 518 (70%) | 2.40 (1.38) | 1033 (69%) | 2.40 (1.43) | -0.02 (-0.16 to 0.11) |
| Global improvement score | 501 (67%) | 4.07 (1.40) | 1001 (66%) | 4.01 (1.44) | -0.08 (-0.23 to 0.08) |
| Response to treatment (OMERACT OARSI ) | 510 (69%) | 197 (38.6%) | 1029 (68%) | 430 (41.8%) | 1.14 (0.92 to 1.43) |
| Waiting time to first assessment and advice | 618 (83%) | 34 (20 to 55)[f] | 1281 (85%) | 7 (4 to 15)[f] | 0.32 (0.29 to 0.35)[g] |
| Patient overall satisfaction | 367 (49% | 79.7 (26.5) | 739 (49%) | 75.9 (28.3) | -3.8 (-7.3 to -0.3) |
| QALYs[h] | 454 (61%) | 0.322 (0.079) | 881 (58%) | 0.331 (0.082) | 0.009 (-0.000 to 0.018) |

[a] Uses all available data, so denominators differ by category
[b] Total of time off to attend physiotherapy and associated with the condition
[c] At 6 month follow-up time point
[d] Adjusted for outcome at baseline, gender, age, referral problem, PCT
[e] Lower score is better
[f] Median (IQR)
[g] Accelerated failure time analysis
[h] Adjusted for outcome at baseline

For peer review only



For peer review only - http://bmjopen.bmj.com/site/about/guidelines.xhtml

**Table 7: Cost-effectiveness analysis.** Includes cases with complete data on NHS costs and QALYs

| | n | usual care mean (SD) cost | n | PhysioDirect Mean (SD) cost | Incremental difference (95% CI) |
|---|---|---|---|---|---|
| **Cost of physiotherapy** | 432 | £78.77 (£57.08) | 840 | £86.75 (£65.47) | £7.98 (£0.69 to £15.27) |
| **Cost of NHS services other than physiotherapy** | 432 | £100.91 (£502.02) | 840 | £112.23 (£476.91) | £11.32 (-£45.08 to £67.72) |
| **Total cost including physiotherapy** | 432 | £179.68 (£504.73) | 840 | £198.98 (482.12) | £19.30 (-£37.60 to £76.19) |
| **QALYs** | 432 | 0.325 (0.077) | 840 | 0.332 (0.081) | 0.007 (-0.003 to 0.016) |
| **Incremental cost-effectiveness ratio (ICER)** | | | | | £2,889 |
| **Median Net Monetary Benefit (95% CI) based on bootstrapped results:** | | | | | |
| **λ=£20,000** | | | | | £117 (-£86 to £310) |
| probability of intervention being cost-effective | | | | | 0.88 |
| **λ=£30,000** | | | | | £184 (-£106 to £461) |
| probability of intervention being cost-effective | | | | | 0.90 |

For peer review only

**Table 8: Sensitivity Analysis**

| | n | Usual care mean (SD) | n | PhysioDirect mean (SD) | Incremental difference (95% confidence interval) |
|---|---|---|---|---|---|
| **(1) mimicking an efficient service** | | | | | |
| Cost of physiotherapy | 432 | £76.56 (£55.34) | 840 | £72.22 (£61.55) | -£4.34 (-£11.25 to £2.57) |
| Cost of NHS services | 432 | £100.91 (£502.02) | 840 | £112.23 (476.91) | £11.32 (-£45.08 to £67.72 |
| Total cost | 432 | £177.46 (£504.49) | 840 | £184.44 (£481.83) | £6.98 (-£49.89 to £63.85) |
| Incremental cost-effectiveness ratio (ICER) | | | | | £1,045 |
| Median Net Monetary Benefit (95% CI) based on bootstrapped results: | | | | | |
| λ=£20,000 | | | | | £127 (-£74 to £319) |
| λ=£30,000 | | | | | £193 (-£95 to £473) |
| **(2) Excluding hospital costs** | | | | | |
| Cost of physiotherapy | 448 | £78.49 (£57.14) | 869 | £86.84 (£65.25) | £8.35 (£1.21 to £15.50) |
| Cost of NHS services excluding secondary care | 448 | £33.75 (£76.46) | 869 | £33.49 (£63.76) | -£0.25 (-£8.05 to £7.54) |
| Total cost of NHS services including physiotherapy | 448 | £112.23 (£99.621) | 869 | £120.33 (£98.85) | £8.10 (-£3.21 to £19.41) |
| Incremental cost-effectiveness ratio (ICER) | | | | | £1,084 |
| Median Net Monetary Benefit (95% CI) based on bootstrapped results: | | | | | |
| λ=£20,000 | | | | | £142 (-£41 to £324) |
| λ=£30,000 | | | | | £217 (-£56 to £489) |
| **(3) Imputed data** | | | | | |
| Cost of physiotherapy | 743 | £69.73 (£56.17) | 1506 | £74.01 (£63.97) | £4.28 (-£1.12 to £9.69) |
| Cost of NHS services | 743 | £131.37 (£465.49) | 1506 | £131.51 (£384.36) | £0.17 (-£36.13 to £36.48) |
| Total cost of NHS services including physiotherapy | 743 | £201.09 (£467.51) | 1506 | £205.55 (£390.04) | £4.46 (-£32.22 to £41.14) |
| Quality Adjusted Life Years | 743 | 0.320 (0.003) | 1506 | 0.322 (0.002) | 0.002 (-0.006 to 0.009) |

For peer review only

| | | | | | |
|---|---|---|---|---|---|
| Incremental cost-effectiveness ratio (ICER) | | | | | £2,260 |
| Median Net Monetary Benefit (95% CI) based on bootstrapped results: | | | | | |
| λ=£20,000 | | | | | £34 (-£119 to £193) |
| λ=£30,000 | | | | | £52 (-£172 to £285) |
| **(4) Imputed data and 'efficient' service** | | | | | |
| Cost of physiotherapy | 743 | £67.61 (£54.19) | 1506 | £61.41 (£59.13 | -£6.20 (-£11.26 to -£1.14) |
| Cost of NHS services | 743 | £131.37 (£465.49) | 1506 | £131.54 (384.36) | £0.17 (-£36.13 to £36.48) |
| Total cost of NHS services including physiotherapy | 743 | £198.98 (£467.48) | 1506 | £192.95 (£389.52) | -£6.02 (-£42.68 to £30.63) |
| Quality Adjusted Life Years | 743 | 0.320 (0.003) | 1506 | 0.322(0.002) | 0.002 (-0.006 to 0.009) |
| Incremental cost-effectiveness ratio (ICER) | | | | | -£3,054 |
| Median Net Monetary Benefit (95% CI) based on bootstrapped results: | | | | | |
| λ=£20,000 | | | | | £47 (-£113 to £202) |
| λ=£30,000 | | | | | £67 (-£165 to £293) |

For peer review only

**Figure 1. Cost-effectiveness acceptability curve showing the probability that the intervention is cost-effective at different levels of willingness to pay for one Quality Adjusted Life Year**

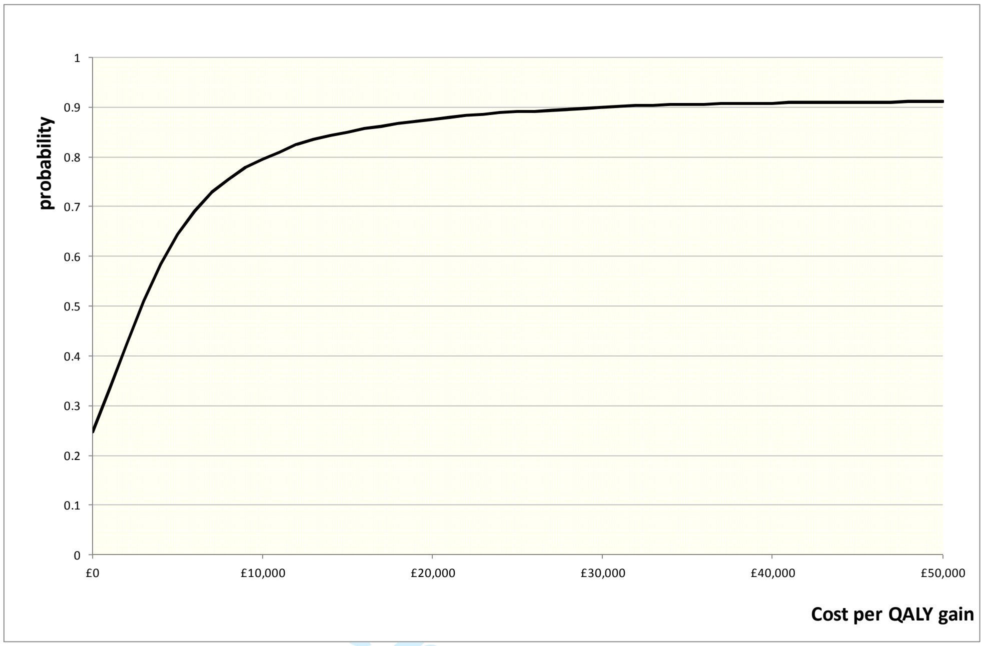


**Figure 2. Cost-effectiveness acceptability curve showing the probability that the intervention is cost-effective at different levels of willingness to pay for one Quality Adjusted Life Year: Sensitivity Analyses**

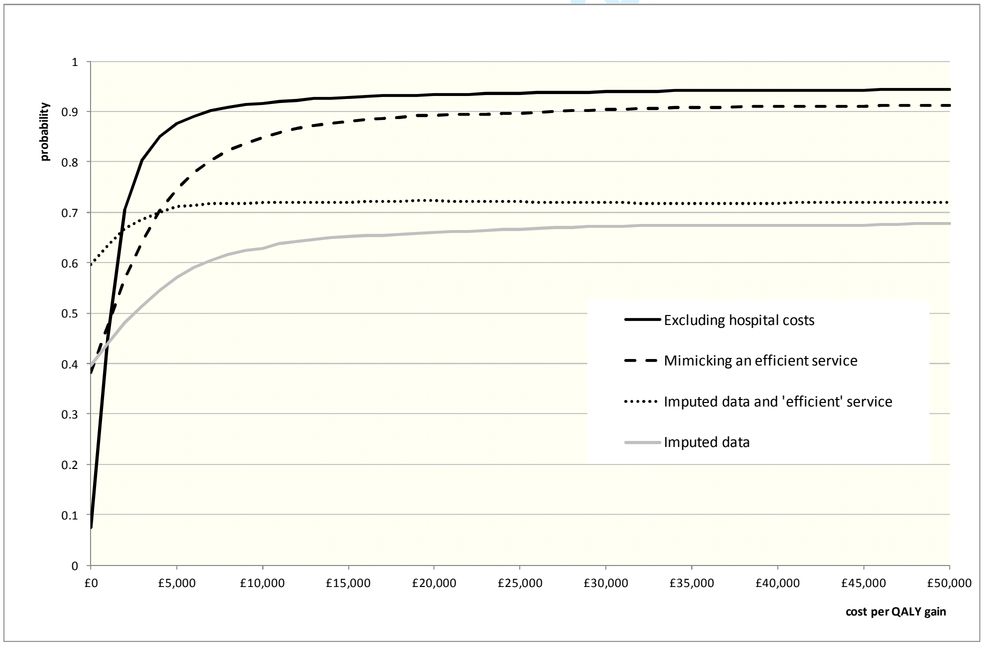

**CHEERS checklist—Items to include when reporting economic evaluations of health interventions**

| Section/item | Item No | Recommendation | Reported on page No/ line No |
|---|---|---|---|
| **Title and abstract** | | | |
| Title | 1 | Identify the study as an economic evaluation or use more specific terms such as "cost-effectiveness analysis", and describe the interventions compared. | 1/1-3 |
| Abstract | 2 | Provide a structured summary of objectives, perspective, setting, methods (including study design and inputs), results (including base case and uncertainty analyses), and conclusions. | P2-3 |
| **Introduction** | | | |
| Background and objectives | 3 | Provide an explicit statement of the broader context for the study. Present the study question and its relevance for health policy or practice decisions. | 5/1 – 6/20 |
| **Methods** | | | |
| Target population and subgroups | 4 | Describe characteristics of the base case population and subgroups analysed, including why they were chosen. | 7/12-16 |
| Setting and location | 5 | State relevant aspects of the system(s) in which the decision(s) need(s) to be made. | 7/12-16 |
| Study perspective | 6 | Describe the perspective of the study and relate this to the costs being evaluated. | 7/1-9 |
| Comparators | 7 | Describe the interventions or strategies being compared and state why they were chosen. | 7/20 - 8/2 |
| Time horizon | 8 | State the time horizon(s) over which costs and consequences are being evaluated and say why appropriate. | 6/25 - 7/1 |
| Discount rate | 9 | Report the choice of discount rate(s) used for costs and outcomes and say why appropriate. | 13/4-5 |
| Choice of health outcomes | 10 | Describe what outcomes were used as the measure(s) of benefit in the evaluation and their relevance for the type of analysis performed. | 8/5-13 |
| Measurement of effectiveness | 11a | *Single study-based estimates:* Describe fully the design features of the single effectiveness study and why the single study was a sufficient source of clinical effectiveness data. | 6/25-7/1 7/12-17 |
| | 11b | *Synthesis-based estimates:* Describe fully the methods used for identification of included studies and synthesis of clinical effectiveness data. | n/a |

For peer review only

| | | | |
|---|---|---|---|
| Measurement and valuation of preference based outcomes | 12 | If applicable, describe the population and methods used to elicit preferences for outcomes. | 8/5-6<br>12/6-7 |
| Estimating resources and costs | 13a | *Single study-based economic evaluation:* Describe approaches used to estimate resource use associated with the alternative interventions. Describe primary or secondary research methods for valuing each resource item in terms of its unit cost. Describe any adjustments made to approximate to opportunity costs. | 8/16 – 11/23 |
| | 13b | *Model-based economic evaluation:* Describe approaches and data sources used to estimate resource use associated with model health states. Describe primary or secondary research methods for valuing each resource item in terms of its unit cost. Describe any adjustments made to approximate to opportunity costs. | n/a |
| Currency, price date, and conversion | 14 | Report the dates of the estimated resource quantities and unit costs. Describe methods for adjusting estimated unit costs to the year of reported costs if necessary. Describe methods for converting costs into a common currency base and the exchange rate. | 11/25 |
| Choice of model | 15 | Describe and give reasons for the specific type of decision-analytical model used. Providing a figure to show model structure is strongly recommended. | n/a |
| Assumptions | 16 | Describe all structural or other assumptions underpinning the decision-analytical model. | n/a |
| Analytical methods | 17 | Describe all analytical methods supporting the evaluation. This could include methods for dealing with skewed, missing, or censored data; extrapolation methods; methods for pooling data; approaches to validate or make adjustments (such as half cycle corrections) to a model; and methods for handling population heterogeneity and uncertainty. | 12/7-10<br>12/23-13/2 |
| **Results** | | | |
| Study parameters | 18 | Report the values, ranges, references, and, if used, probability distributions for all parameters. Report reasons or sources for distributions used to represent uncertainty where appropriate. Providing a table to show the input values is strongly recommended. | n/a |
| Incremental costs and outcomes | 19 | For each intervention, report mean values for the main categories of estimated costs and outcomes of interest, as well as mean differences between the comparator groups. If applicable, report incremental cost-effectiveness ratios. | 15/1-23<br>Tables 5,6,7 |

For peer review only

| | | | |
|---|---|---|---|
| Characterising uncertainty | 20a | *Single study-based economic evaluation:* Describe the effects of sampling uncertainty for the estimated incremental cost and incremental effectiveness parameters, together with the impact of methodological assumptions (such as discount rate, study perspective). | 16/1-7<br>Table 7<br>Figure 1<br>16/13 – 17/16<br>Table 8<br>Figure 2 |
| | 20b | *Model-based economic evaluation:* Describe the effects on the results of uncertainty for all input parameters, and uncertainty related to the structure of the model and assumptions. | n/a |
| Characterising heterogeneity | 21 | If applicable, report differences in costs, outcomes, or cost-effectiveness that can be explained by variations between subgroups of patients with different baseline characteristics or other observed variability in effects that are not reducible by more information. | n/a |
| **Discussion** | | | |
| Study findings, limitations, generalisability, and current knowledge | 22 | Summarise key study findings and describe how they support the conclusions reached. Discuss limitations and the generalisability of the findings and how the findings fit with current knowledge. | 17/21 – 18/26 |
| **Other** | | | |
| Source of funding | 23 | Describe how the study was funded and the role of the funder in the identification, design, conduct, and reporting of the analysis. Describe other non-monetary sources of support. | 26/6-11 |
| Conflicts of interest | 24 | Describe any potential for conflict of interest of study contributors in accordance with journal policy. In the absence of a journal policy, we recommend authors comply with International Committee of Medical Journal Editors recommendations. | 26/1-3 |

**For consistency, the CHEERS statement checklist format is based on the format of the CONSORT statement checklist**

For peer review only

# A pragmatic randomised controlled trial of 'PhysioDirect' telephone assessment and advice services for patients with musculoskeletal problems: economic evaluation

| | |
|---|---|
| Journal: | *BMJ Open* |
| Manuscript ID: | bmjopen-2013-003406.R1 |
| Article Type: | Research |
| Date Submitted by the Author: | 23-Aug-2013 |
| Complete List of Authors: | Hollinghurst, Sandra; University of Bristol, Community Based Medicine<br>Coast, Joanna; University of Birmingham, Department of Health Economics<br>Busby, John; University of Bristol,<br>Bishop, Annette; Keele University,<br>Foster, Nadine; Keele University, Primary Care Musculoskeletal Research Centre<br>Franchini, Angelo; Imperial College, London,<br>Grove, Sean; Bristol Community Health,<br>Hall, Jeanette; Bristol Community Health,<br>Hopper, Cherida; University of Bristol,<br>Kaur, Surinder; University of Bristol,<br>Montgomery, Alan; University of Nottingham,<br>Salisbury, Chris; University of Bristol, Academic Unit of Primary Health Care |
| <b>Primary Subject Heading</b>: | Health economics |
| Secondary Subject Heading: | General practice / Family practice |
| Keywords: | economic evaluation, physiotherapy, telehealth, PRIMARY CARE, costs & cost analysis |

SCHOLARONE™ Manuscripts

# A pragmatic randomised controlled trial of 'PhysioDirect' telephone assessment and advice services for patients with musculoskeletal problems: economic evaluation

Sandra Hollinghurst, Joanna Coast, John Busby, Annette Bishop, Nadine E Foster, Angelo Franchini, Sean Grove, Jeanette Hall, Cherida Hopper, Surinder Kaur, Alan A Montgomery, Chris Salisbury

Sandra Hollinghurst, PhD. Senior Lecturer in Health Economics[1]
Joanna Coast, PhD. Professor of Health Economics[2]
John Busby, MSc. Research Assistant in Health Economics[1]
Annette Bishop, PhD. Research Physiotherapist[3]
Nadine E Foster, D.Phil. NIHR Professor of Musculoskeletal Health in Primary Care[3]
Angelo Franchini, MSc. Research Associate[4]
Sean Grove, MSc. Clinical Lead Physiotherapy[5]
Jeanette Hall, Grad Dip Phys. Operational lead for Outpatient Physiotherapy [5]
Cherida Hopper, PhD. Trial Manager[1]
Surinder Kaur, BSc. Research Associate[1]
Alan A Montgomery, PhD. Professor of Medical Statistics and Clinical Trials[6]
Chris Salisbury, MD. Professor of Primary Health Care [1]

[1]Centre for Academic Primary Care, School of Social and Community Medicine, University of Bristol, UK
[2]Health Economics Unit, School of Health & Population Sciences, University of Birmingham, UK
[3]Arthritis Research UK Primary Care Centre, Primary Care Sciences, Keele University, UK
[4] Imperial Clinical Trials Unit, School of Public Health Medicine, Imperial College, London, UK
[5]Musculoskeletal Outpatient Department, Bristol Community Health, UK
[6] Nottingham Clinical Trials Unit, Queen's Medical Centre, Nottingham, UK

**Correspondence:**
Sandra Hollinghurst, School of Social and Community Medicine, University of Bristol, Canynge Hall, 39 Whatley Road, Bristol BS8 2PS
s.p.hollinghurst@bristol.ac.uk
0117 331 3901

Key Words: economic evaluation; physiotherapy; telehealth; primary care; cost & cost analysis

For peer review only

# A pragmatic randomised controlled trial of 'PhysioDirect' telephone assessment and advice services for patients with musculoskeletal problems: economic evaluation


## ABSTRACT

### Objectives

To compare the cost-effectiveness of PhysioDirect with usual physiotherapy care for patients with musculoskeletal problems.

### Design

(i) Cost-consequences comparing cost to the NHS, to patients, and the value of lost productivity with a range of outcomes. (ii) Cost-utility analysis comparing cost to the NHS with Quality Adjusted Life Years (QALYs)

### Setting

Four physiotherapy services in England

### Participants

Adults (18+) referred by their general practitioner or self-referred for physiotherapy.

### Interventions

PhysioDirect involved telephone assessment and advice followed by face-to-face care if needed. Usual care patients were placed on a waiting list for face-to-face care.

### Primary and secondary outcomes

Primary clinical outcome: physical component summary from the SF-36v2 at six months. Also included in the cost-consequences: Measure Yourself Medical Outcomes Profile; a Global Improvement Score; response to treatment; patient satisfaction; waiting time.

Outcome for the cost-utility analysis: QALYs

### Results

2249 patients took part (1506 PhysioDirect; 743 usual care).


For peer review only

(i) Cost-consequences: there was no evidence of a difference between the two groups in the cost of physiotherapy, other NHS services, personal costs or value of time off work. Outcomes were also similar.

(ii) Cost-utility analysis based on complete cases (n=1,272). Total NHS costs, including the cost of physiotherapy were higher in the PhysioDirect group by £19.30 (95% CI: -£37.60 to £76.19) and there was a QALY gain of 0.007 (95% CI: -0.003 to 0.016). The incremental cost-effectiveness ratio was £2,889 and the net monetary benefit at λ=£20,000 was £117 (95% CI: -£86 to £310)

**Conclusions**

PhysioDirect may be a cost-effective alternative to usual physiotherapy care, though only with careful management of staff time. Physiotherapists providing the service must be more fully occupied than was possible under trial conditions: consideration should be given to the scale of operation, opening times of the service, and flexibility in the methods used to contact patients.

For peer review only

**Article Summary**

Article focus

- What is the cost of providing PhysioDirect telephone assessment and advice, followed by face-to-face care if needed, to adults with musculoskeletal pain problems, compared with usual physiotherapy care?
- What are the patient benefits of PhysioDirect compared with usual care?
- Is PhysioDirect a cost-effective alternative to usual care for primary care patients with musculoskeletal conditions?

Key messages

- There was very little difference between the two groups in terms of cost or outcomes
- PhysioDirect has potential to be a cost-effective alternative to usual care but requires careful management of staff resources

Strengths and limitations of this study

- Findings are based on a large sample of patients with a wide range of musculoskeletal problems based in diverse locations
- The study takes a broad perspective, including the health care provider, patients, and a valuation of lost productivity
- Physiotherapists were constrained by trial conditions and were underutilised

For peer review only

## INTRODUCTION

There is a trend to explore the use of new technology in the delivery of health care, particularly the use of telephone assessment and triage, as for example in NHS Direct.[1] These services aim to better manage patient demand, and research has shown that telephone based services can be safe, clinically accurate, cost-effective, acceptable to patients, and reduce the workload of clinicians,[2-5] although there have been some concerns about using telephone triage in patients presenting with acute health problems.[6]

Musculoskeletal pain problems are one of the most common causes of disability. Over a quarter of all patients registered in general practice will consult at least once for a musculoskeletal problem each year,[7,8] with musculoskeletal pain accounting for around 15% of all GP consultations.[9] This high prevalence of musculoskeletal problems[10] results in large direct and indirect healthcare costs: for example, low back pain alone has recently been estimated to cost the UK economy £15.84 billion a year.[11, 12] Most patients are managed with advice and analgesia but many are referred to physiotherapists, with 1.23 million new referrals each year from GPs and 4.4 million in total.[13] Ensuring timely access to physiotherapy has long been an issue within the UK NHS, with waiting times of more than four months in some areas. Patients may suffer unnecessary pain and disability, and there are high productivity losses: for example, back pain accounts for some 120 million days of certified absence from work each year.[14] Delay may also cause NHS inefficiencies on the one hand as some patients recover and do not attend their physiotherapy appointment when it finally arrives, whilst on the other, some patients continue to access more expensive forms of treatment whilst awaiting their appointment.

In response to these problems, physiotherapy services have drawn on the new service models and a range of 'PhysioDirect' services have been developed. These vary in format though they commonly involve a physiotherapist assessing a patient's musculoskeletal pain problem over the telephone,

For peer review only

sometimes supported by computerised assessment templates, offering tailored, self-management advice supplemented by written advice sent by post. Alternatively if the assessment findings suggest that face-to-face care is needed this is arranged, and patients who are initially managed by telephone advice can phone back for further advice and/or face-to-face treatment.[15] There is, however, limited evidence about the costs and benefits of this approach within physiotherapy. Local evaluations and non-randomised studies suggest that these services may be popular with patients[16-18] and diagnoses made by physiotherapists over the telephone are comparable to diagnoses reached in face-to-face assessments[19-21] although there is some concern that the experience of the physiotherapist providing the telephone assessment might be important.[19,22]

In particular there is no information about the costs or cost-effectiveness of PhysioDirect services, despite: (i) a major underlying rationale for their development being to generate greater efficiency in the use of resources; and (ii) a ready presumption that telephone-based services result in lower costs (by assuming that services better use physiotherapy time, use less costly telephone consultations and reduce rates of appointment non-attendance). Without such evidence, preferably generated alongside high quality primary evidence obtained using rigorous study designs, it remains unclear whether such services should be more widely implemented. This paper reports the results of an economic evaluation conducted alongside a randomised controlled trial powered to generate evidence on whether PhysioDirect services for primary care patients with musculoskeletal problems produce equivalent outcomes to usual face-to-face services.

## METHOD

### Study design

We conducted an economic evaluation alongside a randomised controlled trial to establish the cost-effectiveness of PhysioDirect compared with usual care based only on face-to-face treatment over a

period of 6 months. The trial and its clinical findings have been reported in full elsewhere.[15,23] The aim of the evaluation was to provide information about the long-run costs and benefits of the alternative methods of running a physiotherapy service for this patient group so with that in mind we excluded the initial set up costs associated with establishing the new telephone service, including the training undertaken by the practitioners.[24] As the nature of the intervention suggests there could be an impact on patients' costs, and as it is known that musculoskeletal conditions account for a considerable amount of time off work[14] we chose to use a cost-consequences approach, comparing cost from all three perspectives (health care provider, patients and carers, lost productivity) with a range of clinical outcomes.[25,26] However, the perspective of greatest interest to UK policy makers is the health and social care provider[23] so we also conducted a cost-utility analysis to compare cost to the National Health Service (NHS) with Quality-Adjusted Life-Years (QALYs).

**Setting and participants**

We recruited adults aged 18 and over from four community physiotherapy services in England – Bristol, Somerset, Stoke-on-Trent, and Cheshire – which provided diversity in terms of socio-economic status and a mix of urban and rural communities. All patients referred by their general practitioner (GP), or who referred themselves, for physiotherapy for a non-urgent musculoskeletal problem were invited to take part. Patients were randomised to PhysioDirect or usual care on a two to one basis to increase the chances of the PhysioDirect service being fully utilised.

**Interventions**

The intervention has been reported in detail elsewhere[15,25]. Patients randomised to the PhysioDirect service received an invitation to telephone a senior (band 6 or above) specially trained physiotherapist, who assessed their musculoskeletal problem with the aid of previously developed computerised templates.[23] These templates were provided by Huntingdonshire Primary Care Trust, which has been operating  a similar service since 2001. Patients were then sent appropriate advice

For peer review only

leaflets about self-management and exercises to try at home, and invited to phone again and/or make a face-to-face appointment if necessary. If the service was engaged when the patient called the call was answered by a receptionist who added the patient to a 'call-back' list and the physiotherapist would return the call when they were free. Patients randomised to usual care were put on the usual service waiting list for face-to-face assessment and treatment.

**Outcome measures**

We used the EQ-5D-3L[28], valued using the UK tariff,[29] to estimate QALYs gained for the cost-utility analysis. The primary outcome for the trial was the physical component summary (PCS) measure from the SF-36v2 questionnaire[30] and secondary clinical outcomes included: the Measure Yourself Medical Outcomes Profile (MYMOP);[31] a Global Improvement Score – a single question about overall improvement; a composite measure of response to treatment including pain, function and overall improvement (OMERACT OARSI);[32] patient satisfaction; and waiting time to first treatment advice from a physiotherapist. All outcomes (except the global improvement score and waiting time to first treatment advice) were measured at baseline, 6 weeks and 6 months and were obtained from a self completed questionnaire administered at these three time points.

**Resource use**

The analysis was based on costs related to the reason for which the patient was referred to the physiotherapy service. We identified relevant resources in discussion with participating physiotherapists and service managers. Direct costs to the health care provider included: cost of initial and follow-up physiotherapy consultations; primary and community consultations; hospital care; and prescribed medication. Patient and carer costs included: telephone calls to the PhysioDirect service; travel; over the counter medication; prescription costs; private therapy and purchase of equipment; extra domestic help; and loss of earnings. Lost productivity was estimated

For peer review only

separately in relation to time off work to attend physiotherapy appointments and time off because of the musculoskeletal condition itself.

Patient level data about all physiotherapy appointments and consultations were recorded either automatically by computer or by the physiotherapist treating the patient. For those in the intervention group, the PhysioDirect assessment software recorded which physiotherapist conducted each telephone call, and the duration of each call. In addition to the time logged on to the system physiotherapists had to carry out administrative activities following each telephone call, such as collating information to send to the patient by post. The time spent on these activities was estimated from information available at one site (Bristol) where manual recording of the entire encounter supplemented the electronic recording.

Physiotherapists assigned to the PhysioDirect service were required to be available throughout the time the service was 'open' but they were not usually fully engaged in dealing with PhysioDirect patients during these hours. We conducted an observational time and motion study at each of the four sites to determine how they occupied their non-PhysioDirect contact time in order to apportion costs appropriately. Time and motion data were collected at points in the study when the sites were expected to be fully operational, and across a mix of day, time of day, and location. The capital costs required to run a telephone service are potentially less than for a face-to-face service. Each site provided information about space and equipment required to run their telephone service and we used this to estimate an overall percentage reduction of capital costs for these compared with a standard face-to-face service.

Data about all face-to-face appointments were recorded routinely. These data included the length of appointment, the grade of the physiotherapist seen, and information about missed appointments.

For peer review only

Information about other NHS resource use was collected, where possible, from general practice records and supplemented by information gained directly from patients. General practice notes were scrutinised for patient level data on primary care consultations and prescribed medication. We included all consultations at which the musculoskeletal condition for which the patient was referred to physiotherapy was mentioned and these were recorded by type of consultation (for example, face-to-face, telephone, out of hours, home visit) and by type of professional seen (for example, GP, nurse). It was not feasible to distinguish between medication prescribed for the condition for which the patient was referred for physiotherapy and any other musculoskeletal problem so we included all medication of a potentially relevant type, defined using British National Formulary (BNF)[33] coding. These were: Analgesics (chapters 4.7.1 – 4.7.2); Non-steroidal anti-inflammatory drugs (10.1.1); Local corticosteroid injections (10.1.2.2); and Drugs for the relief of soft-tissue inflammation (10.3).

A questionnaire was administered to participants at 6 weeks and 6 months after randomisation to obtain resource use data not available elsewhere. The questionnaire was designed specifically for this study but was similar in content and structure to others used for the same purpose[34] Questions included information about hospital care related to the condition for which the patient was referred to physiotherapy: visits to Accident & Emergency, outpatient appointments, and inpatient stays. Information about personal expenditure relevant to the patient's musculoskeletal condition was also gained from the questionnaire at 6 weeks and 6 months. We asked about the cost of travel to physiotherapy and other health care appointments, expenditure on over the counter medication, prescription costs, use of private therapies and their cost, expenditure on equipment or devices, and extra help at home. In addition, participants were asked about any time off work, and the associated loss of earnings, because of their condition or to attend health care appointments relating to the condition including usual care physiotherapy and PhysioDirect.

**Valuation of resource use**

For peer review only

Table 1 gives the unit costs and sources used to value the health care resources. We used Curtis[35] to value primary and community health care and Department of Health reference costs[36] for all hospital-based care. The cost of prescribed medication was estimated from that published in the BNF,[33] adjusted to allow for the discount available to the NHS, and the professional fee and container allowance in accordance with the Drug Tariff for England.[37] Personal expenditure was reported directly by the participants, the exception being travel by car, which was reported as mileage and costed using the AA schedule of motoring costs.[38] Time off work was valued using the median gross weekly earnings by age and sex.[39]

*Table 1 here*

The cost of face-to-face physiotherapy consultations was estimated by adapting the methods of Curtis[35] to obtain a different unit cost for each band of staff at each site. National median pay rates, by band,[40] were adjusted to allow for National Insurance, superannuation, and overheads, as per Curtis, then further adjusted to allow for band and site specific non-contact time. Information about the proportion of time physiotherapists on each grade typically spend in direct contact with patients was provided by the four physiotherapy service managers. This provided us with a cost per hour for each band of staff at each site.

The unit cost of physiotherapists working in the PhysioDirect service was estimated in a similar way, but allowing for the reduced cost of capital and overheads; information from the site managers indicated this to be about 50%. To obtain a cost per hour of telephone contact we used information from the computerised records of the PhysioDirect service, which identified the proportion of time spent by physiotherapists actually dealing with PhysioDirect patients. We then combined this with data from the time and motion study, which identified activities undertaken during non-contact

For peer review only

time, for example, administration relating to face-to-face appointments or general administration, to give a cost per hour for each band of staff at each site.

All costs were valued in £ sterling at 2009 prices, adjusted for inflation where necessary.[35]

**Data analysis**

We investigated the amount of each resource used by patients in each group using frequencies, means and medians. Mean total cost per participant was derived by combining resource use with unit costs.

QALYs were derived from responses to the EQ-5D-3L at baseline, 6 weeks and 6 months using valuations from the UK general population.[29] These values, representing health-related quality of life on a scale between zero (death) and 1 (best imaginable health), were used to compute QALYs experienced over the 6 month period using the area under the curve approach and adjusting for any difference between the groups at baseline.[43]

A cost-consequences matrix was constructed using all available data. We compared costs from all three perspectives (health care provider, patients and carers, lost productivity) with the SF-36v2 PCS, MYMOP, Global Improvement Score, OMERACT OARSI, patient satisfaction, waiting time, and QALYs.

The cost-utility analysis was carried out using data on all patients for whom we had complete NHS cost and QALY data. An incremental cost-effectiveness ratio (ICER) was constructed, comparing the difference in mean total cost per patient with mean difference in QALYs, thus the lower the ICER, the greater the cost-effectiveness and the better the value for money. Uncertainty around the ICER was captured using the bootstrapping technique: 5000 replicates of the cost and QALY data were created by sampling from the original data, with replacement. The range and spread of the 5000 ICERs was

For peer review only

used to construct a cost-effectiveness acceptability curve to indicate the likelihood of the intervention being cost-effective. The net monetary benefit (NMB) of the intervention was estimated from the point estimate of the ICER for values of societal willingness-to-pay of £20,000 and £30,000 per QALY. If the NMB is positive at a given level of willingness to pay, the intervention is regarded as cost-effective. Confidence intervals around the NMB were formed from the bootstrapped estimates.

We used the multiple imputation by chained equation procedure to address the issue of missing cost and EQ-5D data.[44] This technique uses a regression model to estimate missing values from known values. In addition to cost and EQ-5D-3L variables the imputation model also included randomisation group, age, sex, and SF36v2 PCS. Stata v12[45] was used to generate five datasets using 10 switching procedures.

Discounting was not carried out because the analysis was restricted to costs and outcomes over a period of less than a year. All analyses were conducted using Microsoft Excel and Stata v12.[44]

**Sensitivity Analyses**

We addressed three areas of uncertainty using four one/two-way sensitivity analyses. First, we estimated the cost of running the PhysioDirect service if it was operating at full capacity. It is likely that this was not achieved during the trial because of: low demand due to exclusions and non-participation in the trial; inflexible staffing levels to ensure consistency throughout the trial period; and the 'one-way' system generally used, where physiotherapists waited for patients to call them but did not routinely contact patients themselves (notwithstanding some limited use of answer-machines). Data from the Bristol service, which continued to operate beyond the trial period and was then able to tailor staffing levels to demand, were used to estimate the cost of running a more 'efficient but feasible' PhysioDirect service once the trial had ended.


For peer review only - http://bmjopen.bmj.com/site/about/guidelines.xhtml

The second area of uncertainty addressed hospital costs. Patients in the trial were recruited from primary care and for these use of secondary care is infrequent but relatively expensive and this can have a disproportionate effect on mean total cost. We tested this by excluding hospital costs from the total.

The third area of uncertainty tested the effect of using imputed data rather than complete cases; the third sensitivity analysis used trial data with missing values imputed.

Finally, in a two-way sensitivity analysis, we re-estimated the results of the first, (mimicking an ‘efficient but feasible’ service) in this instance using the imputed dataset.

**RESULTS**

A total of 2,249 patients were recruited between July 2009 and December 2009, and followed up until June 2010, 1506 allocated to PhysioDirect and 743 to usual care. The mean age was 60, with slightly more females than males (60% vs 40%); they were overwhelmingly white (97%), just over half (60%) were employed and all but a few were referred for physiotherapy by their GP. Lower limb problems were the most prevalent (30%) reason for referral, 27% patients had a lumbar problem and 23% upper limb problems. Nearly all participants (2,223=99%) gave permission to access their GP notes to obtain data about primary care encounters and prescribed medication. 81% returned questionnaires at both 6 weeks and 6 months though not all participants completed all sections at both time points. We had complete NHS cost and QALY data for 840 (56%) PhysioDirect and 432 (58%) usual care participants.

**Resource use**

For peer review only



For peer review only - http://bmjopen.bmj.com/site/about/guidelines.xhtml

Table 2 gives information about the different types of physiotherapy consultations by patients in each group. Of the 1506 patients in the PhysioDirect group, 39% (586) were managed solely over the telephone and only 46% (695) had any face-to-face consultations. In total, patients in the usual care group had, on average, 0.38 (95% CI: 0.12 to 0.63) more consultations than those in the PhysioDirect group and the mean total duration of all consultations was 20 minutes longer (95% CI: 12 to 28).

*Table 2 here*

Tables 3 and 4 give information about NHS and personal resource use. Just over a third of patients (35%) had a GP consultation during the 6 months and 40% received a prescription for musculoskeletal pain-related medication. There was very little difference between the two groups in terms of health care use and the only notable difference in personal expenditure was travel to physiotherapy appointments.

*Tables 3 and 4 here*

**Costs and consequences**

Table 5 summarises the mean cost per patient, by group, for each category of cost. All available data are included giving variable denominators for each category. Comparing the two groups, there are small differences in cost in some categories but for most of these the confidence intervals indicate there is no evidence of a difference between the groups.

*Table 5 here*

Table 6 combines the results of the cost analysis with the full range of primary and secondary outcomes, including QALYs.

For peer review only

*Table 6 here*

Results are presented for all available data, with cost categories combined. Denominators vary within the table and they also differ from those in table 5 because sub-categories have been collapsed. There was no evidence of a difference in the primary clinical outcome (the SF36v2 PCS) between the groups, suggesting that PhysioDirect led to similar outcomes as usual physiotherapy care. Patients in the PhysioDirect group had their first assessment and telephone advice 27 days earlier than those in the usual care group, however patient satisfaction was slightly lower in those receiving PhysioDirect. QALYs were higher in the PhysioDirect group by 0.009, which equates to about 3.3 extra days of full health over a year.

**Cost-utility analysis**

The cost-utility analysis presented in Table 7 uses complete cases, that is, we include only those patients for whom we had complete NHS cost and QALY data: 432 (58%) from the usual care group and 840 (56%) from PhysioDirect. The small extra cost of caring for patients in the PhysioDirect group was compensated for by the extra QALY gain, giving an incremental cost-effectiveness ratio of £2,889. Values below £20,000 are regarded by NICE to indicate a cost-effective intervention[27] At this threshold level of willingness to pay for a QALY there is a positive net monetary benefit of £117 (95% CI -£96 to £310) and there is 0.88 probability that the intervention is cost-effective. This is illustrated in the cost-effectiveness acceptability curve in figure 1.

*Table 7 here*

*Figure 1 here*

**Sensitivity Analysis**

For peer review only

The results of the four sensitivity analyses are shown in table 8 and figure 2. Scenario (1) indicates the potential cost-effectiveness of a more efficient PhysioDirect service. During the trial PhysioDirect clinic opening hours, physiotherapists spent about 35% of their time on the phone or dealing with directly related administration; in Bristol after the trial, this was increased to 57%. Under this scenario, the cost per patient in the PhysioDirect group was £14.53 less than under trial conditions and £2.11 less per patient in the usual care group. The ICER is therefore lower, at £1,045, the NMB correspondingly higher at £127 ($\lambda$=£20,000). At low levels of $\lambda$, the probability of PhysioDirect being cost-effective under this scenario is higher than with the base case, though at $\lambda$=£20,000 it reaches a similar value (see figures 1 and 2).

*Table 8 here*

*Figure 2 here*

The effect of removing hospital costs from the analysis is shown in sensitivity analysis (2). Hospital costs accounted for 75% of all NHS costs yet only 19% (n=252) participants reported using any secondary care. Hospital use was evenly divided between the two groups so removing these from the analysis made very little difference to incremental analysis.

The effect of imputing missing NHS cost and QALY data is explored in sensitivity analysis (3). Using these data the cost of the interventions is lower but this is offset by higher NHS costs, giving a higher mean total cost in both groups, by £21.41 in the usual care group and £6.58 in the PhysioDirect group. QALYs using imputed data are lower, by 0.005 in the usual care group and by 0.010 in the PhysioDirect group. The net effect is a reduction of both incremental cost and incremental QALYs, giving an ICER of £2,260. Uncertainty around the ICER is reduced, as seen by the flatter CEAC in figure 2.

For peer review only

Sensitivity analysis (4) combines analyses (1) and (3) by using imputed cost data in the 'efficient service' scenario. In this case the results indicate that PhysioDirect is, on average, cheaper than usual care with a possible saving of £6.02 per patient, which gives a negative value for the ICER, indicating the intervention is superior in terms of both cost and outcome. The probability that the service is cost-effective at λ=£20,000 is 0.72.

## DISCUSSION

### Statement of principal findings

The results of this economic evaluation suggest that PhysioDirect services for patients with musculoskeletal problems require careful management if they are to be a cost-effective alternative to usual physiotherapy care. There was very little difference between the two groups in terms of either outcomes or costs, and the finding that PhysioDirect is cost-effective is based on evidence that it provides very slightly greater QALY benefits at very slightly greater cost.

Clearer cost savings were observed in the sensitivity analysis that replicated the post-trial service, once greater flexibility in working arrangements was implemented. Without the restrictions of a trial environment staffing was adjusted to meet the anticipated demand, a call-back service was employed which accommodated fluctuations in activity during each session, referrals added to the system were adjusted regularly to reflect actual staffing and the number of patients waiting for a call-back and a higher throughput of patients led to greater economies of scale. These changes ensured physiotherapists within the PhysioDirect service spent a higher proportion of their PhysioDirect clinic time on the telephone with patients. There was no evidence of a difference between PhysioDirect and usual care in cost to patients and their families, or to society through the costs of lost production.

For peer review only

**Strengths and weaknesses of the study**

The study has a number of strengths. It is the first study assessing the cost-effectiveness of a PhysioDirect service including a large sample of patients with a wide range of musculoskeletal problems based across a number of locations.[15] It uses a rigorous study design and conforms to CONSORT guidelines.[46] The follow up rate from participating patients was in line with other primary care trials,[47,48] a high proportion of the resource use data were collected from GP records, and there was collection of resource use information outside of the main health service perspective. The cost consequences analysis provides complete information on costs from different perspectives compared with a range of outcomes so although this approach is sometimes criticised for leaving the reader to evaluate the findings it does have the advantage of transparency. Furthermore, in this study we have also presented a cost-utility analysis that conforms to the recommendations of NICE. Nevertheless, there are also limitations. The practices recruited to the trial had a low proportion of ethnic minority patients, a slightly lower proportion of patients from deprived areas were judged to be eligible, and the proportion of eligible individuals consenting to participate in the trial was only 50%.[23] These factors limit the generalisability of the results though none of these selection effects was large. Further, a particular difficulty in conducting economic evaluation with new service developments is ensuring that they are fully utilised,[24] particularly when conducting analysis from a long-run perspective, as here. Although there was a clear run-in period prior to data collection for the trial (ranging from 4 to 12 weeks in each of the four sites), to ensure that services were operating as well as they could, and a 2:1 randomisation ratio in favour of PhysioDirect was used, there was still considerable underutilisation of the new service. This was ameliorated by including a more fully utilised service within the sensitivity analysis. It should also be noted that, because of the use of a long-run perspective in the analysis, set up costs, which may, in the short-term be important in a financially constrained service, are not included here. Finally, because the differences in both costs and effects are small, there is still some uncertainty around the findings.

For peer review only

**The meaning of the study and implications for policy makers**

If the aim of health services is to achieve maximum health gain from an investment in health care, then PhysioDirect has a high probability of being more cost-effective than usual physiotherapy care. This research also, however, suggests that it cannot be assumed that PhysioDirect will reduce costs, although it could potentially do so if PhysioDirect services are managed efficiently. Both the costs and cost-effectiveness of services will depend on the productivity of physiotherapist time. If physiotherapists are able to use most of their time dealing directly with patients during sessions when they are available on the telephone, then the service will be less costly. This is most likely to be achieved by operating a call-back service and/or by operating the service on a large scale to even out fluctuations in demand. Operating a system on  larger scale might also offer other economies of scale, particularly in terms of infrastructure. Further efficiencies may also be achieved if these services are, in the future, provided in conjunction with direct access for patients (rather than following referral from another health care professional), given that patients who self-refer are likely to contact the service with musculoskeletal problems of shorter duration,[49] and such patients may be particularly appropriate for the initial assessment and advice provided by a PhysioDirect service. More generally, the study has broader implications for telephone services, particularly around the implicit assumption that such services will inevitably be money saving. Here this assumption was found to be false, largely because the physiotherapists' time was underutilised during PhysioDirect clinic hours. Thus, for all such services, it will be important for policy makers to ensure that easy assumptions about the costs of these services are properly assessed in relation to factors such as how efficiently the service is run, and what proportion of patients are subsequently invited for face to face care following an initial telephone call.

**Unanswered questions and future research**

As services evolve, further research should explore the costs and benefits of PhysioDirect under different scenarios. These might include: comparing different skill levels of staff operating the


For peer review only - http://bmjopen.bmj.com/site/about/guidelines.xhtml

service; the inclusion of patient self-referrals in addition to GP referrals; the use, or otherwise, of computerised support in assessing the patient; the extension to internet services (possibly combined with cameras);[50] and the use of mobile 'smartphone' technology, for example in rapid assessment of musculoskeletal injuries. In particular, however, it will be important to assess the costs and benefits of services once they are more established and provided on a wider scale. The costs and benefits of telehealth more generally need further exploration in relation to their cost effectiveness particularly given the negative findings of the Whole Systems Demonstrator project evaluating telehealth support and treatment for patients with long term conditions.[51] It would be helpful to identify those characteristics that are likely to make services both more cost-effective and less costly.

For peer review only

## References

1. Munro J, Nicholl, J, O'Cathain A et al. Impact of NHS Direct on demand for immediate care: observational study. *Br Med J* 2000;321:150-153.

2. Lattimer V, Sassi F, George S et al. Cost analysis of nurse telephone consultation in out of hours primary care: evidence from a randomised controlled trial. *Br Med J* 2000;320:1053-1057.

3. Lattimer V, George S, Thompson F et al. Safety and effectiveness of nurse telephone consultation in out of hours primary care: randomised controlled trial. The South Wiltshire Out of Hours Project (SWOOP) Group. *Br Med J* 1998;317:1054-1059.

4. Pinnock H, Bawden R, Proctor S et al. Accessibility, acceptability, and effectiveness in primary care of routine telephone review of asthma: pragmatic, randomised controlled trial. *Br Med J* 2003;326:477-479.

5. Bunn F, Byrne G, Kendall S. Telephone consultation and triage: effects on health care use and patient satisfaction.*Cochrane Database of Systematic Reviews.(4):CD004180, 2004.* 2004;CD004180.

6. McKinstry B, Watson P, Pinnock H et al. Telephone consulting in primary care: a triangulated qualitative study of patients and providers. *Br J Gen Pract* 2009;59:e209-e218.

7. Picavet HS, Schouten JS. Musculoskeletal pain in the Netherlands: prevalences, consequences and risk groups, the DMC(3)-study. *Pain* 2003;102:167-178.

8. Jordan K, Ong BN, Croft P. Previous consultation and self reported health status as predictors of future demand for primary care. *J Epidemiol Commun Health* 2003;57:109-113.

9. McKormick A, Fleming D, and Charlton J *Morbidity statistics from general practice. Fourth national study 1991-1992 Serise MB5 no.3*. London: 1995.


For peer review only - http://bmjopen.bmj.com/site/about/guidelines.xhtml

10. Balage F, Mannion A, Pellise F, et al. Non-specific low back pain. *Lancet* 2012;379(9814):482-91.

11. Chuang L-H, Soares M, Tilbrook H, et al. A Pragmatic multicentred randomized controlled trial of yoga for chroonic low back pain: economic evaluation*. Spine* 2012;37(18):1593-1601.

12. National Institute for Health and Clinical Excellence *Low back pain: early management of persistent non-specific low back pain. NICE clinical guideline 88*. 2009.

13. Department of Health. Physiotherapy Services Summary Information for 2004-2005, England https://catalogue.ic.nhs.uk/publications/hospital/outpatients/nhs-phys-serv-summ-eng-2004-05/nhs-phys-serv-summ-eng-2004-05-rep.pdf (accessed 16.08.2013)

14. Maniadakis N, Gray A. The economic burden of back pain in the UK. *Pain* 2000;84:95-103.

15. Salisbury C, Montgomery AA, Hollinghurst S et al. Effectiveness of PhysioDirect telephone assessment and advice services for patients with musculoskeletal problems: pragmatic randomised controlled trial. *Br Med J* 2013;346:f43.

16. Hodgson K. Kidderminster helpline proves a success. *Physiotherapy Frontline* 2001;7:22-23.

17. Taylor S, Ellis I, and Gallagher M. Patient satisfaction with a new physiotherapy telephone service for back pain patients. *Physiotherapy* 2002;88:645-657.

18. Clayson M and Woolvine M. Back pain direct clinic: a collaboration between general practitioners and physiotherapists. *Work Based Learning in Primary Care* 2004;2:38-43.

19. Turner D. An exploratory study of physiotherapy telephone assessment. *International Journal of Therapy and Rehabilitation* 2009;16:97-105.

20. Russell T, Truter P, Blumke R, Richardson B. The diagnostic accuracy of telerehabilitation for nonarticular lower-limb musculoskeletal disorders. *Telemed J & E-Health* 2010;16:585-594.

For peer review only

21. Patel S, Hossain FS, Colaco HB et al. The accuracy of primary care teams in diagnosing disorders of the shoulder. *J Eval Clin Pract* 2011;17:118-122.

22. Jette DU, Ardleigh K, Chandler K et al. Decision-making ability of physical therapists: physical therapy intervention or medical referral. *Phys Ther* 2006;86:1619-1629.

23. Salisbury C, Foster NE, Hopper C et al. A pragmatic randomised controlled trial of the effectiveness and cost-effectiveness of 'PhysioDirect' telephone assessment and advice services for physiotherapy. *Health Technology Assessment (Winchester, England)* /20;17:1-157.

24. Coast J, Hensher M, Mulligan J, et al. Conceptual and practical difficulties with the economic evaluation of health services developments. J *Health Serv Res Policy* 2000;5:42-48.

25. Coast, J. Is economic evaluation in touch with society's health values? *Br Med J* 2004;(329):1233-1236.

26. Drummond M, Brown R, Fendrick M, et al. Use of Pharmacoeconomics Information—Report of the ISPOR Task Force on Use of Pharmacoeconomic/Health Economic Information in Health-Care Decision Making. *Value in Health* 2003;6 (4):407-416.

27. National Institute for Health and Clinical Excellence. Guide to the Methods of Technology Appraisal http://www.nice.org.uk/media/B52/A7/TAMethodsGuideUpdatedJune2008.pdf (accessed 16.08.2013)

28. Brooks R, with the EuroQol Group. EuroQol: the current state of play. *Health Policy* 1996;53-72.

29. Dolan P, Gudex C, Kind P et al. *A social tariff for EuroQol: Results from a UK general population survey*. University of York, 1-9-1995.

30. Quality Metric. A community for measuring health outcomes using SF tools. http://www.sf-36.org/ (accessed 16.08.2013)

For peer review only

31. Paterson, C. Measuring outcomes in primary care: a patient generated measure, MYMOP, compared with the SF-36 survey. *Br Med J* 1996;312:1016-1020.

32. Pham T, Van Der Heijde D, Lassere M et al. Outcome variables for osteoarthritis clinical trials: The OMERACT-OARSI set of responder criteria. *J Rheumatol* 2003;30:1648-1654.

33. British National Formulary. British National Formulary. http://www.bnf.org/bnf (accessed 16.08.2013)

34. Database of Instruments for Resource Use Measurement (DIRUM) http://www.dirum.org (accessed 16.08.2013)

35. Curtis L. *Unit costs of health and social care 2009*. PSSRU, University of Kent, 2010.

36. Department of Health. NHS Reference costs 2009-2010. https://www.gov.uk/government/publications/nhs-reference-costs-2009-2010 (accessed 16.08.2013)

37. National Health Service. NHS Drug tariff. http://www.nhsbsa.nhs.uk/PrescriptionServices/924.aspx (accessed 16.08.2013)

38. The Automobile Association. The AA schedule of motoring costs. http://www.theaa.com/motoring_advice/running_costs/index.html (accessed 16.08.2013)

39. Office for National Statistics. Annual survey of hours and earnings 2009 http://www.ons.gov.uk/ons/rel/ashe/annual-survey-of-hours-and-earnings/2009-revised/index.html (accessed 16.08.2013)

40. Royal College of Nursing. Agenda for change – pay rates http://www.nhscareers.nhs.uk/working-in-the-nhs/pay-and-benefits/agenda-for-change-pay-rates (accessed 16.08.2013)

For peer review only

41. Scott A, Simoens S, Heaney D, et al. What does GP out of hours care cost? An analysis of different models of out of hours care in Scotland. *Scot Med J* 2004;49:61-66.

42. O'Dowd A. Cost of out of hours care was 22% higher than predicted in England. *Br Med J* 2006;332:1113.4.

43. Manca A, Hawkins N, Sculpher M. Estimating mean QALYs in trial-based cost-effectiveness analysis: the importance of controlling for baseline utility. *Health Econ* 2005;14:487-496.

44. van Buuren S, Boshuizen HC, Knook D L. Multiple Imputation of missing blood pressure covariaties in survival analysis. *Stat Med* 1999;18:681-694:

45. StataCorp *Statistical Software: Release 11. College Station, TX: StataCorp LP.* 2009.

46. The CONSORT Group. CONSORT: the transparent reporting of trials http://www.consort-statement.org (accessed 16.08.2013)

47. Stamuli E, Bloor K, MacPherson H, et al. Cost-effectiveness of acupuncture for irritable bowel syndrome: findings from an economic evaluation conducted alongside a pragmatic randomised controlled trial in primary care. BMC Gastroenterology 2012; 12:149.

48. Hill JC, Whitehurst DG, Lewis M, et al. Comparison of stratified primary care management for low back pain with current best practice (STarT Back): a randomised controlled trial. Lancet 2011; 378 (9802): 1560-71.49. Leemrijse CJ, Swinkels IC, Veenhof C. Direct access to physical therapy in the Netherlands: results from the first year in community-based physical therapy. *Phys Ther*. 2008;88(8):936-46

50. Li LC, Townsend AF, Badley EM. Self-management interventions in the digital age: new approaches to support people with rheumatologic conditions. *Best Pract Res Clin Rheumatol*. 2012;26(3):321-33.



For peer review only - http://bmjopen.bmj.com/site/about/guidelines.xhtml

51. Henderson C, Knapp M, Ferbnandez J-L, et al. Cost effectiveness of telehealth for patients with long term conditions (Whole Systems Demonstrator telehealth questionnaire study): nested economic evaluation in a pragmatic, cluster randomised controlled trial. *Br Med J* 2013;34.j2065.

For peer review only

**Competing interestes**

We have read and understood the BMJ Group policy on declaration of interests and declare the following interests: none.

**Funding**

The research was funded by the Medical Research Council (MRC) and managed by the National Institute for Health Research (NIHR) on behalf of the MRC-NIHR partnership. The funder had no role in: the study design; the collection, analysis, or interpretation of data; the writing of the report; the decision to submit the paper for publication. The researchers are all independent of the funders. All researchers had access to all the data.

Nadine Foster is supported by a National Institute for Health Research NIHR Research Professorship NIHR-RP-011-015. The views expressed in this publication are those of the authors and not necessarily those of the NHS, the National Institute for Health Research or the Department of Health.

**Aurthors' contributions**

CS was principal investigator on the trial, SH took the lead on the economic evaluation. JC, NF, SG, JH, and AM were co-applicants and contributed to the conception and design. SH, JC and JB carried out the analysis with help from CS and AM, and all authors contributed to the interpretation of the data. SH and JC wrote the first draft of the paper with all authors contributing to subsequent revisions. All authors have seen and approved the final version.

**Acknowledgments**

We would like to dedicate this paper to the memory of Cherida Hopper, trial manager of the PhysioDirect study, who sadly died in June 2013.

We thank the patients who contributed to this research; the physiotherapists, administrative staff, managers and commissioners who supported the set up and delivery of the trial in the four primary care trusts; participating general practices; the research support staff in Bristol and Keele; members

For peer review only

of the Trial Steering Committee and Data Monitoring Committee; and Jill Gamlin and Nick Deane who developed the PhysioDirect assessment algorithms and software used in this trial.

For peer review only



For peer review only - http://bmjopen.bmj.com/site/about/guidelines.xhtml

**Table 1: Data sources and unit costs**

| | Unit cost (£) |
|---|---|
| **Primary and Community Care**[35] | |
| General Practitioner | |
| Surgery | 27.00 |
| Telephone consultation | 16.00 |
| Home visit | 91.00 |
| Practice nurse | |
| Surgery | 10.00 |
| Telephone consultation | 5.93 |
| Health Care Assistant/phlebotomist | |
| Surgery | 6.92 |
| District Nurse | |
| Home visit | 16.33 |
| **Out of Hours**[41,42] | |
| General practitioner | 23.50 |
| **Hospital care**[36] | |
| A&E | 103.00 |
| Outpatient visits | By Healthcare Resource Group, differentiated by first and follow-up |
| Inpatient stays | By Healthcare Resource Group |
| **Prescribed medication**[33] | per item, by name, strength and amount |
| **Mileage**[38] | 0.4612 |
| **Time off work**[39] | Median national wage by age and sex |

For peer review only

**Table 2: Number of physiotherapy consultations and mean duration, by type and group**

| | | Usual care (n=743) | PhysioDirect (n=1506) |
|---|---|---|---|
| **Face-to-face appointments** | mean (SD) number | 3.11(2.63) | 1.91(2.72) |
| | mean (SD) total duration (minutes) | 107.51(88.92) | 64.20(89.31) |
| **Telephone appointments** | mean (SD) number | 0.13(0.44) | 0.96 (0.63) |
| | mean (SD) total duration (minutes) | 4.21(14.64) | 27.37(19.92) |
| **Home visits** | mean (SD) number | 0.00 (0.06) | 0.00 (0.06) |
| | mean (SD) total duration (minutes) | 0.14(2.27) | 0.12(2.12) |
| **All physiotherapy contacts** | mean (SD) number | 3.25(2.70) | 2.87(2.94) |
| | mean (SD) total duration (minutes) | 111.86(90.50) | 91.70 (95.40) |

For peer review only

**Table 3: Health services resource use, by group. All available data.**

| | Mean (SD) number of consultations | | | |
|---|---|---|---|---|
| | **n** | **usual care** | **n** | **PhysioDirect** |
| GP consultations | 739 | 0.77(1.47) | 1484 | 0.87 (1.68) |
| Nurse consultations | 739 | 0.04 (0.22) | 1484 | 0.06 (0.32) |
| Other primary care consultations | 739 | 0.02(0.14) | 1484 | 0.02 (0.17) |
| **Total number of primary care contacts** | **739** | **0.83(1.56)** | **1484** | **0.96 (1.84)** |
| Number of prescriptions | 728 | 1.36 (2.73) | 1469 | 1.68 (3.72) |
| A&E (visits) | 467 | 0.02 (0.01) | 912 | 0.03 (0.01) |
| Out-patient (consultations) | 467 | 0.17 (0.83) | 910 | 0.35 (1.03) |
| In-patient stays (Finished Consultant Episodes) | 465 | 0.01 (0.10) | 910 | 0.01 (0.10) |

For peer review only

**Table 4: Patient and societal resource use, by group. All available data.**

| Number (%) reporting: | n | usual care | n | PhysioDirect |
|---|---|---|---|---|
| Expenditure on travel to physiotherapy | 462 | 242 (52.4) | 1232 | 308 (25.8) |
| Expenditure on travel to primary care | 669 | 116 (17.3) | 1337 | 237 (17.7) |
| Expenditure on over-the-counter medication | 506 | 256 (50.6) | 1028 | 512 (49.8) |
| Expenditure on prescriptions | 559 | 264 (47.2) | 1085 | 508 (46.8) |
| Expenditure on private therapy | 484 | 89 (18.4) | 934 | 167 (17.9) |
| Equipment purchase | 480 | 139 (29.0) | 939 | 233 (24.8) |
| Payments for extra domestic help | 459 | 35 (7.6) | 928 | 76 (8.2) |
| Loss of earnings | 598 | 30 (5.5) | 1209 | 64 (5.3) |
| Any time off to attend physiotherapy consultation | 692 | 218 (31.5) | 1416 | 380 (26.8) |
| Work has been affected because of condition | 477 | 141 (29.6) | 959 | 317 (33.1) |

For peer review only

**Table 5: Mean total cost per patient, by group and category. All available data[a].**

| | usual care | | PhysioDirect | | Incremental difference (95% Confidence Interval) |
|---|---|---|---|---|---|
| | n | mean (SD) cost | n | mean (SD) cost | |
| **Physiotherapy services** | | | | | |
| Face-to-face appointments | 743 | £64.42 (£53.00) | 1506 | £38.76 (£53.92) | -£25.66 (-£30.37 to -£20.95) |
| Telephone appointments | 743 | £5.22 (£18.01) | 1506 | £35.17 (£26.34) | £29.94 (£27.84 to £32.05) |
| Home visits | 743 | £0.08 (£1.33) | 1506 | £0.08 (£1.46) | £0.00 (-£0.12 to £0.13) |
| **Total physiotherapy cost** | **743** | **£69.73 (£56.17)** | **1506** | **£74.01 (£63.97)** | **£4.28 (-£1.12 to £9.69)** |
| **Primary care services** | | | | | |
| GP consultations | 739 | £19.21 (£35.91) | 1484 | £21.69 (£41.66) | £2.48 (-£1.04 to £6.00) |
| Nurse consultations | 739 | £0.44 (£2.37) | 1484 | £0.61 (£3.17) | £0.16 (-£0.10 to £0.42) |
| Other primary care consultations | 739 | £0.03 (£0.57) | 1484 | £0.07 (£1.31) | £0.05 (-£0.05 to £0.15) |
| **Total primary care cost** | **739** | **£19.68 (£36.68)** | **1484** | **£22.37 (£42.83)** | **£2.69 (-£0.92 to £6.30)** |
| **Medication cost** | 728 | £11.04 (£51.61) | 1469 | £10.33 (£55.43) | -£0.72 (-£5.53 to £4.10) |
| **Hospital services** | | | | | |
| A&E | 467 | £1.99 (£17.12) | 912 | £3.17 (£20.84) | £1.18 (-£1.01 to £3.37) |
| Out-patient | 467 | £30.74 (£98.36) | 910 | £38.35 (£126.05) | £7.61 (-£5.50 to £20.72) |
| In-patient | 465 | £51.02 (£520.48) | 910 | £34.99 (£399.62) | -£16.03 (-£65.70 to £33.64) |
| **Total hospital cost** | **459** | **£83.04 (£561.68)** | **899** | **£77.00 (£446.24)** | **-£6.04 (-£60.99 to £48.91)** |
| **Personal expenditure** | | | | | |
| Cost of all calls to physiotherapy service | 743 | £0.97 (£0.99) | 1506 | £1.75 (£1.29) | £0.79 (£0.68 to £0.89) |
| Travel to physiotherapy | 462 | £6.11 (£11.48) | 1232 | £3.11 (£8.51) | -£3.01 (-£4.01 to -£2.00) |
| Travel for primary care | 669 | £0.65 (£2.93) | 1337 | £0.75 (£4.10) | £0.11 (-£0.24 to £0.45) |
| Over-the-counter medication | 490 | £7.67 (£14.09) | 987 | £8.61 (£22.38) | £0.94 (-£1.23 to £3.11) |
| Cost of prescriptions | 553 | £2.72 (£8.95) | 1076 | £2.67 (£8.33) | -£0.05 (-£0.93 to £0.82) |

For peer review only

| | | | | | |
|---|---|---|---|---|---|
| Private therapy | 475 | £21.98 (£70.34) | 915 | £39.34 (£296.94) | £17.36 (-£9.76 to £44.48) |
| Equipment purchase | 473 | £17.16 (£169.12) | 924 | £9.12 (£56.42) | -£8.04 (-£20.08 to £4.00) |
| Extra domestic help | 451 | £10.93 (£64.31) | 905 | £13.68 (£96.02) | £2.75 (-£7.07 to £12.56) |
| Cost associated with loss of earnings | 598 | £46.69 (409.72) | 1209 | £82.78 (£885.85) | £36.09 (-£38.63 to £110.81) |
| **Value of time off work** | | | | | |
| Time off work to attend physiotherapy | 598 | £12.90 (£38.99) | 1211 | £11.91 (£57.86) | £0.95 (-£3.81 to £5.70) |
| Time off work associated with the condition | 452 | £265.92 (£1350.82) | 884 | £226.61(£1139.84) | £111.31 (-£159.04 to £379.67) |

[a] Uses all available data, so denominators differ by category.

For peer review only

**Table 6: Cost-consequences. All available data[a].**

| | n (%) | usual care | n (%) | PhysioDirect | Incremental difference (95% Confidence Interval) |
|---|---|---|---|---|---|
| **Mean (SD) Cost** | | | | | |
| Total physiotherapy cost | 743 | £69.73 (£56.17) | 1506 | £74.01 (£63.97) | £4.28 (-£1.12 to £9.69) |
| Cost of NHS services including physiotherapy | 453 (61%) | £189.19 (£557.61) | 888 (59%) | £196.43 (£472.02) | £7.24 (-£49.68 to £64.10) |
| Total personal expenditure | 310 (42%) | £121.10 (£575) | 714 (47%) | £166.40 (£1040.27) | £45.30 (-£78.01 to £168.61) |
| Total value of all time off work[b] | 451 (61%) | £276.75 (£1355.00) | 883 (59%) | £240.74 (£1147.20) | -£36.01 (-£174.69 to £102.66) |
| **Consequences[c]** | | | | | **Difference/odds ratio (95% confidence interval)[d]** |
| SF36v2 PCS | 629 (85%) | 44.18 (10.84) | 1283 (85%) | 43.50 (10.94) | -0.01 (-0.80 to 0.79) |
| MYMOP[e] | 518 (70%) | 2.40 (1.38) | 1033 (69%) | 2.40 (1.43) | -0.02 (-0.16 to 0.11) |
| Global improvement score | 501 (67%) | 4.07 (1.40) | 1001 (66%) | 4.01 (1.44) | -0.08 (-0.23 to 0.08) |
| Response to treatment (OMERACT OARSI ) | 510 (69%) | 197 (38.6%) | 1029 (68%) | 430 (41.8%) | 1.14 (0.92 to 1.43) |
| Waiting time to first assessment and advice | 618 (83%) | 34 (20 to 55)[f] | 1281 (85%) | 7 (4 to 15)[f] | 0.32 (0.29 to 0.35)[g] |
| Patient overall satisfaction | 367 (49% | 79.7 (26.5) | 739 (49%) | 75.9 (28.3) | -3.8 (-7.3 to -0.3) |
| QALYs[h] | 454 (61%) | 0.322 (0.079) | 881 (58%) | 0.331 (0.082) | 0.009 (-0.000 to 0.018) |

[a] Uses all available data, so denominators differ by category
[b] Total of time off to attend physiotherapy and associated with the condition
[c] At 6 month follow-up time point
[d] Adjusted for outcome at baseline, gender, age, referral problem, PCT
[e] Lower score is better
[f] Median (IQR)
[g] Accelerated failure time analysis
[h] Adjusted for outcome at baseline

For peer review only

**Table 7: Cost-effectiveness analysis.** Includes cases with complete data on NHS costs and QALYs

| | n | usual care mean (SD) cost | n | PhysioDirect Mean (SD) cost | Incremental difference (95% CI) |
|---|---|---|---|---|---|
| **Cost of physiotherapy** | 432 | £78.77 (£57.08) | 840 | £86.75 (£65.47) | £7.98 (£0.69 to £15.27) |
| **Cost of NHS services other than physiotherapy** | 432 | £100.91 (£502.02) | 840 | £112.23 (£476.91) | £11.32 (-£45.08 to £67.72) |
| **Total cost including physiotherapy** | 432 | £179.68 (£504.73) | 840 | £198.98 (482.12) | £19.30 (-£37.60 to £76.19) |
| **QALYs** | 432 | 0.325 (0.077) | 840 | 0.332 (0.081) | 0.007 (-0.003 to 0.016) |
| **Incremental cost-effectiveness ratio (ICER)** | | | | | £2,889 |
| **Median Net Monetary Benefit (95% CI) based on bootstrapped results:** | | | | | |
| **λ=£20,000** | | | | | £117 (-£86 to £310) |
| probability of intervention being cost-effective | | | | | 0.88 |
| **λ=£30,000** | | | | | £184 (-£106 to £461) |
| probability of intervention being cost-effective | | | | | 0.90 |

For peer review only

**Table 8: Sensitivity Analysis**

| | Usual care | | PhysioDirect | | Incremental difference |
|---|---|---|---|---|---|
| | n | mean (SD) | n | mean (SD) | (95% confidence interval) |
| **(1) mimicking an efficient service** | | | | | |
| Cost of physiotherapy | 432 | £76.56 (£55.34) | 840 | £72.22 (£61.55) | -£4.34 (-£11.25 to £2.57) |
| Cost of NHS services | 432 | £100.91 (£502.02) | 840 | £112.23 (476.91) | £11.32 (-£45.08 to £67.72 |
| Total cost | 432 | £177.46 (£504.49) | 840 | £184.44 (£481.83) | £6.98 (-£49.89 to £63.85) |
| Incremental cost-effectiveness ratio (ICER) | | | | | £1,045 |
| Median Net Monetary Benefit (95% CI) based on bootstrapped results: | | | | | |
| λ=£20,000 | | | | | £127 (-£74 to £319) |
| λ=£30,000 | | | | | £193 (-£95 to £473) |
| **(2) Excluding hospital costs** | | | | | |
| Cost of physiotherapy | 448 | £78.49 (£57.14) | 869 | £86.84 (£65.25) | £8.35 (£1.21 to £15.50) |
| Cost of NHS services excluding secondary care | 448 | £33.75 (£76.46) | 869 | £33.49 (£63.76) | -£0.25 (-£8.05 to £7.54) |
| Total cost of NHS services including physiotherapy | 448 | £112.23 (£99.621) | 869 | £120.33 (£98.85) | £8.10 (-£3.21 to £19.41) |
| Incremental cost-effectiveness ratio (ICER) | | | | | £1,084 |
| Median Net Monetary Benefit (95% CI) based on bootstrapped results: | | | | | |
| λ=£20,000 | | | | | £142 (-£41 to £324) |
| λ=£30,000 | | | | | £217 (-£56 to £489) |
| **(3) Imputed data** | | | | | |
| Cost of physiotherapy | 743 | £69.73 (£56.17) | 1506 | £74.01 (£63.97) | £4.28 (-£1.12 to £9.69) |
| Cost of NHS services | 743 | £131.37 (£465.49) | 1506 | £131.51 (£384.36) | £0.17 (-£36.13 to £36.48) |
| Total cost of NHS services including physiotherapy | 743 | £201.09 (£467.51) | 1506 | £205.55 (£390.04) | £4.46 (-£32.22 to £41.14) |
| Quality Adjusted Life Years | 743 | 0.320 (0.003) | 1506 | 0.322 (0.002) | 0.002 (-0.006 to 0.009) |
| Incremental cost-effectiveness ratio (ICER) | | | | | £2,260 |
| Median Net Monetary Benefit (95% CI) based on bootstrapped results: | | | | | |
| λ=£20,000 | | | | | £34 (-£119 to £193) |
| λ=£30,000 | | | | | £52 (-£172 to £285) |
| **(4) Imputed data and 'efficient' service** | | | | | |
| Cost of physiotherapy | 743 | £67.61 (£54.19) | 1506 | £61.41 (£59.13 | -£6.20 (-£11.26 to -£1.14) |
| Cost of NHS services | 743 | £131.37 (£465.49) | 1506 | £131.54 (384.36) | £0.17 (-£36.13 to £36.48) |
| Total cost of NHS services including physiotherapy | 743 | £198.98 (£467.48) | 1506 | £192.95 (£389.52) | -£6.02 (-£42.68 to £30.63) |
| Quality Adjusted Life Years | 743 | 0.320 (0.003) | 1506 | 0.322(0.002) | 0.002 (-0.006 to 0.009) |
| Incremental cost-effectiveness ratio (ICER) | | | | | -£3,054 |
| Median Net Monetary Benefit (95% CI) based on bootstrapped results: | | | | | |
| λ=£20,000 | | | | | £47 (-£113 to £202) |
| λ=£30,000 | | | | | £67 (-£165 to £293) |

For peer review only

**Figure 1. Cost-effectiveness acceptability curve showing the probability that the intervention is cost-effective at different levels of willingness to pay for one Quality Adjusted Life Year**

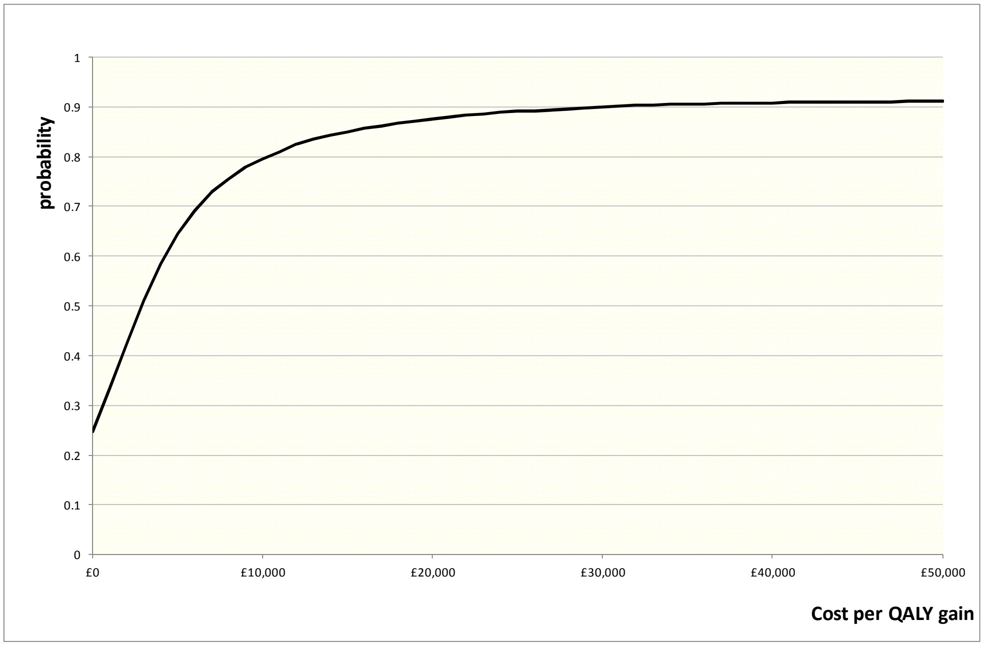


**Figure 2. Cost-effectiveness acceptability curve showing the probability that the intervention is cost-effective at different levels of willingness to pay for one Quality Adjusted Life Year: Sensitivity Analyses**

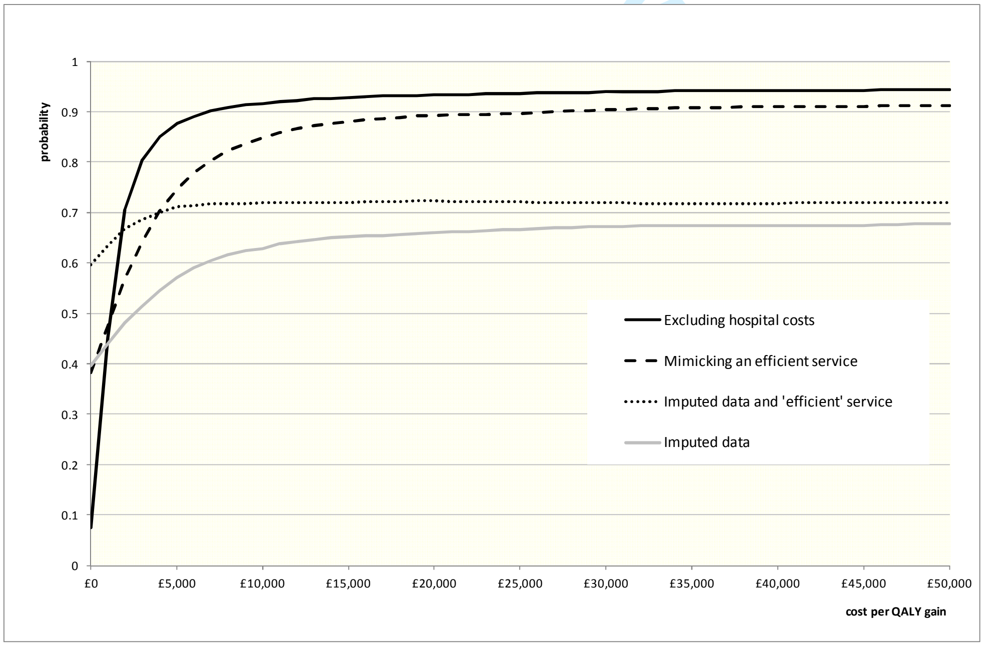

# A pragmatic randomised controlled trial of 'PhysioDirect' telephone assessment and advice services for patients with musculoskeletal problems: economic evaluation

Sandra Hollinghurst, Joanna Coast, John Busby, Annette Bishop, Nadine E Foster, Angelo Franchini, Sean Grove, Jeanette Hall, Cherida Hopper, Surinder Kaur, Alan A Montgomery, Chris Salisbury

Sandra Hollinghurst, PhD. Senior Lecturer in Health Economics[1]
Joanna Coast, PhD. Professor of Health Economics[2]
John Busby, MSc. Research Assistant in Health Economics[1]
Annette Bishop, PhD. Research Physiotherapist[3]
Nadine E Foster, D.Phil. NIHR Professor of Musculoskeletal Health in Primary Care[3]
Angelo Franchini, MSc. Research Associate[4]
Sean Grove, MSc. Clinical Lead Physiotherapy[5]
Jeanette Hall, Grad Dip Phys. Operational lead for Outpatient Physiotherapy [5]
Cherida Hopper, PhD. Trial Manager[1]
Surinder Kaur, BSc. Research Associate[1]
Alan A Montgomery, PhD. Professor of Medical Statistics and Clinical Trials[6]
Chris Salisbury, MD. Professor of Primary Health Care [1]

[1]Centre for Academic Primary Care, School of Social and Community Medicine, University of Bristol, UK
[2]Health Economics Unit, School of Health & Population Sciences, University of Birmingham, UK
[3]Arthritis Research UK Primary Care Centre, Primary Care Sciences, Keele University, UK
[4] Imperial Clinical Trials Unit, School of Public Health Medicine, Imperial College, London, UK
[5]Musculoskeletal Outpatient Department, Bristol Community Health, UK
[6] Nottingham Clinical Trials Unit, Queen's Medical Centre, Nottingham, UK

**Correspondence:**
Sandra Hollinghurst, School of Social and Community Medicine, University of Bristol, Canynge Hall, 39 Whatley Road, Bristol BS8 2PS
s.p.hollinghurst@bristol.ac.uk
0117 331 3901

Key Words: economic evaluation; physiotherapy; telehealth; primary care; cost & cost analysis



For peer review only - http://bmjopen.bmj.com/site/about/guidelines.xhtml

# A pragmatic randomised controlled trial of 'PhysioDirect' telephone assessment and advice services for patients with musculoskeletal problems: economic evaluation

## ABSTRACT


### Objectives

To compare the cost-effectiveness of PhysioDirect with usual physiotherapy care for patients with musculoskeletal problems.

### Design

(i) Cost-consequences comparing cost to the NHS, to patients, and the value of lost productivity with a range of outcomes. (ii) Cost-utility analysis comparing cost to the NHS with Quality Adjusted Life Years (QALYs)

### Setting

Four physiotherapy services in England

### Participants

Adults (18+) referred by their general practitioner or self-referred for physiotherapy.

### Interventions

PhysioDirect involved telephone assessment and advice followed by face-to-face care if needed. Usual care patients were placed on a waiting list for face-to-face care.

### Primary and secondary outcome ~~measure~~s

Primary clinical outcome: physical component summary ~~measure~~ from the SF-36v2 at six months.

Also included in the cost-consequences~~Secondary outcomes included~~: Measure Yourself Medical Outcomes Profile; a Global Improvement Score; response to treatment; patient satisfaction; waiting time.

Outcome for the cost-utility analysis: QALYs

### Results

2249 patients took part (1506 PhysioDirect; 743 usual care).


For peer review only



For peer review only - http://bmjopen.bmj.com/site/about/guidelines.xhtml

(i) Cost-consequences: there was no evidence of a difference between the two groups in the cost of physiotherapy, other NHS services, personal costs or value of time off work. Outcomes were also similar.

(ii) Cost-utility analysis based on complete cases (n=1,272). Total NHS costs, including the cost of physiotherapy were higher in the PhysioDirect group by £19.30 (95% CI: -£37.60 to £76.19) and there was a QALY gain of 0.007 (95% CI: -0.003 to 0.016). The incremental cost-effectiveness ratio was £2,889 and the net monetary benefit at λ=£20,000 was £117 (95% CI: -£86 to £310)

**Conclusions**

PhysioDirect may be a cost-effective alternative to usual physiotherapy care, though only with careful management of staff time. Physiotherapists providing the service must be more fully occupied than was possible under trial conditions: consideration should be given to the scale of operation, opening times of the service, and flexibility in the methods used to contact patients.



For peer review only

**Article Summary**

Article focus

- What is the cost of providing PhysioDirect telephone assessment and advice, followed by face-to-face care if needed, to adults with musculoskeletal pain problems, compared with usual physiotherapy care?
- What are the patient benefits of PhysioDirect compared with usual care?
- Is PhysioDirect a cost-effective alternative to usual care for primary care patients with musculoskeletal conditions?

Key messages

- There was very little difference between the two groups in terms of cost or outcomes
- PhysioDirect has potential to be a cost-effective alternative to usual care but requires careful management of staff resources

Strengths and limitations of this study

- Findings are based on a large sample of patients with a wide range of musculoskeletal problems based in diverse locations
- The study takes a broad perspective, including the health care provider, patients, and a valuation of lost productivity
- Physiotherapists were constrained by trial conditions and were underutilised



For peer review only - http://bmjopen.bmj.com/site/about/guidelines.xhtml

## INTRODUCTION

There is a trend to explore the use of new technology in the delivery of health care, particularly the use of telephone assessment and triage, as for example in NHS Direct.[1] These services aim to better manage patient demand, and research has shown that telephone based services can be safe, clinically accurate, cost-effective, acceptable to patients, and reduce the workload of clinicians,[2-5] although there have been some concerns about using telephone triage in patients presenting with acute health problems.[6]

Musculoskeletal pain problems are one of the most common causes of disability. Over a quarter of all patients registered in general practice will consult at least once for a musculoskeletal problem each year,[7,8] with musculoskeletal pain accounting for around 15% of all GP consultations.[9] This high prevalence of musculoskeletal problems[10] results in large direct and indirect healthcare costs: ;[10] estimated for for example, low back pain alone has recently been estimated to cost the UK economy £15.84 billion a yearat £10,668 million for the UK in 1998[11] and rising substantially since then.[11, 12]

Formatted: Superscript

Formatted: Superscript

Most patients are managed with advice and analgesia but many are referred to physiotherapists, with 1.23 million new referrals each year from GPs and 4.4 million in total.[13] Ensuring timely access to physiotherapy has long been an issue within the UK NHS, with waiting times of more than four months in some areas. Patients may suffer unnecessary pain and disability, and there are high productivity losses: for example, back pain accounts for some 120 million days of certified absence from work each year.[1411] Delay may also cause NHS inefficiencies on the one hand as some patients recover and do not attend their physiotherapy appointment when it finally arrives, whilst on the other, some patients continue to access more expensive forms of treatment whilst awaiting their appointment.

In response to these problems, physiotherapy services have drawn on the new service models and a range of 'PhysioDirect' services have been developed. These vary in format though they commonly



For peer review only - http://bmjopen.bmj.com/site/about/guidelines.xhtml

For peer review only

involve a physiotherapist assessing a patient's musculoskeletal pain problem over the telephone, sometimes supported by computerised assessment templates, offering tailored, self-management advice supplemented by written advice sent by post. Alternatively if the assessment findings suggest that face-to-face care is needed this is arranged, and patients who are initially managed by telephone advice can phone back for further advice and/or face-to-face treatment.[154] There is, however, limited evidence about the costs and benefits of this approach within physiotherapy. Local evaluations and non-randomised studies suggest that these services may be popular with patients[165-187] and diagnoses made by physiotherapists over the telephone are comparable to diagnoses reached in face-to-face assessments[198-210] although there is some concern that the experience of the physiotherapist providing the telephone assessment might be important.[198,221]

In particular there is no information about the costs or cost-effectiveness of PhysioDirect services, despite: (i) a major underlying rationale for their development being to generate greater efficiency in the use of resources; and (ii) a ready presumption that telephone-based services result in lower costs (by assuming that services better use physiotherapy time, use less costly telephone consultations and reduce rates of appointment non-attendance). Without such evidence, preferably generated alongside high quality primary evidence obtained using rigorous study designs, it remains unclear whether such services should be more widely implemented. This paper reports the results of an economic evaluation conducted alongside a randomised controlled trial powered to generate evidence on whether PhysioDirect services for primary care patients with musculoskeletal problems produce equivalent outcomes to usual face-to-face services.

## METHOD

**Study design**



For peer review only

For peer review only - http://bmjopen.bmj.com/site/about/guidelines.xhtml

We conducted an economic evaluation alongside a randomised controlled trial to establish the cost-effectiveness of PhysioDirect compared with usual care based only on face-to-face treatment over a period of 6 months. The trial and its clinical findings have been reported in full elsewhere.[154,2332] The aim of the evaluation was to provide information about the long-run costs and benefits of the alternative methods of running a physiotherapy service for this patient group so with that in mind we excluded the initial set up costs associated with establishing the new telephone service, including the training undertaken by the practitioners.[24] As the nature of the intervention suggests there could be an impact on patients' costs, and as it is known that musculoskeletal conditions account for a considerable amount of time off work[14] we chose to use a cost-consequences approach, comparing cost from all three perspectives (health care provider, patients and carers, lost productivity) with a range of clinical outcomes.[25,26] However, the perspective of greatest interest to UK policy makers is the health and social care provider[23] so we also conducted a cost-utility analysis to compare cost to the National Health Service (NHS) with Quality-Adjusted Life-Years (QALYs). However, the nature of the intervention suggests there could be an impact on patients' costs, and as it is known that musculoskeletal conditions account for a considerable amount of time off work[10,11] we included these perspectives in the analysis. We used a cost-consequences approach to compare cost from all three perspectives (health care provider, patients and carers, lost productivity) with a range of clinical outcomes and used a cost-utility analysis to compare cost to the National Health Service (NHS) with Quality-Adjusted Life-Years (QALYs).

The primary perspective for the economic evaluation was the health and social care provider, as that is of greatest interest to UK policy makers.[23] However, the nature of the intervention suggests there could be an impact on patients' costs, and as it is known that musculoskeletal conditions account for a considerable amount of time off work[10,11] we included these perspectives in the analysis. We used a cost-consequences approach to compare cost from all three perspectives (health care provider, patients and carers, lost productivity) with a range of clinical outcomes and used a cost-utility analysis

Formatted: Superscript, Not Highlight

Formatted: Superscript

Formatted: Superscript



For peer review only - http://bmjopen.bmj.com/site/about/guidelines.xhtml

~~analysis to compare cost to the National Health Service (NHS) with Quality-Adjusted Life-Years (QALYs).~~

**Setting and participants**

We recruited adults aged 18 and over from four community physiotherapy services in England – Bristol, Somerset, Stoke-on-Trent, and Cheshire – which provided diversity in terms of socio-economic status and a mix of urban and rural communities. All patients referred by their general practitioner (GP), or who referred themselves, for physiotherapy for a non-urgent musculoskeletal problem were invited to take part. Patients were randomised to PhysioDirect or usual care on a two to one basis to increase the chances of the PhysioDirect service being fully utilised.

**Interventions**

The intervention has been reported in detail elsewhere[154,252]. Patients randomised to the PhysioDirect service received an invitation to telephone a senior (band 6 or above) specially trained physiotherapist, who assessed their musculoskeletal problem with the aid of previously developed computerised templates.[232] These templates were provided by Huntingdonshire Primary Care Trust, which has been operating  a similar service since 2001. Patients were then sent appropriate advice leaflets about self-management and exercises to try at home, and invited to phone again and/or make a face-to-face appointment if necessary. If the service was engaged when the patient called the call was answered by a receptionist who added the patient to a 'call-back' list and the physiotherapist would return the call when they were free. Patients randomised to usual care were put on the usual service waiting list for face-to-face assessment and treatment.

**Formatted:** Font: 11 pt, Not Italic, Font color: Auto, Pattern: Clear

**Formatted:** Font: 11 pt, Not Italic, Font color: Auto, Pattern: Clear

**Formatted:** Font: 11 pt, Not Italic, Font color: Auto, Pattern: Clear

**Formatted:** Font: 11 pt, Not Italic, Font color: Auto, Pattern: Clear

**Outcome measures**

We used the EQ-5D-3L[284], valued using the UK tariff,[295] to estimate QALYs gained for the cost-utility analysis. The primary outcome for the trial was the physical component summary (PCS) measure



For peer review only - http://bmjopen.bmj.com/site/about/guidelines.xhtml

from the SF-36v2 questionnaire[2630] and secondary clinical outcomes included: the Measure Yourself Medical Outcomes Profile (MYMOP);[2731] a Global Improvement Score – a single question about overall improvement; a composite measure of response to treatment including pain, function and overall improvement (OMERACT OARSI);[2832] patient satisfaction; and waiting time to first treatment advice from a physiotherapist. All outcomes (except the global improvement score and waiting time to first treatment advice) were measured at baseline, 6 weeks and 6 months and were obtained from a self completed questionnaire administered at these three time points.

**Resource use**

The analysis was based on costs related to the reason for which the patient was referred to the physiotherapy service. We identified relevant resources in discussion with participating physiotherapists and service managers. Direct costs to the health care provider included: cost of initial and follow-up physiotherapy consultations; primary and community consultations; hospital care; and prescribed medication. Patient and carer costs included: telephone calls to the PhysioDirect service; travel; over the counter medication; prescription costs; private therapy and purchase of equipment; extra domestic help; and loss of earnings. Lost productivity was estimated separately in relation to time off work to attend physiotherapy appointments and time off because of the musculoskeletal condition itself.

Patient level data about all physiotherapy appointments and consultations were recorded either automatically by computer or by the physiotherapist treating the patient. For those in the intervention group, the PhysioDirect assessment software recorded which physiotherapist conducted each telephone call, and the duration of each call. In addition to the time logged on to the system physiotherapists had to carry out administrative activities following each telephone call, such as collating information to send to the patient by post. The time spent on these activities was



For peer review only - http://bmjopen.bmj.com/site/about/guidelines.xhtml

estimated from information available at one site (Bristol) where manual recording of the entire encounter supplemented the electronic recording.

Physiotherapists assigned to the PhysioDirect service were required to be available throughout the time the service was ‘open’ but they were not usually fully engaged in dealing with PhysioDirect patients during these hours. We conducted an observational time and motion study at each of the four sites to determine how they occupied their non-PhysioDirect contact time in order to apportion costs appropriately. Time and motion data were collected at points in the study when the sites were expected to be fully operational, and across a mix of day, time of day, and location. The capital costs required to run a telephone services are potentially less than for a face-to-face service. Each site provided information about space and equipment required to run their telephone service and we used this to estimate an overall percentage reduction of capital costs for these compared with a standard face-to-face service.

Data about all face-to-face appointments were recorded routinely. These data included the length of appointment, the grade of the physiotherapist seen, and information about missed appointments.

Information about other NHS resource use was collected, where possible, from general practice records and supplemented by information gained directly from patients. General practice notes were scrutinised for patient level data on primary care consultations and prescribed medication. We included all consultations at which the musculoskeletal condition for which the patient was referred to physiotherapy was mentioned and these were recorded by type of consultation (for example, face-to-face, telephone, out of hours, home visit) and by type of professional seen (for example, GP, nurse). It was not feasible to distinguish between medication prescribed for the condition for which the patient was referred for physiotherapy and any other musculoskeletal problem so we included all medication of a potentially relevant type, defined using British National Formulary (BNF)[2933]



For peer review only - http://bmjopen.bmj.com/site/about/guidelines.xhtml

coding. These were: Analgesics (chapters 4.7.1 – 4.7.2); Non-steroidal anti-inflammatory drugs (10.1.1); Local corticosteroid injections (10.1.2.2); and Drugs for the relief of soft-tissue inflammation (10.3).

A questionnaire was administered to participants at 6 weeks and 6 months after randomisation to obtain resource use data not available elsewhere. The questionnaire was designed specifically for this study but was similar in content and structure to others used for the same purpose.[34] Questions These included information about hospital care related to the condition for which the patient was referred to physiotherapy: visits to Accident & Emergency, outpatient appointments, and inpatient stays. Information about personal expenditure relevant to the patient's musculoskeletal condition was also gained from the questionnaire at 6 weeks and 6 months. We asked about the cost of travel to physiotherapy and other health care appointments, expenditure on over the counter medication, prescription costs, use of private therapies and their cost, expenditure on equipment or devices, and extra help at home. In addition, participants were asked about any time off work, and the associated loss of earnings, because of their condition or to attend health care appointments relating to the condition including usual care physiotherapy and PhysioDirect.

Formatted: Superscript

**Valuation of resource use**

Table 1 gives the unit costs and sources used to value the health care resources. We used Curtis[3035] to value primary and community health care and Department of Health reference costs[361] for all hospital-based care. The cost of prescribed medication was estimated from that published in the BNF,[2933] adjusted to allow for the discount available to the NHS, and the professional fee and container allowance in accordance with the Drug Tariff for England.[372] Personal expenditure was reported directly by the participants, the exception being travel by car, which was reported as mileage and costed using the AA schedule of motoring costs.[383] Time off work was valued using the median gross weekly earnings by age and sex.[394]



For peer review only

*Table 1 here*

The cost of face-to-face physiotherapy consultations was estimated by adapting the methods of Curtis[350] to obtain a different unit cost for each band of staff at each site. National median pay rates, by band,[3640] were adjusted to allow for National Insurance, superannuation, and overheads, as per Curtis, then further adjusted to allow for band and site specific non-contact time. Information about the proportion of time physiotherapists on each grade typically spend in direct contact with patients was provided by the four physiotherapy service managers. This provided us with a cost per hour for each band of staff at each site.

The unit cost of physiotherapists working in the PhysioDirect service was estimated in a similar way, but allowing for the reduced cost of capital and overheads; information from the site managers indicated this to be about 50%. To obtain a cost per hour of telephone contact we used information from the computerised records of the PhysioDirect service, which identified the proportion of time spent by physiotherapists actually dealing with PhysioDirect patients. We then combined this with data from the time and motion study, which identified activities undertaken during non-contact time, for example, administration relating to face-to-face appointments or general administration, to give a cost per hour for each band of staff at each site.

All costs were valued in £ sterling at 2009 prices, adjusted for inflation where necessary.[350]



For peer review only - http://bmjopen.bmj.com/site/about/guidelines.xhtml

**Data analysis**

We investigated the amount of each resource used by patients in each group using frequencies, means and medians. Mean total cost per participant was derived by combining resource use with unit costs.

QALYs were derived from responses to the EQ-5D-3L at baseline, 6 weeks and 6 months using valuations from the UK general population.[295] These values, representing health-related quality of life on a scale between zero (death) and 1 (best imaginable health), were used to compute QALYs experienced over the 6 month period using the area under the curve approach and adjusting for any difference between the groups at baseline.[3743]

A cost-consequences matrix was constructed using all available data. We compared costs from all three perspectives (health care provider, patients and carers, lost productivity) with the SF-36v2 PCS, MYMOP, Global Improvement Score, OMERACT OARSI, patient satisfaction, waiting time, and QALYs.

The cost-utility analysis was carried out using data on all patients for whom we had complete NHS cost and QALY data. An incremental cost-effectiveness ratio (ICER) was constructed, comparing the difference in mean total cost per patient with mean difference in QALYs, thus the lower the ICER, the greater the cost-effectiveness and the better the value for money. Uncertainty around the ICER was captured by using the bootstrapping technique: 5000 replicates of the cost and QALY data were created by sampling from the original data, with replacement. The range and spread of the 5000 ICERs was used to replicating it 5000 times using the bootstrapping technique, construct a cost-effectiveness acceptability curve to indicate the likelihood of the intervention being cost-effective. The net monetary benefit (NMB) of the intervention was estimated from the point estimate of the ICER for values of societal willingness-to-pay of £20,000 and £30,000 per QALY. If the NMB is positive



For peer review only - http://bmjopen.bmj.com/site/about/guidelines.xhtml

at a given level of willingness to pay, the intervention is regarded as cost-effective. Confidence intervals around the NMB were formed from the bootstrapped estimates. estimating a confidence interval around the net monetary benefit, and constructing a cost-effectiveness acceptability curve.

We used the multiple imputation by chained equation procedure to address the issue of missing cost and EQ-5D data.[3844] This technique uses a regression model to estimate missing values from known values. In addition to cost and EQ-5D-3L variables the imputation model also included randomisation group, age, sex, and SF36v2 PCS. Stata v12[3945] was used to generate five datasets using 10 switching procedures.

Discounting was not carried out because the analysis was restricted to costs and outcomes over a period of less than a year. All analyses were conducted using Microsoft Excel and Stata v12.[3944]

**Sensitivity Analyses**

We addressed three areas of uncertainty using four one/two-way sensitivity analyses. First, we estimated the cost of running the PhysioDirect service if it was operating at full capacity. It is likely that this was not achieved during the trial because of: low demand due to exclusions and non-participation in the trial; inflexible staffing levels to ensure consistency throughout the trial period; and the ‘one-way’ system generally used, where physiotherapists waited for patients to call them but did not routinely contact patients themselves (notwithstanding some limited use of answer-machines). Data from the Bristol service, which continued to operate beyond the trial period and was then able to tailor staffing levels to demand, were used to estimate the cost of running a more ‘efficient but feasible’ PhysioDirect service once the trial had ended.



For peer review only - http://bmjopen.bmj.com/site/about/guidelines.xhtml

The second area of uncertainty addressed hospital costs. Patients in the trial were recruited from primary care and for these~~,~~ use of secondary care is infrequent but relatively expensive and this can have a disproportionate effect on mean total cost. We tested this by excluding hospital costs from the total.

The third area of uncertainty tested the effect of using imputed data rather than complete cases; the third ~~and fourth~~ sensitivity analysies used ~~the~~ trial ~~imputed missing~~ data with missing values imputed.

Finally, in a two-way sensitivity ~~as described above first with data collected during the trial and~~ analysis, we re-estimated ~~second by comb~~the ~~ining with~~ results of the first ~~sensitivity analysis~~, (mimicking an 'efficient but feasible' service) in this instance using the imputed dataset. ~~.~~

**RESULTS**

A total of 2,249 patients were recruited between July 2009 and December 2009, and followed up until June 2010~~took part in the trial~~, 1506 allocated to PhysioDirect and 743 to usual care. The mean age was 60, with slightly more females than males (60% vs 40%); they were overwhelmingly white (97%), just over half (60%) were employed and all but a few were referred for physiotherapy by their GP. Lower limb problems were the most prevalent (30%) reason for referral, 27% patients had a lumbar problem and 23% upper limb problems. Nearly all participants (2,223=99%) gave permission to access their GP notes to obtain data about primary care encounters and prescribed medication. 81% returned questionnaires at both 6 weeks and 6 months though not all participants completed all sections at both time points. We had complete NHS cost and QALY data for 840 (56%) PhysioDirect and 432 (58%) usual care participants.



For peer review only

For peer review only - http://bmjopen.bmj.com/site/about/guidelines.xhtml

**Resource use**

Table 2 gives information about the different types of physiotherapy consultations by patients in each group. Of the 1506 patients in the PhysioDirect group, 39% (586) were managed solely over the telephone and only 46% (695) had any face-to-face consultations. In total, patients in the usual care group had, on average, 0.38 (95% CI: 0.12 to 0.63) more consultations than those in the PhysioDirect group and the mean total duration of all consultations was 20 minutes longer (95% CI: 12 to 28).

*Table 2 here*

Tables 3 and 4 give information about NHS and personal resource use. Just over a third of patients (35%) had a GP consultation during the 6 months and 40% received a prescription for musculoskeletal pain-related medication. There was very little difference between the two groups in terms of health care use and the only ~~important~~ notable difference in personal expenditure was travel to physiotherapy appointments.

*Tables 3 and 4 here*

**Costs and consequences**

Table 5 summarises the mean cost per patient, by group, for each category of cost. All available data are included giving variable denominators for each category. Comparing the two groups, there are small differences in cost in some categories but for most of these the confidence intervals indicate there is no evidence of a difference between the groups.

*Table 5 here*



For peer review only - http://bmjopen.bmj.com/site/about/guidelines.xhtml

Table 6 combines the results of the cost analysis with the full range of primary and secondary outcomes, including QALYs.

*Table 6 here*

Results are presented for all available data, with cost categories combined. DAgain, denominators vary within the table and they also differ from those in table 5 because sub-categories have been collapsed. There was no evidence of a difference in the primary clinical outcome (the SF36v2 PCS) between the groups, suggesting that PhysioDirect led to similar outcomes as usual physiotherapy care. Patients in the PhysioDirect group had their first assessment and telephone advice 27 days earlier than those in the usual care group, however patient satisfaction was slightly lower in those receiving PhysioDirect. QALYs were higher in the PhysioDirect group by 0.009, which equates to about 3.3 extra days of full health over a year.

**Cost-utility analysis**

The cost-utility analysis presented in Table 7 uses complete cases, that is, we include only those patients for whom we had complete NHS cost and QALY data: 432 (58%) from the usual care group and 840 (56%) from PhysioDirect. The small extra cost of caring for patients in the PhysioDirect group was compensated for by the extra QALY gain, giving an incremental cost-effectiveness ratio of £2,889. Values below £20,000 are regarded by NICE to indicate a cost-effective intervention[27] At this threshold level of If society is willingness to pay for a £20,000 per QALY[23] there is a positive -net monetary benefit of is £117 (95% CI -£96 to £310) and there is 0.88 probability that the intervention is cost-effective. This is illustrated in the cost-effectiveness acceptability curve in figure 1.

**Formatted:** Superscript

*Table 7 here*

*Figure 1 here*

17

For peer review only

**Sensitivity Analysis**

The results of the four sensitivity analyses are shown in table 8 and figure 2. Scenario (1) indicates the potential cost-effectiveness of a more efficient PhysioDirect service. During the trial PhysioDirect clinic opening hours, physiotherapists spent about 35% of their time on the phone or dealing with directly related administration; in Bristol after the trial, this was increased to 57%. Under this scenario, the cost per patient in the PhysioDirect group was £14.53 less than under trial conditions and £2.11 less per patient in the usual care group. The ICER is therefore lower, at £1,045, the NMB correspondingly higher at £127 (λ=£20,000). At low levels of λ, the probability of PhysioDirect being cost-effective under this scenario is higher than with the base case, though at λ=£20,000 it reaches a similar value (see figures 1 and 2).

*Table 8 here*

*Figure 2 here*

The effect of removing hospital costs from the analysis is shown in sensitivity analysis (2). Hospital costs accounted for 75% of all NHS costs yet only 19% (n=252) participants reported using any secondary care. Hospital use was evenly divided between the two groups so removing these from the analysis made very little difference to incremental analysis.

The effect of imputing missing NHS cost and QALY data is explored in sensitivity analysis (3). Using these data the cost of the interventions is lower but this is offset by higher NHS costs, giving a higher mean total cost in both groups, by £21.41 in the usual care group and £6.58 in the PhysioDirect group. QALYs using imputed data are lower, by 0.005 in the usual care group and by 0.010 in the PhysioDirect group. The net effect is a reduction of both incremental cost and incremental QALYs,



For peer review only - http://bmjopen.bmj.com/site/about/guidelines.xhtml

giving an ICER of £2,260. Uncertainty around the ICER is reduced, as seen by the flatter CEAC in figure 2.

Sensitivity analysis (4) combines analyses (1) and (3) by using imputed cost data in the 'efficient service' scenario. In this case the results indicate that PhysioDirect is, on average, cheaper than usual care with a possible saving of £6.02 per patient, which gives a negative value for the ICER, indicating the intervention is superior in terms of both cost and outcome. The results suggest a possible cost saving of £6.02 per patient; the probability that the service is cost-effective at λ=£20,000 is 0.72.

**DISCUSSION**

**Statement of principal findings**

The results of this economic evaluation suggest that PhysioDirect services for patients with musculoskeletal problems require careful management if they are to be a cost-effective alternative to usual physiotherapy care. There was very little difference between the two groups in terms of either outcomes or costs, and the finding that PhysioDirect is cost-effective is based on evidence that it provides very slightly greater QALY benefits at very slightly greater cost.

Clearer cost savings were observed in the sensitivity analysis that replicated the post-trial service, once greater flexibility in working arrangements was implemented. Without the restrictions of a trial environment staffing was adjusted to meet the anticipated demand, a call-back service was employed which accommodated fluctuations in activity during each session, referrals added to the system were adjusted regularly to reflect actual staffing and the number of patients waiting for a call-back and a higher throughput of patients led to greater economies of scale. These changes ensured physiotherapists within the PhysioDirect service spent a higher proportion of their PhysioDirect clinic time on the telephone with patients. There was no evidence of a difference



For peer review only - http://bmjopen.bmj.com/site/about/guidelines.xhtml

between PhysioDirect and usual care in cost to patients and their families, or to society through the costs of lost production.

**Strengths and weaknesses of the study**

The study has a number of strengths. It is the first study assessing the cost-effectiveness of a PhysioDirect service including a large sample of patients with a wide range of musculoskeletal problems based across a number of locations.[1~~54~~] It uses a rigorous study design and conforms to CONSORT guidelines.[4~~60~~] The f~~F~~ollow up rate from participating patients was in line with other primary care trials~~good~~,[47,48] a high proportion of the resource use data were collected from GP records, and there was collection of resource use information outside of the main health service perspective. ~~Nevertheless, there are also limitations.~~ The cost consequences analysis provides complete information on costs from different perspectives compared with a range of outcomes so although this approach is sometimes criticised for leaving the reader to evaluate the findings it does have the advantage of transparency. Furthermore, in this study we have also presented a cost-utility analysis that conforms to the recommendations of NICE. ~~The proportion of eligible individuals consenting to participate in the trial was only 50%.~~[~~22~~] Nevertheless, there are also limitations. The practices recruited to the trial had a low proportion of ethnic minority patients, a slightly lower proportion of patients from deprived areas were judged to be eligible, and the proportion of eligible individuals consenting to participate in the trial was only 50%.[23] These factors limit the generalisability of the results though none of these selection effects was large. Further, a particular difficulty in conducting economic evaluation with new service developments is ensuring that they are fully utilised,~~.~~[~~41~~24] particularly when conducting analysis from a long-run perspective, as here. Although there was a clear run-in period prior to data collection for the trial (ranging from 4 to 12 weeks in each of the four sites), to ensure that services were operating as well as they could, and a 2:1 randomisation ratio in favour of PhysioDirect was used, there was still considerable underutilisation of the new service. This was ameliorated by including a more fully utilised service

**Formatted:** Superscript

**Formatted:** Check spelling and grammar, Not Superscript/ Subscript



For peer review only - http://bmjopen.bmj.com/site/about/guidelines.xhtml

within the sensitivity analysis. It should also be noted that, because of the use of a long-run perspective in the analysis, set up costs, which may, in the short-term be important in a financially constrained service, are not included here. Finally, because the differences in both costs and effects are small, there is still some uncertainty around the findings.

**The meaning of the study and implications for policy makers**

If the aim of health services is to achieve maximum health gain from an investment in health care, then PhysioDirect has a high probability of being more cost-effective than usual physiotherapy care. This research also, however, suggests that it cannot be assumed that PhysioDirect will reduce costs, although it could potentially do so if PhysioDirect services are managed efficiently. Both the costs and cost-effectiveness of services will depend on the productivity of physiotherapist time. If physiotherapists are able to use most of their time dealing directly with patients during sessions when they are available on the telephone, then the service will be less costly. This is most likely to be achieved by operating a call-back service and/or by operating the service on a large scale to even out fluctuations in demand. Operating a system on  larger scale might also offer other economies of scale, particularly in terms of infrastructure. Further efficiencies may also be achieved if these services are, in the future, provided in conjunction with direct access for patients (rather than following referral from another health care professional), given that patients who self-refer are likely to contact the service with musculoskeletal problems of shorter duration,[492] and such patients may be particularly appropriate for the initial assessment and advice provided by a PhysioDirect service. More generally, the study has broader implications for telephone services, particularly around the implicit assumption that such services will inevitably be money saving. Here this assumption was found to be false, largely because the physiotherapists' time was underutilised during PhysioDirect clinic hours. Thus, for all such services, it will be important for policy makers to ensure that easy assumptions about the costs of these services are properly assessed in relation to factors such as



For peer review only

how efficiently the service is run, and what proportion of patients are subsequently invited for face to face care following an initial telephone call.

**Unanswered questions and future research**

As services evolve, further research should explore the costs and benefits of PhysioDirect under different scenarios. These might include: comparing different skill levels of staff operating the service; the inclusion of patient self-referrals in addition to GP referrals; the use, or otherwise, of computerised support in assessing the patient; the extension to internet services (possibly combined with cameras);[~~43~~50] and the use of mobile ‘smartphone’ technology, for example in rapid assessment of musculoskeletal injuries. In particular, however, it will be important to assess the costs and benefits of services once they are more established and provided on a wider scale. The costs and benefits of telehealth more generally need further exploration in relation to their cost effectiveness particularly given the negative findings of the Whole Systems Demonstrator project evaluating telehealth support and treatment for patients with long term conditions.[~~44~~51] It would be helpful to identify those characteristics that are likely to make services both more cost-effective and less costly.

For peer review only



For peer review only - http://bmjopen.bmj.com/site/about/guidelines.xhtml

## References


1. Munro J, Nicholl, J, O'Cathain A et al. Impact of NHS Direct on demand for immediate care: observational study. *Br Med J* 2000;321:150-153.

2. Lattimer V, Sassi F, George S et al. Cost analysis of nurse telephone consultation in out of hours primary care: evidence from a randomised controlled trial. *Br Med J* 2000;320:1053-1057.

3. Lattimer V, George S, Thompson F et al. Safety and effectiveness of nurse telephone consultation in out of hours primary care: randomised controlled trial. The South Wiltshire Out of Hours Project (SWOOP) Group. *Br Med J* 1998;317:1054-1059.

4. Pinnock H, Bawden R, Proctor S et al. Accessibility, acceptability, and effectiveness in primary care of routine telephone review of asthma: pragmatic, randomised controlled trial. *Br Med J* 2003;326:477-479.

5. Bunn F, Byrne G, Kendall S. Telephone consultation and triage: effects on health care use and patient satisfaction.*Cochrane Database of Systematic Reviews.(4):CD004180, 2004.* 2004;CD004180.

6. McKinstry B, Watson P, Pinnock H et al. Telephone consulting in primary care: a triangulated qualitative study of patients and providers. *Br J Gen Pract* 2009;59:e209-e218.

7. Picavet HS, Schouten JS. Musculoskeletal pain in the Netherlands: prevalences, consequences and risk groups, the DMC(3)-study. *Pain* 2003;102:167-178.

8. Jordan K, Ong BN, Croft P. Previous consultation and self reported health status as predictors of future demand for primary care. *J Epidemiol Commun Health* 2003;57:109-113.

9. McKormick A, Fleming D, and Charlton J *Morbidity statistics from general practice. Fourth national study 1991-1992 Serise MB5 no.3*. London: 1995.




For peer review only

For peer review only - http://bmjopen.bmj.com/site/about/guidelines.xhtml

10. Balage F, Mannion A, Pellise F, et al. Non-specific low back pain. *Lancet* 2012;379(9814):482-91.

~~Andersson GB. Epidemiological features of chronic low-back pain. *Lancet* 1999; 354: 581-585.~~

11. Chuang L-H, Soares M, Tilbrook H, et al. A Pragmatic multicentred randomized controlled trial of yoga for chroonic low back pain: economic evaluation. *Spine* 2012;37(18):1593-1601.

~~Maniadakis N, Gray A. The economic burden of back pain in the UK. *Pain* 2000;84:95-103.~~

12. National Institute for Health and Clinical Excellence *Low back pain: early management of persistent non-specific low back pain. NICE clinical guideline 88*. 2009.

13. Department of Health. Physiotherapy Services Summary Information for 2004-2005, England https://catalogue.ic.nhs.uk/publications/hospital/outpatients/nhs-phys-serv-summ-eng-2004-05/nhs-phys-serv-summ-eng-2004-05-rep.pdf ~~http://www.hscic.gov.uk/searchcatalogue?productid=4306&kwd=K&gran=Government+Office+Regions&sort=Relevance&size=10&page=1#top~~ (accessed 16~~0~~.08~~6~~.2013)

14. Maniadakis N, Gray A. The economic burden of back pain in the UK. *Pain* 2000;84:95-103.

15~~4~~. Salisbury C, Montgomery AA, Hollinghurst S et al. Effectiveness of PhysioDirect telephone assessment and advice services for patients with musculoskeletal problems: pragmatic randomised controlled trial. *Br Med J* 2013;346:f43.

16~~5~~. Hodgson K. Kidderminster helpline proves a success. *Physiotherapy Frontline* 2001;7:22-23.

17~~6~~. Taylor S, Ellis I, and Gallagher M. Patient satisfaction with a new physiotherapy telephone service for back pain patients. *Physiotherapy* 2002;88:645-657.

**Formatted:** Font: Italic

**Formatted:** Font: Italic

**Formatted:** Indent: Hanging: 0.25"

**Formatted:** Indent: Hanging: 0.25"

**Formatted:** Default Paragraph Font



For peer review only - http://bmjopen.bmj.com/site/about/guidelines.xhtml

187. Clayson M and Woolvine M. Back pain direct clinic: a collaboration between general practitioners and physiotherapists. *Work Based Learning in Primary Care* 2004;2:38-43.

198. Turner D. An exploratory study of physiotherapy telephone assessment. *International Journal of Therapy and Rehabilitation* 2009;16:97-105.

2019. Russell T, Truter P, Blumke R, Richardson B. The diagnostic accuracy of telerehabilitation for nonarticular lower-limb musculoskeletal disorders. *Telemed J & E-Health* 2010;16:585-594.

210. Patel S, Hossain FS, Colaco HB et al. The accuracy of primary care teams in diagnosing disorders of the shoulder. *J Eval Clin Pract* 2011;17:118-122.

221. Jette DU, Ardleigh K, Chandler K et al. Decision-making ability of physical therapists: physical therapy intervention or medical referral. *Phys Ther* 2006;86:1619-1629.

232. Salisbury C, Foster NE, Hopper C et al. A pragmatic randomised controlled trial of the effectiveness and cost-effectiveness of 'PhysioDirect' telephone assessment and advice services for physiotherapy. *Health Technology Assessment (Winchester, England)* /20;17:1-157.

~~23. National Institute for Health and Clinical Excellence. Guide to the Methods of Technology Appraisal http://www.nice.org.uk/media/B52/A7/TAMethodsGuideUpdatedJune2008.pdf (accessed 10.06.2013)~~

2411. —Coast J, Hensher M, Mulligan J, et al. Conceptual and practical difficulties with the economic evaluation of health services developments. J *Health Serv Res Policy* 2000;5:42-48.

25. Coast, J. Is economic evaluation in touch with society's health values? *Br Med J* 2004;(329):1233-1236.


Formatted: Font: Italic, No underline, Font color: Auto



For peer review only - http://bmjopen.bmj.com/site/about/guidelines.xhtml

26. Drummond M, Brown R, Fendrick M, et al. Use of Pharmacoeconomics Information—Report of the ISPOR Task Force on Use of Pharmacoeconomic/Health Economic Information in Health-Care Decision Making. *Value in Health* 2003;6 (4):407-416.

27. National Institute for Health and Clinical Excellence. Guide to the Methods of Technology Appraisal http://www.nice.org.uk/media/B52/A7/TAMethodsGuideUpdatedJune2008.pdf (accessed 16.08.2013)

284. Brooks R, with the EuroQol Group. EuroQol: the current state of play. *Health Policy* 1996;53-72.

295. Dolan P, Gudex C, Kind P et al. *A social tariff for EuroQol: Results from a UK general population survey*. University of York, 1-9-1995.

3026. Quality Metric. A community for measuring health outcomes using SF tools. http://www.sf-36.org/ (accessed 160.086.2013)

3127. Paterson, C. Measuring outcomes in primary care: a patient generated measure, MYMOP, compared with the SF-36 survey. *Br Med J* 1996;312:1016-1020.

3228. Pham T, Van Der Heijde D, Lassere M et al. Outcome variables for osteoarthritis clinical trials: The OMERACT-OARSI set of responder criteria. *J Rheumatol* 2003;30:1648-1654.

3329. British National Formulary. British National Formulary. http://www.bnf.org/bnf (accessed 160.086.2013)

34. Database of Instruments for Resource Use Measurement (DIRUM) http://www.dirum.org (accessed 16.08.2013)

350. Curtis L. *Unit costs of health and social care 2009*. PSSRU, University of Kent, 2010.


**Formatted:** Indent: Left: 0"

**Formatted:** Font: Italic, No underline, Font color: Auto

**Formatted:** Indent: Left: 0", Hanging: 0.25"

**Formatted:** Indent: Left: 0", Hanging: 0.25"

**Formatted:** Default Paragraph Font, Font: (Default) Calibri, 11 pt, No underline, Font color: Auto, Do not check spelling or grammar, Pattern: Clear

**Formatted:** Default Paragraph Font, Font: (Default) Calibri, 11 pt, No underline, Font color: Auto, Do not check spelling or grammar, Pattern: Clear

**Formatted:** Default Paragraph Font, Font: (Default) +Body (Calibri), Check spelling and grammar



For peer review only

For peer review only - http://bmjopen.bmj.com/site/about/guidelines.xhtml

3<u>6</u>~~1~~. Department of Health. NHS Reference costs 2009-2010. https://www.gov.uk/government/publications/nhs-reference-costs-2009-2010 (accessed 1<u>6</u>~~0~~.0<u>8</u>~~6~~.2013)

3<u>7</u>~~2~~. National Health Service. NHS Drug tariff. http://www.nhsbsa.nhs.uk/PrescriptionServices/924.aspx (accessed 1<u>6</u>~~0~~.0<u>8</u>~~6~~.2013)

3<u>8</u>~~3~~. The Automobile Association. The AA schedule of motoring costs. http://www.theaa.com/motoring_advice/running_costs/index.html (accessed 1<u>6</u>~~0~~.0<u>8</u>~~6~~.2013)

3<u>9</u>~~4~~. Office for National Statistics. Annual survey of hours and earnings 2009 http://www.ons.gov.uk/ons/rel/ashe/annual-survey-of-hours-and-earnings/2009-revised/index.html (accessed 1<u>6</u>~~0~~.0<u>8</u>~~6~~.2013)

<u>40</u>~~35~~. Royal College of Nursing. Agenda for change – pay rates http://www.nhscareers.nhs.uk/working-in-the-nhs/pay-and-benefits/agenda-for-change-pay-rates (accessed 1<u>6</u>~~0~~.0<u>8</u>~~6~~.2013)

<u>41</u>~~36~~. Scott A, Simoens S, Heaney D, et al. What does GP out of hours care cost? An analysis of different models of out of hours care in Scotland. *Scot Med J* 2004;49:61-66.

<u>42. O'Dowd A. Cost of out of hours care was 22% higher than predicted in England. *Br Med J* 2006;332:1113.4.</u>

<u>43</u>~~37~~. Manca A, Hawkins N, Sculpher M. Estimating mean QALYs in trial-based cost-effectiveness analysis: the importance of controlling for baseline utility. *Health Econ* 2005;14:487-496.

<u>44</u>~~38~~. van Buuren S, Boshuizen HC, Knook D L. Multiple Imputation of missing blood pressure covariaties in survival analysis. *Stat Med* 1999;18:681-694:

<u>45</u>~~39~~. StataCorp *Statistical Software: Release 11. College Station, TX: StataCorp LP.* 2009.

**Formatted:** Font: Italic, No underline, Font color: Auto



For peer review only

46~~0~~. The CONSORT Group. CONSORT: the transparent reporting of trials http://www.consort-statement.org (accessed 16~~0~~.08~~6~~.2013)

47. Stamuli E, Bloor K, MacPherson H, et al. Cost-effectiveness of acupuncture for irritable bowel syndrome: findings from an economic evaluation conducted alongside a pragmatic randomised controlled trial in primary care. BMC Gastroenterology 2012; 12:149.

48. Hill JC, Whitehurst DG, Lewis M, et al. Comparison of stratified primary care management for low back pain with current best practice (STarT Back): a randomised controlled trial. Lancet 2011; 378 (9802): 1560-71.

~~41. Coast J, Hensher M, Mulligan J, et al. Conceptual and practical difficulties with the economic evaluation of health services developments. J *Health Serv Res Policy* 2000;5:42-48.~~

49~~2~~. Leemrijse CJ, Swinkels IC, Veenhof C. Direct access to physical therapy in the Netherlands: results from the first year in community-based physical therapy. *Phys Ther*. 2008;~~-~~88(8):936-46

50~~43~~. Li LC, Townsend AF, Badley EM. Self-management interventions in the digital age: new approaches to support people with rheumatologic conditions. *Best Pract Res Clin Rheumatol*. 2012;~~-~~26(3):321-33.

51~~44~~. Henderson C, Knapp M, Ferbnandez J-L, et al. Cost effectiveness of telehealth for patients with long term conditions (Whole Systems Demonstrator telehealth questionnaire study): nested economic evaluation in a pragmatic, cluster randomised controlled trial. *Br Med J* 2013;34.

j2065.

**Formatted:** Font: (Default) Calibri, No underline, Font color: Auto, Do not check spelling or grammar

**Formatted:** Font: (Default) Calibri, 11 pt, No underline, Font color: Auto, Do not check spelling or grammar

**Formatted:** Normal, Indent: Left: 0", Hanging: 0.25", Space After: 12 pt, Line spacing: Double, Tab stops: 0.25", Left

**Formatted:** Font: (Default) Calibri, 11 pt, Not Bold, No underline, Font color: Auto, Do not check spelling or grammar

**Formatted:** Font: (Default) Calibri, 11 pt, No underline, Font color: Auto, Do not check spelling or grammar

**Formatted:** Font: (Default) Calibri, No underline, Font color: Auto, Do not check spelling or grammar

**Formatted:** Font: (Default) Calibri, 11 pt, Not Italic, No underline, Font color: Auto, Do not check spelling or grammar

**Formatted:** Font: (Default) Calibri, 11 pt, Not Italic, No underline, Font color: Auto, Do not check spelling or grammar

**Formatted:** Font: (Default) Calibri, Do not check spelling or grammar

**Formatted:** Font: (Default) Calibri, No underline, Font color: Auto, Do not check spelling or grammar

**Formatted:** Indent: Left: 0", Hanging: 0.25"

**Formatted:** Font: (Default) Calibri, 11 pt, Not Bold, No underline, Font color: Auto, Do not check spelling or grammar

**Formatted:** Font: (Default) Calibri, 11 pt, No underline, Font color: Auto, Do not check spelling or grammar

**Formatted:** Font: (Default) Calibri, 11 pt, No underline, Font color: Auto, Do not check spelling or grammar

**Formatted:** Font: (Default) Calibri, No underline, Font color: Auto, Do not check spelling or grammar

**Formatted** ... [1]

**Formatted** ... [2]

**Formatted** ... [3]

**Formatted** ... [4]

**Formatted:** Indent: Left: 0", Hanging: 0.25"

**Formatted** ... [5]

**Formatted:** Font: Italic, No underline, Font color: Auto

**Formatted:** Font: Italic, No underline, Font color: Auto

**Formatted:** Indent: Hanging: 0.25"

**Formatted:** Font: Italic, No underline, Font color: Auto

**Formatted** ... [6]



For peer review only

For peer review only - http://bmjopen.bmj.com/site/about/guidelines.xhtml

**Competing interestes**

We have read and understood the BMJ Group policy on declaration of interests and declare the following interests: none.

**Funding**

The research was funded by the Medical Research Council (MRC) and managed by the National Institute for Health Research (NIHR) on behalf of the MRC-NIHR partnership. The funder had no role in: the study design; the collection, analysis, or interpretation of data; the writing of the report; the decision to submit the paper for publication. The researchers are all independent of the funders. All researchers had access to all the data.

Nadine Foster is supported by a National Institute for Health Research NIHR Research Professorship NIHR-RP-011-015. The views expressed in this publication are those of the authors and not necessarily those of the NHS, the National Institute for Health Research or the Department of Health.

**Aurthors' contributions**

CS was principal investigator on the trial, SH took the lead on the economic evaluation. JC, NF, SG, JH, and AM were co-applicants and contributed to the conception and design. SH, JC and JB carried out the analysis with help from CS and AM, and all authors contributed to the interpretation of the data. SH and JC wrote the first draft of the paper with all authors contributing to subsequent revisions. All authors have seen and approved the final version.

**Acknowledgments**

**We would like to dedicate this paper to the memory of Cherida Hopper, trial manager of the PhysioDirect study, who sadly died in June 2013.**

We would like to thank the patients who contributed to this research; the physiotherapists, administrative staff, managers and commissioners who supported the set up and delivery of the trial in the four primary care trusts; participating general practices; the research support staff in Bristol and Keele; members of the Trial Steering Committee and Data Monitoring Committee; and Jill



For peer review only - http://bmjopen.bmj.com/site/about/guidelines.xhtml

Gamlin and Nick Deane who developed the PhysioDirect assessment algorithms and software used in this trial.

For peer review only



For peer review only - http://bmjopen.bmj.com/site/about/guidelines.xhtml

**Table 1: Data sources and unit costs**

| | Unit cost (£) |
|---|---|
| **Primary and Community Care**[350] | |
| General Practitioner | |
| Surgery | 27.00 |
| Telephone consultation | 16.00 |
| Home visit | 91.00 |
| Practice nurse | |
| Surgery | 10.00 |
| Telephone consultation | 5.93 |
| Health Care Assistant/phlebotomist | |
| Surgery | 6.92 |
| District Nurse | |
| Home visit | 16.33 |
| **Out of Hours**[41,42] | |
| General practitioner | 23.50 |
| **Hospital care**[361] | |
| A&E | 103.00 |
| Outpatient visits | By Healthcare Resource Group, differentiated by first and follow-up |
| Inpatient stays | By Healthcare Resource Group |
| **Prescribed medication**[2933] | per item, by name, strength and amount |
| **Mileage**[383] | 0.4612 |
| **Time off work**[394] | Median national wage by age and sex |



For peer review only - http://bmjopen.bmj.com/site/about/guidelines.xhtml

**Table 2: Number of physiotherapy consultations and mean duration, by type and group**

| | | Usual care (n=743) | PhysioDirect (n=1506) |
|---|---|---|---|
| **Face-to-face appointments** | mean (SD) number | 3.11(2.63) | 1.91(2.72) |
| | mean (SD) total duration (minutes) | 107.51(88.92) | 64.20(89.31) |
| **Telephone appointments** | mean (SD) number | 0.13(0.44) | 0.96 (0.63) |
| | mean (SD) total duration (minutes) | 4.21(14.64) | 27.37(19.92) |
| **Home visits** | mean (SD) number | 0.00 (0.06) | 0.00 (0.06) |
| | mean (SD) total duration (minutes) | 0.14(2.27) | 0.12(2.12) |
| **All physiotherapy contacts** | mean (SD) number | 3.25(2.70) | 2.87(2.94) |
| | mean (SD) total duration (minutes) | 111.86(90.50) | 91.70 (95.40) |



For peer review only

**Table 3: Health services resource use, by group. All available data.**

| | Mean (SD) number of consultations | | | |
|---|---|---|---|---|
| | **n** | **usual care** | **n** | **PhysioDirect** |
| GP consultations | 739 | 0.77(1.47) | 1484 | 0.87 (1.68) |
| Nurse consultations | 739 | 0.04 (0.22) | 1484 | 0.06 (0.32) |
| Other primary care consultations | 739 | 0.02(0.14) | 1484 | 0.02 (0.17) |
| **Total number of primary care contacts** | **739** | **0.83(1.56)** | **1484** | **0.96 (1.84)** |
| Number of prescriptions | 728 | 1.36 (2.73) | 1469 | 1.68 (3.72) |
| A&E (visits) | 467 | 0.02 (0.01) | 912 | 0.03 (0.01) |
| Out-patient (consultations) | 467 | 0.17 (0.83) | 910 | 0.35 (1.03) |
| In-patient stays (Finished Consultant Episodes) | 465 | 0.01 (0.10) | 910 | 0.01 (0.10) |



For peer review only - http://bmjopen.bmj.com/site/about/guidelines.xhtml

**Table 4: Patient and societal resource use, by group. All available data.**

| Number (%) reporting: | n | usual care | n | PhysioDirect |
|---|---|---|---|---|
| Expenditure on travel to physiotherapy | 462 | 242 (52.4) | 1232 | 308 (25.8) |
| Expenditure on travel to primary care | 669 | 116 (17.3) | 1337 | 237 (17.7) |
| Expenditure on over-the-counter medication | 506 | 256 (50.6) | 1028 | 512 (49.8) |
| Expenditure on prescriptions | 559 | 264 (47.2) | 1085 | 508 (46.8) |
| Expenditure on private therapy | 484 | 89 (18.4) | 934 | 167 (17.9) |
| Equipment purchase | 480 | 139 (29.0) | 939 | 233 (24.8) |
| Payments for extra domestic help | 459 | 35 (7.6) | 928 | 76 (8.2) |
| Loss of earnings | 598 | 30 (5.5) | 1209 | 64 (5.3) |
| Any time off to attend physiotherapy consultation | 692 | 218 (31.5) | 1416 | 380 (26.8) |
| Work has been affected because of condition | 477 | 141 (29.6) | 959 | 317 (33.1) |



For peer review only

**Table 5: Mean total cost per patient, by group and category. All available data[a].**

| | ~~n~~ usual care | ~~mean (SD) cost~~ | ~~N~~ PhysioDirect | ~~mean (SD) cost~~ | Incremental difference (95% Confidence Interval) |
|---|---|---|---|---|---|
| | n | mean (SD) cost | n | mean (SD) cost | |
| **Physiotherapy services** | | | | | |
| Face-to-face appointments | 743 | £64.42 (£53.00) | 1506 | £38.76 (£53.92) | -£25.66 (-£30.37 to -£20.95) |
| Telephone appointments | 743 | £5.22 (£18.01) | 1506 | £35.17 (£26.34) | £29.94 (£27.84 to £32.05) |
| Home visits | 743 | £0.08 (£1.33) | 1506 | £0.08 (£1.46) | £0.00 (-£0.12 to £0.13) |
| **Total physiotherapy cost** | **743** | **£69.73 (£56.17)** | **1506** | **£74.01 (£63.97)** | **£4.28 (-£1.12 to £9.69)** |
| **Primary care services** | | | | | |
| GP consultations | 739 | £19.21 (£35.91) | 1484 | £21.69 (£41.66) | £2.48 (-£1.04 to £6.00) |
| Nurse consultations | 739 | £0.44 (£2.37) | 1484 | £0.61 (£3.17) | £0.16 (-£0.10 to £0.42) |
| Other primary care consultations | 739 | £0.03 (£0.57) | 1484 | £0.07 (£1.31) | £0.05 (-£0.05 to £0.15) |
| **Total primary care cost** | **739** | **£19.68 (£36.68)** | **1484** | **£22.37 (£42.83)** | **£2.69 (-£0.92 to £6.30)** |
| **Medication cost** | 728 | £11.04 (£51.61) | 1469 | £10.33 (£55.43) | -£0.72 (-£5.53 to £4.10) |
| **Hospital services** | | | | | |
| A&E | 467 | £1.99 (£17.12) | 912 | £3.17 (£20.84) | £1.18 (-£1.01 to £3.37) |
| Out-patient | 467 | £30.74 (£98.36) | 910 | £38.35 (£126.05) | £7.61 (-£5.50 to £20.72) |
| In-patient | 465 | £51.02 (£520.48) | 910 | £34.99 (£399.62) | -£16.03 (-£65.70 to £33.64) |
| **Total hospital cost** | **459** | **£83.04 (£561.68)** | **899** | **£77.00 (£446.24)** | **-£6.04 (-£60.99 to £48.91)** |
| **Personal expenditure** | | | | | |
| Cost of all calls to physiotherapy service | 743 | £0.97 (£0.99) | 1506 | £1.75 (£1.29) | £0.79 (£0.68 to £0.89) |
| Travel to physiotherapy | 462 | £6.11 (£11.48) | 1232 | £3.11 (£8.51) | -£3.01 (-£4.01 to -£2.00) |
| Travel for primary care | 669 | £0.65 (£2.93) | 1337 | £0.75 (£4.10) | £0.11 (-£0.24 to £0.45) |
| Over-the-counter medication | 490 | £7.67 (£14.09) | 987 | £8.61 (£22.38) | £0.94 (-£1.23 to £3.11) |
| Cost of prescriptions | 553 | £2.72 (£8.95) | 1076 | £2.67 (£8.33) | -£0.05 (-£0.93 to £0.82) |

**Formatted:** Line spacing: Multiple 1.25 li
**Formatted:** Centered, Line spacing: Multiple 1.25 li
**Formatted:** Centered, Line spacing: Multiple 1.25 li
**Formatted:** Line spacing: Multiple 1.25 li
**Formatted:** Line spacing: Multiple 1.25 li
**Formatted:** Line spacing: Multiple 1.25 li
**Formatted Table**



For peer review only - http://bmjopen.bmj.com/site/about/guidelines.xhtml

| | | | | | |
|---|---|---|---|---|---|
| Private therapy | 475 | £21.98 (£70.34) | 915 | £39.34 (£296.94) | £17.36 (-£9.76 to £44.48) |
| Equipment purchase | 473 | £17.16 (£169.12) | 924 | £9.12 (£56.42) | -£8.04 (-£20.08 to £4.00) |
| Extra domestic help | 451 | £10.93 (£64.31) | 905 | £13.68 (£96.02) | £2.75 (-£7.07 to £12.56) |
| Cost associated with loss of earnings | 598 | £46.69 (409.72) | 1209 | £82.78 (£885.85) | £36.09 (-£38.63 to £110.81) |
| **Value of time off work** | | | | | |
| Time off work to attend physiotherapy | 598 | £12.90 (£38.99) | 1211 | £11.91 (£57.86) | £0.95 (-£3.81 to £5.70) |
| Time off work associated with the condition | 452 | £265.92 (£1350.82) | 884 | £226.61(£1139.84) | £111.31 (-£159.04 to £379.67) |

[a] Uses all available data, so denominators differ by category.

For peer review only



For peer review only - http://bmjopen.bmj.com/site/about/guidelines.xhtml

**Table 6: Cost-consequences. All available data[a].**

| | n (%) | usual care | n (%) | PhysioDirect | Incremental difference (95% Confidence Interval) |
|---|---|---|---|---|---|
| **Mean (SD) Cost** | | | | | |
| Total physiotherapy cost | 743 | £69.73 (£56.17) | 1506 | £74.01 (£63.97) | £4.28 (-£1.12 to £9.69) |
| Cost of NHS services including physiotherapy | 453 (61%) | £189.19 (£557.61) | 888 (59%) | £196.43 (£472.02) | £7.24 (-£49.68 to £64.10) |
| Total personal expenditure | 310 (42%) | £121.10 (£571.15) | 714 (47%) | £166.40 (£1040.27) | £45.30 (-£78.01 to £168.61) |
| Total value of all time off work[b] | 451 (61%) | £276.75 (£1355.00) | 883 (59%) | £240.74 (£1147.20) | -£36.01 (-£174.69 to £102.66) |
| **Consequences[c]** | | | | | **Difference/odds ratio (95% confidence interval)[d]** |
| SF36v2 PCS | 629 (85%) | 44.18 (10.84) | 1283 (85%) | 43.50 (10.94) | -0.01 (-0.80 to 0.79) |
| MYMOP[e] | 518 (70%) | 2.40 (1.38) | 1033 (69%) | 2.40 (1.43) | -0.02 (-0.16 to 0.11) |
| Global improvement score | 501 (67%) | 4.07 (1.40) | 1001 (66%) | 4.01 (1.44) | -0.08 (-0.23 to 0.08) |
| Response to treatment (OMERACT OARSI ) | 510 (69%) | 197 (38.6%) | 1029 (68%) | 430 (41.8%) | 1.14 (0.92 to 1.43) |
| Waiting time to first assessment and advice | 618 (83%) | 34 (20 to 55)[f] | 1281 (85%) | 7 (4 to 15)[f] | 0.32 (0.29 to 0.35)[g] |
| Patient overall satisfaction | 367 (49% | 79.7 (26.5) | 739 (49%) | 75.9 (28.3) | -3.8 (-7.3 to -0.3) |
| QALYs[h] | 454 (61%) | 0.322 (0.079) | 881 (58%) | 0.331 (0.082) | 0.009 (-0.000 to 0.018) |

[a] Uses all available data, so denominators differ by category
[b] Total of time off to attend physiotherapy and associated with the condition
[c] At 6 month follow-up time point
[d] Adjusted for outcome at baseline, gender, age, referral problem, PCT
[e] Lower score is better
[f] Median (IQR)
[g] Accelerated failure time analysis
[h] Adjusted for outcome at baseline

For peer review only

**Table 7: Cost-effectiveness analysis.** Includes cases with complete data on NHS costs and QALYs

| | n | usual care mean (SD) cost | n | PhysioDirect Mean (SD) cost | Incremental difference (95% CI) |
|---|---|---|---|---|---|
| **Cost of physiotherapy** | 432 | £78.77 (£57.08) | 840 | £86.75 (£65.47) | £7.98 (£0.69 to £15.27) |
| **Cost of NHS services other than physiotherapy** | 432 | £100.91 (£502.02) | 840 | £112.23 (£476.91) | £11.32 (-£45.08 to £67.72) |
| **Total cost including physiotherapy** | 432 | £179.68 (£504.73) | 840 | £198.98 (482.12) | £19.30 (-£37.60 to £76.19) |
| **QALYs** | 432 | 0.325 (0.077) | 840 | 0.332 (0.081) | 0.007 (-0.003 to 0.016) |
| **Incremental cost-effectiveness ratio (ICER)** | | | | | £2,889 |
| **Median Net Monetary Benefit (95% CI) based on bootstrapped results:** | | | | | |
| **λ=£20,000** | | | | | £117 (-£86 to £310) |
| probability of intervention being cost-effective | | | | | 0.88 |
| **λ=£30,000** | | | | | £184 (-£106 to £461) |
| probability of intervention being cost-effective | | | | | 0.90 |



For peer review only - http://bmjopen.bmj.com/site/about/guidelines.xhtml

**Table 8: Sensitivity Analysis**

| | n | Usual care | n | PhysioDirect | Incremental difference (95% confidence interval) |
|---|---|---|---|---|---|
| | | mean (SD) ~~Usual care~~ ~~mean (SD)~~ | | ~~PhysioDirect~~ mean (SD) | |
| **(1) mimicking an efficient service** | | | | | |
| Cost of physiotherapy | 432 | £76.56 (£55.34) | 840 | £72.22 (£61.55) | -£4.34 (-£11.25 to £2.57) |
| Cost of NHS services | 432 | £100.91 (£502.02) | 840 | £112.23 (476.91) | £11.32 (-£45.08 to £67.72 |
| Total cost | 432 | £177.46 (£504.49) | 840 | £184.44 (£481.83) | £6.98 (-£49.89 to £63.85) |
| Incremental cost-effectiveness ratio (ICER) | | | | | £1,045 |
| Median Net Monetary Benefit (95% CI) based on bootstrapped results: | | | | | |
| λ=£20,000 | | | | | £127 (-£74 to £319) |
| λ=£30,000 | | | | | £193 (-£95 to £473) |
| **(2) Excluding hospital costs** | | | | | |
| Cost of physiotherapy | 448 | £78.49 (£57.14) | 869 | £86.84 (£65.25) | £8.35 (£1.21 to £15.50) |
| Cost of NHS services excluding secondary care | 448 | £33.75 (£76.46) | 869 | £33.49 (£63.76) | -£0.25 (-£8.05 to £7.54) |
| Total cost of NHS services including physiotherapy | 448 | £112.23 (£99.621) | 869 | £120.33 (£98.85) | £8.10 (-£3.21 to £19.41) |
| Incremental cost-effectiveness ratio (ICER) | | | | | £1,084 |
| Median Net Monetary Benefit (95% CI) based on bootstrapped results: | | | | | |
| λ=£20,000 | | | | | £142 (-£41 to £324) |
| λ=£30,000 | | | | | £217 (-£56 to £489) |
| **(3) Imputed data** | | | | | |
| Cost of physiotherapy | 743 | £69.73 (£56.17) | 1506 | £74.01 (£63.97) | £4.28 (-£1.12 to £9.69) |
| Cost of NHS services | 743 | £131.37 (£465.49) | 1506 | £131.51 (£384.36) | £0.17 (-£36.13 to £36.48) |
| Total cost of NHS services including physiotherapy | 743 | £201.09 (£467.51) | 1506 | £205.55 (£390.04) | £4.46 (-£32.22 to £41.14) |
| Quality Adjusted Life Years | 743 | 0.320 (0.003) | 1506 | 0.322 (0.002) | 0.002 (-0.006 to 0.009) |
| Incremental cost-effectiveness ratio (ICER) | | | | | £2,260 |
| Median Net Monetary Benefit (95% CI) based on bootstrapped results: | | | | | |
| λ=£20,000 | | | | | £34 (-£119 to £193) |
| λ=£30,000 | | | | | £52 (-£172 to £285) |
| **(4) Imputed data and 'efficient' service** | | | | | |
| Cost of physiotherapy | 743 | £67.61 (£54.19) | 1506 | £61.41 (£59.13 | -£6.20 (-£11.26 to -£1.14) |
| Cost of NHS services | 743 | £131.37 (£465.49) | 1506 | £131.54 (384.36) | £0.17 (-£36.13 to £36.48) |
| Total cost of NHS services including physiotherapy | 743 | £198.98 (£467.48) | 1506 | £192.95 (£389.52) | -£6.02 (-£42.68 to £30.63) |
| Quality Adjusted Life Years | 743 | 0.320 (0.003) | 1506 | 0.322(0.002) | 0.002 (-0.006 to 0.009) |
| Incremental cost-effectiveness ratio (ICER) | | | | | -£3,054 |
| Median Net Monetary Benefit (95% CI) based on bootstrapped results: | | | | | |
| λ=£20,000 | | | | | £47 (-£113 to £202) |
| λ=£30,000 | | | | | £67 (-£165 to £293) |

Formatted ... [7]
Formatted Table ... [8]
Formatted ... [9]
Formatted ... [10]
Formatted ... [12]
Formatted ... [14]
Formatted ... [11]
Formatted ... [13]
Formatted ... [15]
Formatted ... [16]
Formatted ... [17]
Formatted ... [18]
Formatted ... [19]
Formatted ... [20]
Formatted ... [21]
Formatted ... [22]
Formatted ... [23]
Formatted ... [24]
Formatted ... [25]
Formatted ... [26]
Formatted ... [27]
Formatted ... [28]
Formatted ... [29]
Formatted ... [30]
Formatted ... [31]
Formatted ... [32]
Formatted ... [33]
Formatted ... [34]
Formatted ... [35]
Formatted ... [36]
Formatted ... [37]
Formatted ... [38]
Formatted ... [39]
Formatted ... [40]
Formatted ... [41]
Formatted ... [42]
Formatted ... [43]
Formatted ... [44]
Formatted ... [45]
Formatted ... [46]
Formatted ... [47]
Formatted ... [48]
Formatted ... [49]
Formatted ... [50]
Formatted ... [51]
Formatted ... [52]
Formatted ... [53]
Formatted ... [54]
Formatted ... [55]
Formatted ... [56]
Formatted ... [57]
Formatted ... [58]
Formatted ... [59]
Formatted ... [60]
Formatted ... [61]
Formatted ... [62]
Formatted ... [63]
Formatted ... [64]
Formatted ... [65]



For peer review only - http://bmjopen.bmj.com/site/about/guidelines.xhtml

For peer review only

40

**Figure 1. Cost-effectiveness acceptability curve showing the probability that the intervention is cost-effective at different levels of willingness to pay for one Quality Adjusted Life Year**

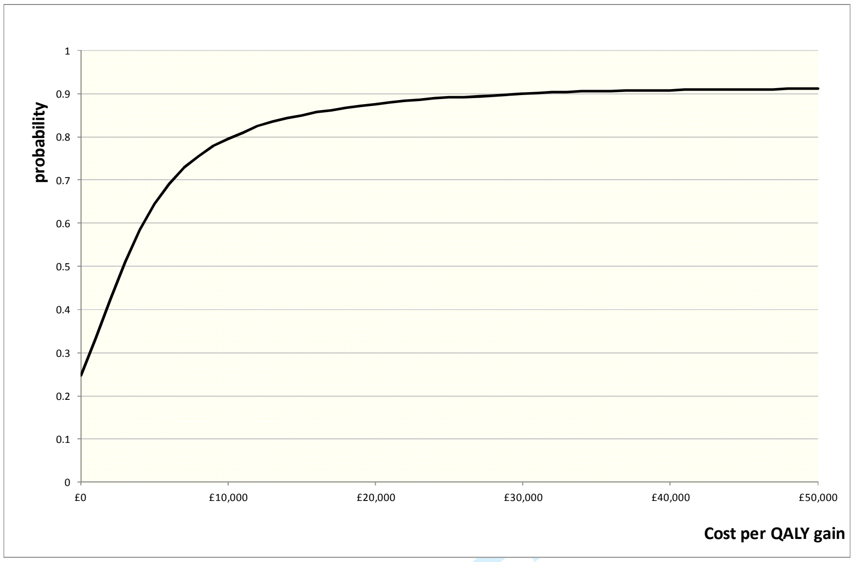


**Figure 2. Cost-effectiveness acceptability curve showing the probability that the intervention is cost-effective at different levels of willingness to pay for one Quality Adjusted Life Year: Sensitivity Analyses**

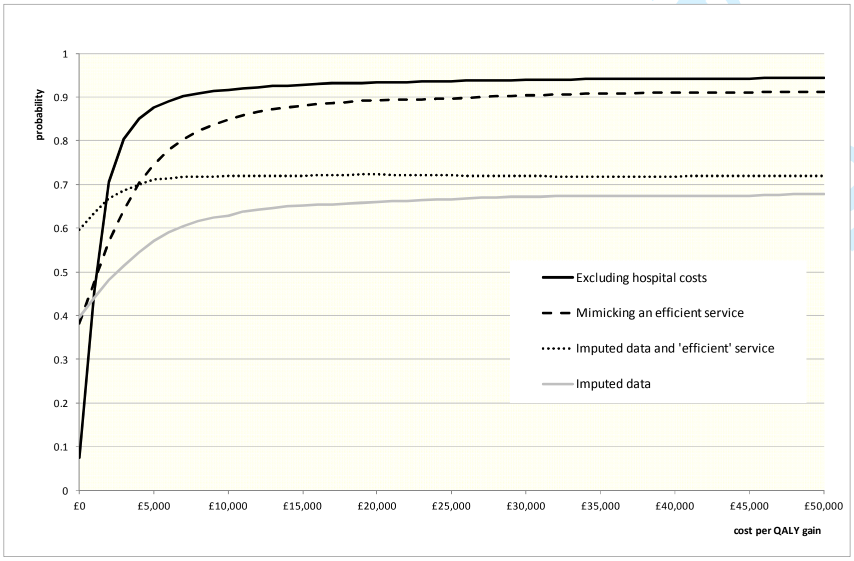




For peer review only - http://bmjopen.bmj.com/site/about/guidelines.xhtml

8/16/2013 11:42:00 AM

e same style, Line spacing: Multiple

8/16/2013 11:42:00 AM
le, Line spacing: Multiple 1.25 li

8/16/2013 11:42:00 AM
e same style, Line spacing: Multiple

8/16/2013 11:42:00 AM
le, Line spacing: Multiple 1.25 li

8/8/2013 5:03:00 PM
, Line spacing: Multiple 1.25 li

8/8/2013 5:03:00 PM
, Line spacing: Multiple 1.25 li

8/8/2013 5:03:00 PM
, Line spacing: Multiple 1.25 li

For peer review only

8/8/2013 5:03:00 PM

, Line spacing: Multiple 1.25 li

8/8/2013 5:02:00 PM

8/8/2013 5:02:00 PM

8/8/2013 5:03:00 PM

graphs of the same style, Line

8/8/2013 5:03:00 PM

, Line spacing: Multiple 1.25 li

8/8/2013 5:03:00 PM

, Line spacing: Multiple 1.25 li

8/8/2013 5:03:00 PM

, Line spacing: Multiple 1.25 li

8/8/2013 5:07:00 PM

For peer review only

, Line spacing: Multiple 1.25 li

8/8/2013 5:02:00 PM

8/8/2013 5:02:00 PM

8/8/2013 5:03:00 PM

hs of the same style, Line spacing:

8/8/2013 5:03:00 PM

graphs of the same style, Line

8/8/2013 5:03:00 PM

, Line spacing: Multiple 1.25 li

8/8/2013 5:03:00 PM

, Line spacing: Multiple 1.25 li

8/8/2013 5:03:00 PM

, Line spacing: Multiple 1.25 li

For peer review only

8/8/2013 5:03:00 PM

, Line spacing: Multiple 1.25 li

8/8/2013 5:06:00 PM

8/8/2013 5:06:00 PM

hs of the same style, Line spacing:

8/8/2013 5:03:00 PM

he same style, Line spacing: Multiple

8/8/2013 5:03:00 PM

, Line spacing: Multiple 1.25 li

8/8/2013 5:03:00 PM

, Line spacing: Multiple 1.25 li

8/8/2013 5:03:00 PM

, Line spacing: Multiple 1.25 li

8/8/2013 5:03:00 PM

For peer review only

For peer review only

**CHEERS checklist—Items to include when reporting economic evaluations of health interventions**

| Section/item | Item No | Recommendation | Reported on page No/ line No |
|---|---|---|---|
| **Title and abstract** | | | |
| Title | 1 | Identify the study as an economic evaluation or use more specific terms such as "cost-effectiveness analysis", and describe the interventions compared. | 1/1-3 |
| Abstract | 2 | Provide a structured summary of objectives, perspective, setting, methods (including study design and inputs), results (including base case and uncertainty analyses), and conclusions. | P2-3 |
| **Introduction** | | | |
| Background and objectives | 3 | Provide an explicit statement of the broader context for the study. Present the study question and its relevance for health policy or practice decisions. | 5/1 – 6/20 |
| **Methods** | | | |
| Target population and subgroups | 4 | Describe characteristics of the base case population and subgroups analysed, including why they were chosen. | 7/14-18 |
| Setting and location | 5 | State relevant aspects of the system(s) in which the decision(s) need(s) to be made. | 7/14-18 |
| Study perspective | 6 | Describe the perspective of the study and relate this to the costs being evaluated. | 7/1-11 |
| Comparators | 7 | Describe the interventions or strategies being compared and state why they were chosen. | 7/22 - 8/5 |
| Time horizon | 8 | State the time horizon(s) over which costs and consequences are being evaluated and say why appropriate. | 6/25 - 7/1 |
| Discount rate | 9 | Report the choice of discount rate(s) used for costs and outcomes and say why appropriate. | 13/14-15 |
| Choice of health outcomes | 10 | Describe what outcomes were used as the measure(s) of benefit in the evaluation and their relevance for the type of analysis performed. | 8/8-16 |
| Measurement of effectiveness | 11a | *Single study-based estimates:* Describe fully the design features of the single effectiveness study and why the single study was a sufficient source of clinical effectiveness data. | 6/25-7/1 7/14-19 |
| | 11b | *Synthesis-based estimates:* Describe fully the methods used for identification of included studies and synthesis of clinical effectiveness data. | n/a |

For peer review only

| | | | |
|---|---|---|---|
| Measurement and valuation of preference based outcomes | 12 | If applicable, describe the population and methods used to elicit preferences for outcomes. | 8/8-9<br>12/11-12 |
| Estimating resources and costs | 13a | *Single study-based economic evaluation:* Describe approaches used to estimate resource use associated with the alternative interventions. Describe primary or secondary research methods for valuing each resource item in terms of its unit cost. Describe any adjustments made to approximate to opportunity costs. | 9/19 – 12/2 |
| | 13b | *Model-based economic evaluation:* Describe approaches and data sources used to estimate resource use associated with model health states. Describe primary or secondary research methods for valuing each resource item in terms of its unit cost. Describe any adjustments made to approximate to opportunity costs. | n/a |
| Currency, price date, and conversion | 14 | Report the dates of the estimated resource quantities and unit costs. Describe methods for adjusting estimated unit costs to the year of reported costs if necessary. Describe methods for converting costs into a common currency base and the exchange rate. | 12/4 |
| Choice of model | 15 | Describe and give reasons for the specific type of decision-analytical model used. Providing a figure to show model structure is strongly recommended. | n/a |
| Assumptions | 16 | Describe all structural or other assumptions underpinning the decision-analytical model. | n/a |
| Analytical methods | 17 | Describe all analytical methods supporting the evaluation. This could include methods for dealing with skewed, missing, or censored data; extrapolation methods; methods for pooling data; approaches to validate or make adjustments (such as half cycle corrections) to a model; and methods for handling population heterogeneity and uncertainty. | 12/12-15<br>13/8-12 |
| **Results** | | | |
| Study parameters | 18 | Report the values, ranges, references, and, if used, probability distributions for all parameters. Report reasons or sources for distributions used to represent uncertainty where appropriate. Providing a table to show the input values is strongly recommended. | n/a |
| Incremental costs and outcomes | 19 | For each intervention, report mean values for the main categories of estimated costs and outcomes of interest, as well as mean differences between the comparator groups. If applicable, report incremental cost-effectiveness ratios. | 15/17-16/11<br>Tables 5,6,7 |

For peer review only

| | | | |
|---|---|---|---|
| Characterising uncertainty | 20a | *Single study-based economic evaluation:* Describe the effects of sampling uncertainty for the estimated incremental cost and incremental effectiveness parameters, together with the impact of methodological assumptions (such as discount rate, study perspective). | 16/13-21<br>Table 7<br>Figure 1<br>16/26 – 18/5<br>Table 8<br>Figure 2 |
| | 20b | *Model-based economic evaluation:* Describe the effects on the results of uncertainty for all input parameters, and uncertainty related to the structure of the model and assumptions. | n/a |
| Characterising heterogeneity | 21 | If applicable, report differences in costs, outcomes, or cost-effectiveness that can be explained by variations between subgroups of patients with different baseline characteristics or other observed variability in effects that are not reducible by more information. | n/a |
| **Discussion** | | | |
| Study findings, limitations, generalisability, and current knowledge | 22 | Summarise key study findings and describe how they support the conclusions reached. Discuss limitations and the generalisability of the findings and how the findings fit with current knowledge. | 18/9 – 19/25 |
| **Other** | | | |
| Source of funding | 23 | Describe how the study was funded and the role of the funder in the identification, design, conduct, and reporting of the analysis. Describe other non-monetary sources of support. | 28/6-11 |
| Conflicts of interest | 24 | Describe any potential for conflict of interest of study contributors in accordance with journal policy. In the absence of a journal policy, we recommend authors comply with International Committee of Medical Journal Editors recommendations. | 28/1-3 |

**For consistency, the CHEERS statement checklist format is based on the format of the CONSORT statement checklist**

For peer review only
